# Formation of *N*-Hydroxyethylisoindolinone Derivatives in Fungi Requires Highly Coordinated Consecutive Oxidation Steps

**DOI:** 10.1021/acs.orglett.5c00328

**Published:** 2025-03-03

**Authors:** Zhang-Hai Li, Yu Dai, Jing Zhou, Li Yang, Shu-Ming Li

**Affiliations:** † Institut für Pharmazeutische Biologie und Biotechnologie, Fachbereich Pharmazie, Philipps-Universität Marburg, Robert-Koch-Straße 4, 35037 Marburg, Germany; ‡ Key Laboratory of Tropical Biological Resources of Ministry of Education, School of Pharmaceutical Sciences, Hainan University, 570200 Haikou, P. R. China; § Haikou Key Laboratory for Research and Utilization of Tropical Natural Products and National Key Laboratory for Tropical Crop Breeding, Institute of Tropical Bioscience and Biotechnology, Chinese Academy of Tropical Agricultural Sciences, 571101 Haikou, P. R. China

## Abstract

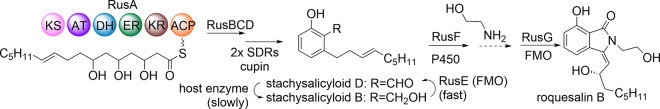

Gene duplication significantly contributes to the diversification of biosynthetic potential and increases the structural diversity of secondary metabolites. Here, we report the second alkyl salicylaldehyde derivative biosynthetic gene cluster in *Penicillium roqueforti*, being responsible for the formation of ethanolamine-containing derivatives. Heterologous expression and feeding experiments provided evidence for their formation via collaboration and modification with one cytochrome P450 and two flavin-containing monooxygenases in a highly ordered manner before and after ethanolamine incorporation.

Alkyl salicylaldehyde derivatives (ASDs) make up a structurally diverse group of fungal metabolites.^1−4^ Some of them exhibit interesting biological and pharmacological properties.^5,6^ Their scaffolds are benzylic alcohol, benzoic aldehyde, or acid with a hydroxyl group and an alkyl chain at its *ortho*-positions. The alkyl chains have various saturation grades and different modifications (Figure 1).^1−3,7,8^ Recent investigations revealed that they are products of strongly reducing polyketide synthases (HR-PKSs),^8,9^ clearly differing from most aromatic polyketides by nonreducing or partially reducing polyketide synthases.^10,11^ For the HR-PKS-catalyzed formation of ASDs, two short-chain dehydrogenases/reductases (SDRs) and one cupin domain-containing protein are usually necessary.^9,12^ The scaffolds undergo further modifications like oxidation and prenylation (Figure 1).^3,7,8,13^ Examples are trichoxide after oxidation,^8^ flavoglaucin and its congeners after prenylation and oxidation,^7^ and annullatins after hydroxylation, prenylation, and lactone formation.^13^

**Figure 1:**
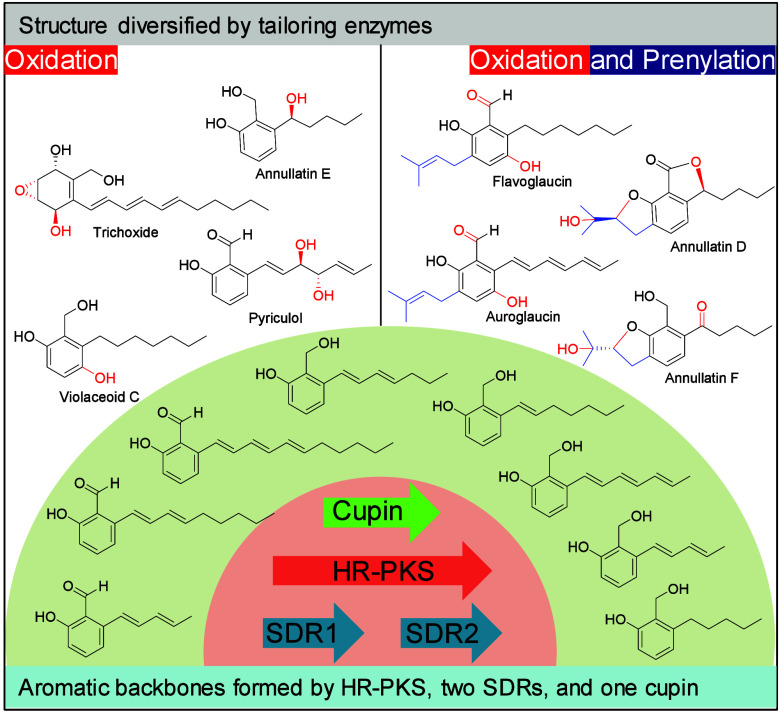
Structures of alkyl salicylaldehyde or salicylalcohol scaffolds as products of the four essential enzymes and their derivatives.

A blast search with the four proteins in Figure 1 revealed the presence of at least 493 homologous clusters in 401 fungal strains (Figure S1), including two in *Penicillium roqueforti* FM164 (Figure S1). They contain four genes with significant sequence similarities to those highlighted in Figure 1. One of them has been confirmed to be responsible for annullatin biosynthesis,^13^ while the products of another, here termed the *rus* cluster, remain unknown. The *rus* cluster spans base pairs 830 087–853 119 of HG792020.1 and contains eight putative genes encoding one HR-PKS (RusA), two SDRs (RusB and RusD), two flavin-containing monooxygenases (FMOs, RusE and RusG), one cupin domain-containing protein (RusC), one cytochrome P450 (RusF), and one transcription factor RusH (Figure 2A). RusA–D share sequence identities of 51.1–63.7% with their homologues from the *str* cluster^9^ and 43.2–60.1% with those from the *anu* cluster (Table S1 and Figure S2).^13^

**Figure 2:**
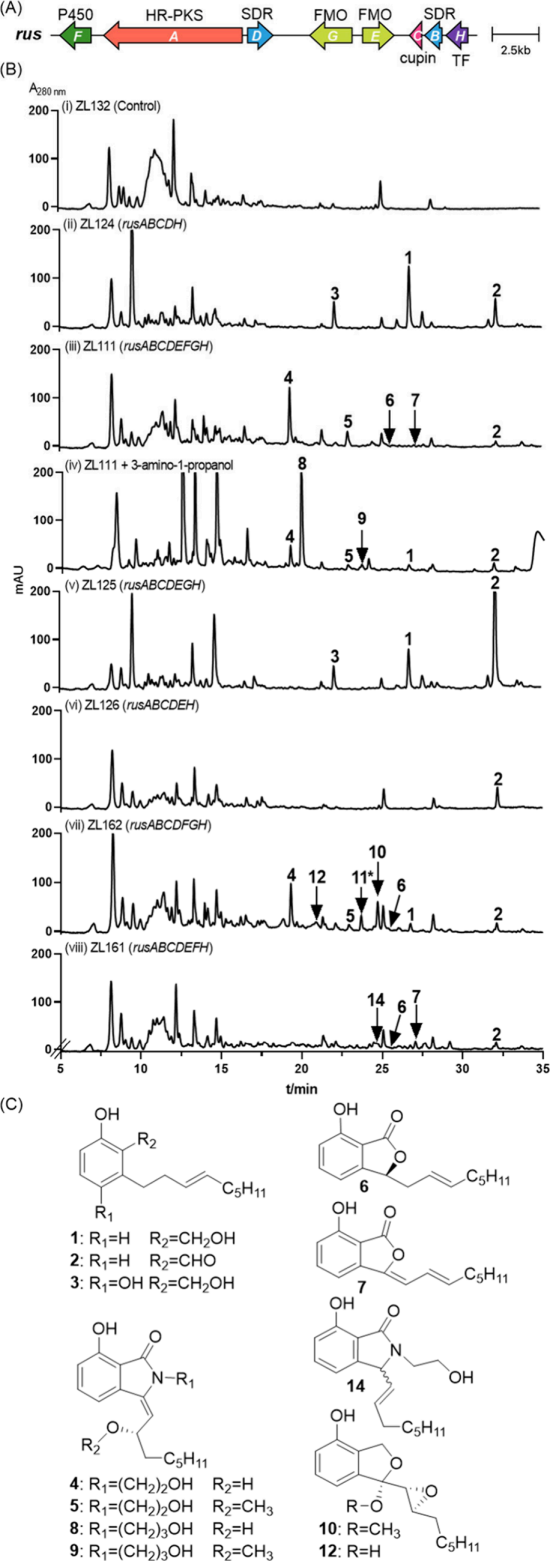
(A) *rus* BGC from *P. roqueforti* FM164. (B) HPLC analysis of *A. nidulans* transformant extracts after cultivation for 14 days in rice media. (C) Structures of the isolated compounds.

We first constructed a plasmid containing *rusA*–*D* and transcription factor gene *rusH* for heterologous expression. In this construct, *rusA*, *rusD*, and *rusH* are placed under the control of different constitutive promoters P*gpdA*, P*hlyA*, and P*gpdA*^*#*^ (*gpdA* promoter originated from *Aspergillus niger*), respectively (Table S3). Protoplast transformation into *Aspergillus nidulans* LO8030 resulted in strain ZL124 (Table S5 and Figure S3). LC-MS analysis of an EtOAc extract of a 14-day-old rice culture revealed the presence of three new peaks, **1**–**3**, with **1** as the main product, which were absent in control strain ZL132 harboring empty vector pZL132 (Table S3 and Figure 2Bi,ii). Isolation and structural elucidation (see the Supporting Information for details) confirmed **1** and **2** as stachysalicyloids B and D, respectively (Table S6 and Figure 4), two products of the *str* cluster.^9^ New compound **3** with an additional hydroxy group at the benzene ring of **1** was termed roquesalin A (Table S6 and Figure 4). These results confirmed that *rusA*–*D* are also responsible for ASD formation.

To further characterize the pathway products, *rusA*–*H* were cloned into pZL132 by homologous recombination^14^ and then integrated into the LO8030 genome. In analogy to ZL124, *rusA*, *rusD*, and *rusH* were placed under the control of the aforementioned promoters. Resulting transformant ZL111 (Table S5 and Figure 2Biii) was subsequently cultivated in a rice medium. LC-MS analysis revealed the presence of a predominant peak, **4**, with a [M + H]^+^ ion at *m*/*z* 320.1867 (C_18_H_26_NO_4_). Isolation and structural elucidation proved it to be an *N*-hydroxyethylisoindolinone derivative by incorporation of an ethanolamine into the alkyl salicylaldehyde scaffold, termed roquesalin B (Table S6 and Figure 4). By comparison of the experimental ECD data with the calculated values, the configuration of **4** was assigned to be 10*S* (Tables S10 and S11 and Figure S63). In addition, three minor peaks, **5**–**7**, were also detected (Figure 2Biii). Compound **5** is a methylated product of **4** (Table S7 and Figure 2C). Given that the construct contains no methyltransferase gene, it is plausible that **5** is formed by the involvement of a host enzyme or by a non-enzymatic reaction. The other two minor products, **6** and **7**, are phthalide derivatives that lack ethanolamine (Table S7 and Figure 2C). They differ from each other by the number of double bonds at the alkyl chain.

Natural products with an *N*-hydroxyethylisoindolinone core structure were identified in different microbials, but their biosynthesis, especially the mechanism of coupling of ethanolamine with aromatic core structure, has not been reported.^15−18^

Addition of 150 mM 3-amino-1-propanol to a 5-day-old rice culture of strain ZL111 led to detection of a new predominant peak, **8**, and a minor peak, **9**, with [M + H]^+^ ions at *m*/*z* 334.2023 and 348.2179, respectively, 14 Da more than those of **4** and **5**, respectively (Figure 2Biv). This indicates the incorporation of free 3-amino-1-propanol into their structures, which was also confirmed by NMR analysis (Tables S7 and S8 and Figure 4). These results strongly support our hypothesis that free ethanolamine was directly used for **4** and **5** formation.

To investigate the biosynthetic route to **4**, we designed three plasmids, pZL125, pZL161, and pZL162 (Table S3), containing the *rus* cluster without *rusF*, *rusG*, and *rusE*, respectively. These plasmids were subsequently transformed into LO8030 to generate expression strains ZL125, ZL161, and ZL162, respectively (Table S5 and Figure S3). When *rusF* encoding the P450 enzyme was excluded, the production of **4**–**7** was abolished. Instead, compound **2** was detected as a predominant peak (Figure 2Bv). This suggests that one or both of the FMOs RusE and RusG can oxidize **1** to **2**.

To elucidate the function of RusE and RusG, we fed **1** to the 5-day-old culture of *rusE* expression strain ZL164, resulting in almost complete conversion to **2** after 3 days (Figure 3iii). In contrast, **2** was detected as a minor peak in RusG expression strain ZL169 and control strain ZL132 (Figure 4ii,iv). Compound **3** was detected in all three strains, indicating the involvement of a host enzyme in **1** hydroxylation (Figure 4). These results proved that RusE is responsible for the conversion of **1** to **2** (Figure 4), which is consistent with the accumulation of **2** as the main product in strain ZL126 (Figure 2Bvi). Lower but clearly detectable conversion of **1** to **2** in strains ZL132 and ZL169 proved that a host enzyme can partially complement the RusE function.

**Figure 3:**
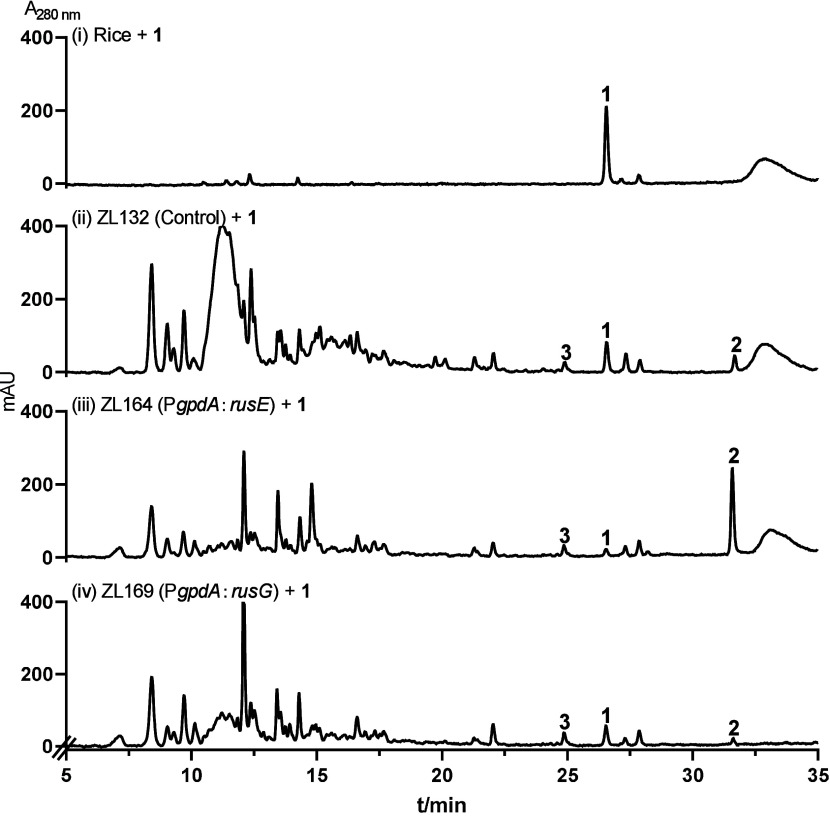
HPLC analysis of *A. nidulans* cultures after they had been fed **1**. Control strain ZL132 harbors the empty vector.

**Figure 4:**
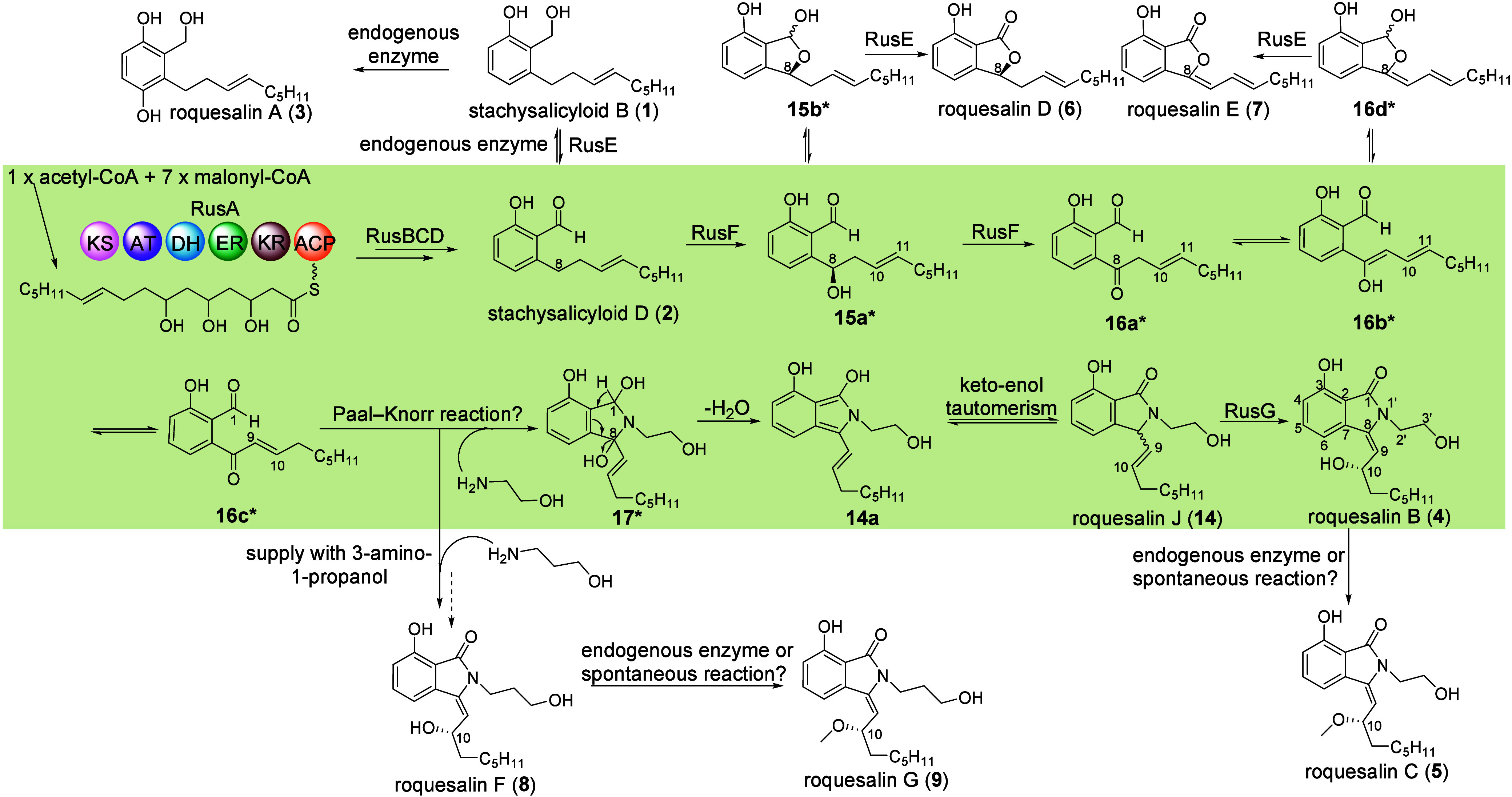
Proposed biosynthetic pathways of the roquesalins. The main pathway is highlighted with a beige background.

Expression of the cluster without *rusE* resulted in strain ZL162, with **4**, **10**, and 11* as the main products as well as **1**, **2**, **5**, **6**, and **12** as the minor products (Figure 2Bvii). Three new peaks **10**, 11*, and **12** with almost the same UV spectra showed [M + Na]^+^ ions at *m*/*z* 315.1575, 315.1579, and 301.1416, respectively, suggesting that **12** differs from **10** and 11* by lacking a methyl group. **10** was proven to be a ketal, and **12** its hemiketal congener (Tables S8 and S9 and Figure S67). Differing from phthalides **6** and **7**, they contain an isobenzofuran-3-ol scaffold with an epoxy group between C-9 and C-10 of the side chain. Compound 11* was instable and converted in part into **12** during the isolation. In this Letter, we use an asterisk after a compound number for proposed structures, which were not verified by NMR analysis. Incubation of **12** in CD_3_OH and H_2_^18^O confirmed that **10** and 11* are methylated derivatives of **12** and its keto form **12a**, respectively. Conversion between 11* and **12a** is a spontaneous event via a 1,4-Michael addition with *o*-quinone methide 13* (Figures S65–S67). Such phenomena were also observed for hydroxyclavatol and its methylated derivative in a previous study.^19^

Expression of the *rus* cluster without *rusG* encoding the second FMO led to a low level of product formation in transformant ZL161, with **2**, **7**, and **14** as the major products in comparable intensities. In addition, compound **6** was identified as a minor product (Figure 2Bviii). In comparison to those of **1**–**3**, an oxygen function is installed at C-8 in **6** and **7**, indicating a possible function of RusF as a hydroxylase. Compound **14** is an analogue of **4** with an integrated ethanolamine moiety (Figure 2C). They differ from each other by a hydroxyl group at C-10 and a double bond between C-8 and C-9 in the structure of **4** (Table S9). These results suggested that after the oxidation at C-8 by the membrane-bound P450 enzyme, the widely distributed ethanolamine in the cell membrane^20^ can be loaded onto the alkyl salicylaldehyde scaffold. Upon comparison of product **14** obtained after *rusABCDEFH* expression (Figure 2Bviii) with **4** and **5** after whole cluster expression, it is obvious that the latter compounds have additional hydroxyl/methoxyl groups at C-10, implying the FMO RusG as the last pathway enzyme for introduction of this oxygen function (Figure S68). This would also be in agreement with **10** and **12** obtained from strain ZL162 without RusE.

Based on the results described above, we postulate a biosynthetic pathway for roquesalin B by incorporation of ethanolamine into alkyl salicylaldehyde (Figure 4). The roquesalin backbone is assembled by cooperation of RusABCD and released as aldehyde **2**, which can be reduced to alcohol **1** by an unknown host enzyme. The FMO RusE oxidizes **1** back to **2** to ensure the oxidation state of the backbone for further reactions. Further oxidation at C-8 of the alkyl chain by cytochrome P450 RusF in two steps formed 8-hydroxylated intermediate 15a* and 8-keto 16a*. The double bond shift from C-10 and C-11 in 16a* to C-9 and C-10 in 16c* is likely caused by keto–enol tautomerism via intermediate 16b*. Spontaneous electrophilic attack of the aldehyde C-1 and the keto C-8 in 16c* by the amino group of ethanolamine via a Paal–Knorr reaction^21^ would result in intermediate 17*. Subsequently, water elimination between C-1 and C-8 leads to isoindole **14a**. Compound **14** as an enantiomeric pair is then formed by keto–enol tautomerism. Hydroxylation at C-10 catalyzed by the FMO RusG completes the biosynthesis of major pathway product **4**, which is then methylated in part to **5** by a host enzyme or non-enzymatic reaction as discussed above. The pathway to **4** differs clearly from the incorporation of an ethanolamine moiety into the structures of pyxidicyclines.^18^ In that pathway, the ethanolamine moiety is formed by the decarboxylation of serine after coupling with an anthracene carboxylic acid.

The formation of main product **4** of the Rus pathway (Figure 4) requires a highly coordinated reaction order. Only in the presence of all three tailoring enzymes RusE–G can the product of RusA–D be effectively converted into main product **4** (Figure 2Biii). Feeding of 3-amino-1-propanol to this strain also led to the accumulation of its congener **8** as the predominant product (Figure 2Biv).

Without RusE, a higher level of accumulation of **1** can be expected, which was then converted into three epoxylated derivatives, **10**–**12**, in high yields (Figure 2Bvii). This proved the essential role of the aryl aldehyde group in the coupling with ethanolamine. Obviously, **1** can also serve as a substrate for RusF to yield C8-oxidized product 18a*, which undergoes subsequent rearrangement to 18b* as mentioned above. The action of RusG on the double bond leads to the formation of epoxylated derivative **12a** in substantial amounts, which was isolated as methyl ketal **10** and hemiketal **12** (Figure 2Bvii and Figure S67).

Without the P450 enzyme RusF for oxidation at C-8, significant accumulation of **2** was observed in ZL126. The involvement of *rusG* in strain ZL125 significantly increased the yields of **2** (Figure 2Bv,vi). In strain ZL161 expressing *rusA–F* and *rusH*, but lacking *rusG*, very low product yields for **6**, **7**, and **14** were observed (Figure 2Bviii). These proved that RusG not only is for the oxidation at C-10 but also plays an important role to direct the pathway flow and to increase product yields, which is also in agreement with the high yield of **2** in strain ZL125. Compounds **6** and **7** in trace amounts were also detected in expression strain ZL111 with the whole cluster (Figure 2Biii). They are likely formed by RusE oxidation of hemiacetals 15b* and 16d* (Figure 4), which is also in agreement with its role in **1** oxidation. Compound **14** is an analogue of **4** lacking the hydroxyl group at C-10 (Figure 4). Accumulation of **14** in ZL161 supports the role of RusG in the oxidation at C-10. Formation of **10**–**12** in ZL162 also implies its action on the double bond of structures without the ethanolamine moiety.

In conclusion, we identified a biosynthetic gene cluster for roquesalins in *P. roqueforti* and elucidated their biosynthetic pathway by heterologous expression and feeding experiments. We provide herewith an example for the biosynthetic machinery of highly coordinated consecutive reaction steps. To the best of our knowledge, the biosynthetic pathway for the formation of ethanolamine-containing ASDs has not been reported prior to this study. Genome mining and analysis by using cblaster^22^ identified at least 18 *rus*-homologous clusters in other fungal species (Figure S69). These clusters could also be responsible for the formation of ethanolamine-containing ASDs.

## Data Availability

The data underlying this study are available in the published article and its Supporting Information.

## Supporting Information Available

Additional experimental details and materials and methods, including strains, physiochemical properties, LC-MS, and NMR data (PDF)
