# Supplementary material for: Formation of N-Hydroxyethylisoindolinone Derivatives in Fungi Requires Highly Coordinated Consecutive Oxidation Steps
Source: Org Lett. 2025 Mar 3;27(10):2433–7. doi: 10.1021/acs.orglett.5c00328 (PMC11915489; doi:10.1021/acs.orglett.5c00328)
Supplement: Supplementary file 1 — ol5c00328_si_001.pdf [file ol5c00328_si_001.pdf]

Supporting Information

Formation of *N*-Hydroxyethylisoindolinone Derivatives in Fungi Requires Highly Coordinated Consecutive Oxidation Steps

Zhang-Hai Li,[‡a] Yu Dai,[‡a] Jing Zhou,[‡b] Li Yang,[c] and Shu-Ming Li[a*]

[a]Institut für Pharmazeutische Biologie und Biotechnologie, Fachbereich Pharmazie, Philipps-Universität Marburg, Robert-Koch-Straße 4, 35037 Marburg, Germany

[b]Key Laboratory of Tropical Biological Resources of Ministry of Education, School of Pharmaceutical Sciences, Hainan University, Haikou 570200, P. R. China

[c]Haikou Key Laboratory for Research and Utilization of Tropical Natural Products & National Key Laboratory for Tropical Crop Breeding, Institute of Tropical Bioscience and Biotechnology, Chinese Academy of Tropical Agricultural Sciences, Haikou 571101, P. R. China

‡These authors contributed equally

*Corresponding author. Email: shuming.li@staff.uni-marburg.de

Table of Contents

Experiment Procedures 6

1. Media, strains, and growth conditions 6
2. Genomic DNA isolation 6
3. Sequence analysis 6
4. PCR amplification, gene cloning, and plasmid construction 7
5. Heterologous expression in *A. nidulans* 7
6. LC-HRMS analysis for secondary metabolites 7
7. Cultivation of *A. nidulans* ZL111 with 3-amino-1-propanol 7
8. Large-scale fermentation, extraction, and isolation of secondary metabolites 8
9. NMR analysis 8
10. Structural elucidation 9
11. Precursor supply for *A. nidulans* ZL164 and ZL169 11
12. Proof of the conversion of **12** to **10** and **11*** 11
13. Measurement of optical rotations 11
14. The experimental and calculated electronic circular dichroism (ECD) spectroscopic analysis 12
15. Physiochemical properties of the compounds described in this study 12

Supplementary Tables 14

Table S1. Sequence identities of proteins encoded by the *rus* cluster from *Penicillium roqueforti* FM164 with those of known clusters. 14

Table S2. Primers used in this study. 15

Table S3. Plasmids used and constructed in this study. 16

Table S4. Media and solutions used in this study. 18

Table S5. Strains used in this study. 19

Table S6. ^{1}H (500 MHz) and ^{13}C NMR (125 MHz) spectroscopic data of stachysalicyloid B (**1**), stachysalicyloid D (**2**), roquesalin A (**3**) in $CDCl_3$, and roquesalin B (**4**) in DMSO-d_6 with key HMBC and ^{1}H-^{1}H COSY correlations. 20

Table S7. ^{1}H (500 MHz) and ^{13}C NMR (125 MHz) spectroscopic data of roquesalin C (**5**) and in DMSO-d_6, and roquesalin D (**6**), roquesalin E (**7**), roquesalin F (**8**) in $CDCl_3$ with key HMBC and ^{1}H-^{1}H COSY correlations. 21

Table S8. ^{1}H (500 MHz) and ^{13}C NMR (125 MHz) spectroscopic data of roquesalin G (**9**) in DMSO-d_6, and roquesalin H (**10**) in $CDCl_3$ with key HMBC and ^{1}H-^{1}H COSY correlations. 22

Table S9. ^{1}H (500 MHz) and ^{13}C NMR (125 MHz) spectroscopic data of roquesalin I (**12**) in $CDCl_3$ and roquesalin J (**14**) DMSO-d_6 in with key HMBC and ^{1}H-^{1}H COSY correlations. 23
Table S10. Conformational analysis of the optimized isomers of **4** in methanol. 23
Table S11. The coordinates of the optimized conformers of **4**. 24
Table S12. Experimental and calculated ^{13}C NMR chemical shifts of **12**. 26
Table S13. Experimental and calculated ^{1}H NMR chemical shifts of **12**. 27
Supplementary Figures 28
Figure S1. Results of the cblaster analysis against the RefSeq database using the sequences of FogABCD as queries. 28
Figure S2. Schematic representation of the *rus* cluster and its homologous gene clusters. 28
Figure S3. Heterologous expression and PCR verification for *A. nidulans* strains. 29
Figure S4. ^{1}H NMR spectrum of stachysalicyloid B (**1**) in $CDCl_3$ (500 MHz). 30
Figure S5. ^{13}C NMR spectrum of stachysalicyloid B (**1**) in $CDCl_3$ (125 MHz). 31
Figure S6. HSQC spectrum of stachysalicyloid B (**1**) in $CDCl_3$. 32
Figure S7. ^{1}H-^{1}H COSY spectrum of stachysalicyloid B (**1**) in $CDCl_3$. 33
Figure S8. HMBC spectrum of stachysalicyloid B (**1**) in $CDCl_3$. 34
Figure S9. ^{1}H NMR spectrum of stachysalicyloid D (**2**) in $CDCl_3$ (500 MHz). 35
Figure S10. ^{13}C NMR spectrum of stachysalicyloid D (**2**) in $CDCl_3$ (125 MHz). 36
Figure S11. ^{1}H NMR spectrum of roquesalin A (**3**) in $CDCl_3$ (500 MHz). 37
Figure S12. ^{13}C NMR spectrum of roquesalin A (**3**) in $CDCl_3$ (125 MHz). 38
Figure S13. HSQC spectrum of roquesalin A (**3**) in $CDCl_3$. 39
Figure S14. ^{1}H-^{1}H COSY spectrum of roquesalin A (**3**) in $CDCl_3$. 40
Figure S15. HMBC spectrum of roquesalin A (**3**) in $CDCl_3$. 41
Figure S16. ^{1}H NMR spectrum of roquesalin B (**4**) in DMSO-d_6 (500 MHz). 42
Figure S17. ^{13}C NMR spectrum of roquesalin B (**4**) in DMSO-d_6 (125 MHz). 43
Figure S18. HSQC spectrum of roquesalin B (**4**) in DMSO-d_6. 44
Figure S19. ^{1}H-^{1}H COSY spectrum of roquesalin B (**4**) in DMSO-d_6. 45
Figure S20. HMBC spectrum of roquesalin B (**4**) in DMSO-d_6. 46
Figure S21. ^{1}H-^{1}H NOESY spectrum of roquesalin B (**4**) in DMSO-d_6. 47
Figure S22. ^{1}H NMR spectrum of roquesalin C (**5**) in DMSO-d_6 (500 MHz). 48
Figure S23. ^{13}C NMR spectrum of roquesalin C (**5**) in DMSO-d_6 (125 MHz). 49
Figure S24. HSQC spectrum of roquesalin C (**5**) in DMSO-d_6. 50

Figure S25. ^{1}H-^{1}H COSY spectrum of roquesalin C (**5**) in DMSO-d_6. 51
Figure S26. HMBC spectrum of roquesalin C (**5**) in DMSO-d_6. 52
Figure S27. ^{1}H NMR spectrum of roquesalin D (**6**) in $CDCl_3$ (500 MHz). 53
Figure S28. ^{13}C NMR spectrum of roquesalin D (**6**) in $CDCl_3$ (125 MHz). 54
Figure S29. HSQC spectrum of roquesalin D (**6**) in $CDCl_3$. 55
Figure S30. ^{1}H-^{1}H COSY spectrum of roquesalin D (**6**) in $CDCl_3$. 56
Figure S31. HMBC spectrum of roquesalin D (**6**) in $CDCl_3$. 57
Figure S32. ^{1}H NMR spectrum of roquesalin E (**7**) in $CDCl_3$ (500 MHz). 58
Figure S33. ^{13}C NMR spectrum of roquesalin E (**7**) in $CDCl_3$ (125 MHz). 59
Figure S34. HSQC spectrum of roquesalin E (**7**) in $CDCl_3$. 60
Figure S35. ^{1}H-^{1}H COSY spectrum of roquesalin E (**7**) in $CDCl_3$. 61
Figure S36. HMBC spectrum of roquesalin E (**7**) in $CDCl_3$. 62
Figure S37. ^{1}H-^{1}H NOESY spectrum of roquesalin E (**7**) in $CDCl_3$. 63
Figure S38. ^{1}H NMR spectrum of roquesalin F (**8**) in $CDCl_3$ (500 MHz). 64
Figure S39. ^{1}H NMR spectrum of roquesalin G (**9**) in DMSO-d_6 (500 MHz). 65
Figure S40. ^{13}C NMR spectrum of roquesalin G (**9**) in DMSO-d_6 (125 MHz). 66
Figure S41. HSQC spectrum of roquesalin G (**9**) in DMSO-d_6. 67
Figure S42. HMBC spectrum of roquesalin G (**9**) in DMSO-d_6. 68
Figure S43. ^{1}H NMR spectrum of roquesalin H (**10**) in $CDCl_3$ (500 MHz). 69
Figure S44. ^{13}C NMR spectrum of roquesalin H (**10**) in $CDCl_3$ (125 MHz). 70
Figure S45. HSQC spectrum of roquesalin H (**10**) in $CDCl_3$. 71
Figure S46. ^{1}H-^{1}H COSY spectrum of roquesalin H (**10**) in $CDCl_3$. 72
Figure S47. HMBC spectrum of roquesalin H (**10**) in $CDCl_3$. 73
Figure S48. ^{1}H NMR spectrum of roquesalin I (**12**) in $CDCl_3$ (500 MHz). 74
Figure S49. ^{13}C NMR spectrum of roquesalin I (**12**) in $CDCl_3$ (125 MHz). 75
Figure S50. HSQC spectrum of roquesalin I (**12**) in $CDCl_3$. 76
Figure S51. ^{1}H-^{1}H COSY spectrum of roquesalin I (**12**) in $CDCl_3$. 77
Figure S52. HMBC spectrum of roquesalin I (**12**) in $CDCl_3$. 78
Figure S53. NOESY spectrum of roquesalin I (**12**) in $CDCl_3$. 79
Figure S54. ^{1}H NMR spectrum of roquesalin J (**14**) in DMSO-d_6 (500 MHz). 80
Figure S55. ^{13}C NMR spectrum of roquesalin J (**14**) in DMSO-d_6 (125 MHz). 81
Figure S56. HSQC spectrum of roquesalin J (**14**) inDMSO-d_6. 82
Figure S57. ^{1}H-^{1}H COSY spectrum of roquesalin J (**14**) in DMSO-d_6. 83

Figure S58. HMBC spectrum of roquesalin J (**14**) in DMSO-d_6. 84

Figure S59. The UV spectra of the identified compounds. 85

Figure S60. The mass spectra of the identified compounds. 86

Figure S61. Linear regression analysis of experimental and calculated NMR chemical shifts of isomers of **12**. 87

Figure S62. The calculated ECD spectra of (8*S*,9*S*,10*R*)- and (8*R*,9*R*,10*S*)-12, and experimental ECD spectra of **10** and **12**. 87

Figure S63. The experimental spectra of **4**, **5**, **8**, and **9**, and calculated ECD spectra of **4**. 88

Figure S64. The experimental spectrum of **6**, and calculated ECD spectra of **6**. 88

Figure S65. MS analysis of **12** after incubation in H_2O or $H_2{}^{18}O$ on both positive and negative modes. 89

Figure S66. Conversion of **12** to **10** and **11***. 90

Figure S67. Proposed shunt pathway in the absence of RusE. 91

Figure S68. Proposed mechanisms of the RusG-catalyzed oxidations. 91

Figure S69. Representative homologous BGCs of the *rus* cluster identified in other fungi by using cblaster. 92

Supplementary References **93**

Experiment Procedures

1. Media, strains, and growth conditions

Media and solutions used in this study are listed in Table S4. *Penicillium roqueforti* FM164 was cultivated on potato dextrose (PD, Sigma) plates with 1.5% agar at 25°C for sporulation. Rice medium was used for secondary metabolite (SM) production. *Aspergillus nidulans* strains were grown on GMM plates with appropriate supplements as required at 37°C for sporulation (Table S4).

Escherichia coli strains were cultivated either in liquid Lysogeny Broth (LB) medium or on agar plates at 37°C. Recombinant *E. coli* strains were grown in the presence of ampicillin (100 µg/mL). *Saccharomyces cerevisiae* BJ5464-npgA was used for cloning *via* homologous recombination and cultivated at 30°C in YPD medium.[1] Selection was performed using synthetic complete (SC) medium without uracil (SC-Ura).

2. Genomic DNA isolation

For genomic DNA isolation, fungal strains were cultured in PD medium with necessary nutritional additives at 37°C for two or three days. The mycelia were harvested by drying on sterilized filter paper and then placed into 2 mL Eppendorf tubes. Four glass beads and 400 µL of LETS solution (Table S4) were added, and the mixture was homogenized using a Minilys Homogenizer (Bertin Technologies, Montigny-le-Bretonneux, France) for 200 seconds. Additional 300 µL of LETS solution were then introduced. The mixture underwent treatment with 700 µL of phenol: chloroform: isoamyl alcohol (25: 24: 1), followed by vigorous mixing and centrifugation at 13000 rpm for 10 minutes. DNA was precipitated by adding double volume of absolute ethanol to the aqueous phase obtained from the previous step. Following centrifugation at 13000 rpm for 30 min, the resulting DNA pellet was washed with 600 µL of 70% ethanol. After drying, the genomic DNA was dissolved in double-distilled H_2O to achieve a concentration of 100 – 200 ng/µL.

3. Sequence analysis

The genome sequence of *P. roqueforti* FM164 was downloaded from the NCBI database and gene cluster prediction was performed using antiSMASH (http://antismash.secondarymetabolites.org/). The sequences of FogABCD of the flavoglaucin cluster were used as queries to search homologous gene clusters using cblaster,[2] which was integrated in the online server CAGECAT[3] (https://cagecat.bioinformatics.nl/). The targeted gene cluster of interest was further analyzed using 2ndFind (https://biosyn.nih.go.jp/2ndfind/) and BLAST (http://blast.ncbi.nlm.nih.gov) to determine the genes and exon boundaries. The sequence of the reported ruquesalin cluster is on scaffold ProqFM164S06 in GenBank and available under the accession number HG792020.1.

4. PCR amplification, gene cloning, and plasmid construction

Primers were synthesized by Seqlab GmbH (Göttingen, Germany) and used for PCR amplification as given in Table S2. The target sequences were amplified using Phusion® High-Fidelity DNA polymerase from New England Biolabs (NEB). PCR amplification was conducted on a T100TM Thermal Cycler (Bio-Rad), following the thermal profiles recommended by the manufacturer.

Given that the empty plasmid pJN017 contains solely the *gpdA* promoter from *Aspergillus nidulans*, we aimed to construct a heterologous expression plasmid featuring multiple genes with replacement of their promoters by constitutive promoters. We amplified therefore two additional promoters, *i.e. gpdA*$^{\#}$ of *Aspergillus niger* from the plasmid pYTR[4] and *hlyA* of *Aspergillus oryzae*[5] from the genomic DNA of *A. oryzae*. These PCR products were then recombined homologously with the NotI-digested pJN017, generating the basic plasmid pZL132 containing three different constitutive promoters. Using this new plasmid as a template, we constructed all the heterologous expression plasmids by performing homologous recombination in yeast,[1] as detailed in Table S3.

5. Heterologous expression in *A. nidulans*

The strain *A. nidulans* LO8030 was employed as the recipient host for homologous expression. Preparation of protoplasts and PEG-mediated transformation were carried out according to the protocol described previously.[6] The empty vector pZL132, the expression plasmids pZL124, pZL126, pZL161, pZL125, pZL162, pZL111, and pZL164 were introduced into LO8030, resulting in the strains ZL132, ZL124, ZL126, ZL161, ZL125, ZL162, ZL111, and ZL164, respectively. To confirm the corrected transformants, PCR was conducted using primers listed in Table S2. The transformants were then cultivated in rice medium for the production of secondary metabolites.

6. LC-HRMS analysis for secondary metabolites

HPLC, LC-MS, and NMR analyses of secondary metabolites were conducted following previously established protocols.[7] ACN and H_2O, each with 0.1% (v/v) HCOOH, were used as solvents for HPLC and LC-MS analyses at a flow rate of 0.3 mL/min. The linear gradient was from 5% to 100% ACN in H_2O over 30 min. For LC-MS analysis, an Agilent 1260 HPLC system was equipped with a Bruker microTOF QIII mass spectrometer and a VDSpher PUR 100 C18-M-SE column (150 × 2 mm, 3 μm) (VDS optilab Chromatographie Technik).

7. Cultivation of *A. nidulans* ZL111 with 3-amino-1-propanol

To test the acceptance of 3-amino-1-propanol by the *rus* cluster in ZL111, the spores of ZL111 and the control strain ZL132 were cultivated in rice medium. After cultivation for 5 days, 100 μL of 3-amino-1-propanol (Sigma-Aldrich, CAS-No: 156-87-6) were

added to the cultures. After further cultivation at 25°C for 3 days, another 100 μL of 3-amino-1-propanol were added to give a final concentration of about 150 mM. The 14-day old cultures were extracted with EtOAc twice and dissolved in methanol for LC-MS analysis.

8. Large-scale fermentation, extraction, and isolation of secondary metabolites

To isolate the secondary metabolites, the *A. nidulans* transformants were cultivated in 10 × 1 L-Fernbach flasks each containing 100 g jasmine rice (Royal Tiger) and 100 mL H_2O supplemented with 750 mg/L uracil, 750 mg/L uridine, and 0.5 mg/L pyridoxin at 25°C for 14 days. The fungal cultures were extracted three times with EtOAc and concentrated under reduced pressure to obtain crude extracts. The obtained crude extracts were separated by silica gel column chromatography using a gradient of petroleum ether (40 – 60 °C) / EtOAc (20:1 to 0:1) as elution solvents. After LC-MS analysis of the obtained fractions, a semi-preparative HPLC on an Agilent HPLC 1200 equipped with an Agilent Eclipse XDB-C18 column (9.4 × 250 mm, 5 μm, VDS Optilab Chromatographie Technik GmbH, Germany, Bremen) was used to obtain the compounds with the conditions given below.

Stachysalicyloid B (**1**, 10.7 mg, t_R = 17.8 min), stachysalicyloid D (**2**, 8.5 mg, t_R = 18.0 min), and roquesalin A (**3**, 3.2 mg, t_R = 29.0 min) were obtained from the fermentation broth of *A. nidulans* ZL124 on a semi-preparative HPLC at a flow rate of 2.0 mL/min by isocratic elution with ACN: H_2O at 85:15, 95:5, and 55:45, respectively.

Roquesalin B (**4**, 5.3 mg, t_R = 14.0 min), roquesalin C (**5**, 3.0 mg, t_R = 29.0 min), roquesalin D (**6**, 2.1 mg, t_R = 32.5 min), and roquesalin E (**7**, 2.6 mg, t_R = 43.5 min), were obtained from fractions of *A. nidulans* ZL111 on a semi-preparative HPLC at a flow rate of 2.0 mL/min by isocratic elution with ACN:H_2O at 60:40, 60:40, 65:35, and 65:35, respectively.

Roquesalin F (**8**, 1.5 mg, t_R = 16.0 min) and roquesalin G (**9**, 8.3 mg, t_R = 35.0 min) were obtained from fractions of *A. nidulans* ZL111 supplemented with 75 mM of 3-amino-1-propanol on a semi-preparative HPLC with ACN: H_2O at 60:40 and 62:38, respectively.

Roquesalin H (**10**, 3.6 mg, t_R = 29 min) and roquesalin I (**12**, 4.8 mg, t_R = 25.0 min) were obtained from fractions of *A. nidulans* ZL162 on a semi-preparative HPLC with ACN/H_2O at 60:40, and 48:52, respectively.

Roquesalin J (**14**, 3.4 mg, t_R = 28.5 min) was obtained from a fraction of *A. nidulans* ZL161 on a semi-preparative HPLC with ACN/H_2O at 55:45.

9. NMR analysis

NMR spectra were recorded on a JEOL ECA-500 MHz spectrometer (JEOL, Tokyo, Japan). The spectra were processed with MestReNova 6.1.0 (Metrelab, Santiago de Compostela, Spain). Chemical shifts are referenced to those of the solvent signals.

To determine the relative configuration of **10** by calculation, conformational searches were first done using the iMTD-GC method embedded in Crest program.[8] Density functional theory calculations were performed with the Gaussian 16 package (https://gaussian.com/gaussian16/). The obtained conformers with population over 1% were optimized at B3LYP-D3BJ/6-31G(d) in gas phase and the conformers within an energy window of 3 kcal/mol were kept. These conformers were refined by re-optimizations at B3LYP-D3BJ/6-311G (d, p) with IEFPCM solvent model in chloroform. and frequency analysis of all optimized conformations were also performed at the same level of theory to exclude the imaginary frequencies. NMR shielding tensors were calculated with the GIAO method at mPW1PW91/6-311G (d, p) level with IEFPCM solvent model in chloroform. The shielding constants were converted into chemical shifts by referencing to TMS at 0 ppm (δcal = σTMS – σcal), where the σTMS (the shielding constant of TMS) was calculated at the same level. For each candidate, the parameters a and b of the linear regression δcal = aδexp + b; the correlation coefficient, R^2; the mean absolute error (MAE) defined as Σn |δcal – δexp|/n; the corrected mean absolute error, CMAE, defined as Σn |δcorr–δexp|/n, where δcorr = (δcal – b)/a, were calculated. DP4+ probability analysis was performed using the calculated NMR shielding tensors.[9,10]

10. Structural elucidation

The structures of **1** and **2** were elucidated by comparison of their NMR data (Table S6 and Figures S4–S10) with those of stachysalicyloids B and D, respectively.[11] The structures of the new identified compounds were elucidated by comprehensive interpretation of their UV, MS, CD and NMR data (Tables S6−S13 and Figures S11−S64).

The molecular weight of roquesalin A (**3**) at 287.1623, 16 daltons larger than that of **1**, suggests one additional oxygen atom in its structure. Its ^{1}H and ^{13}C NMR data are similar to those of stachysalicyloid B (**1**) and differ merely by two doublets at δ_H 6.65 (H-4, d, 8.5 Hz) and 6.64 ppm (H-5, d, 8.5 Hz), instead of two doublets and one triplet in **1**. A significant downfield shift of C-6 to δ_c 147.0 ppm, HMBC correlations from H-8 to C-6, as well as from 6-OH and H-8 to C-7 prove a hydroxyl group at C-6 in **3**.

Roquesalin B (**4**) showed a $[M+H]^+$ ion at *m/z* 320.1867 with a deduced molecular formula of $C_{18}H_{26}NO_4$. Interpretation of its ^{1}H and ^{13}C spectral data revealed the same substitution pattern at the aromatic ring as those of **1** and **2**. Significant changes were observed for the signals of the alkyl chain and those of two additional coupling methylenes at δ_H 3.70 (H-2') and at δ_H 3.51 (H-3') ppm, likely attached to two hetero atoms. ^{1}H-^{1}H COSY correlations of the protons at the alkyl chain, *e.g.* from the olefinic H-9 at 5.51 ppm to H-10 at 4.85 ppm and H-10 to the methylene protons indicate the presence of a double bond between C-8 and C-9 as well as a hydroxyl group at C-10. This conclusion was confirmed by the HMBC correlations from H-9 to C-7 and C-8. The ^{1}H-^{1}H COSY correlation between H-2' and H-3', as well as the HMBC correlations from H-2' to C-1, C-8 and C-3' revealed the presence of an isoindole skeleton and the

attachment of an ethanol to the nitrogen atom. NOESY correlation of H-6 to H-10 assigned the *E*-configuration of the double bond between C-8 and C-9. The *S*-configuration at C-10 was determined by ECD calculation. Compound **4** was named roquesalin B.

Roquesalin C (**5**) was found to have the molecular formula $C_{19}H_{27}NO_4$. Careful comparison of the ^{1}H and ^{13}C NMR data of **5** and **4** showed high similarity, with the exception for additional signals at δ_H 3.19 ppm (H-17) and δ_c 55.3 ppm (C-17) in those of **5**, indicating the replacement of a hydroxyl group in **4** by a methoxyl group in **5**. HMBC correlation from H-17 to C-10 confirmed **5** to be a methylated product of **4**. Therefore, the absolute configuration of **5** was also assigned as 10*S* based on the ECD calculation.

Roquesalin D (**6**) has a $[M+H]^+$ ion at *m/z* 261.1490 and a deduced molecular formula of $C_{16}H_{21}O_3$. Comparison with molecular formula of **2** suggests one additional oxygen atom and one degree of unsaturation in **6**. H-8 appears as a triplet at 5.49 ppm in the ^{1}H-NMR spectrum. Together with the signal of C-8 at 82.4 ppm suggests the linkage with an oxygen atom. HMBC correlations from H-8 to C-1 confirmed the ester bond between C-1 and C-8 to form a γ-lactone ring in **6**. The ^{1}H-^{1}H COSY correlations from H-8 *via* H-9, two olefinic protons H-10 (5.33 ppm, dt, 15.0 and 7.5 Hz) and H-11 (5.57 ppm, dt, 15.0 and 7.5 Hz), four methylenes (H-12 − H-15), to the methyl protons H-16, suggest the double bond between C-10 and C-11 with a *trans* configuration at the alkyl chain.

Roquesalin E (**7**) with a molecular weight of 259.3250 and deduced molecular formula of $C_{16}H_{19}O_3$ has one more degree of unsaturation than **6**. Two ^{13}C signals at δ_c 143.5 and δ_c 110.0 ppm indicates introduction of one additional C-C double bond. ^{1}H-^{1}H COSY correlations from H-9 to H-10 and H-10 to H-11 prove its location between C-8 and C-9.

Roquesalin G (**9**) with a $[M + H]^+$ of 348.2179 and a deduced molecular formula of $C_{20}H_{30}NO_4$, suggesting one more methylene group than **5** and corresponding to the expected incorporation of 3-amino-1-propanol instead of ethanolamine. HMBC correlations from H-4' to C-2' and C-3', as well as H-3' to C-2' and C-4' confirmed this conclusion. In its NMR spectra, signals for a methoxyl group were detected at δ_H 3.22 and δ_c 55.3 ppm. HMBC correlation from H_3-17 to C-10 confirmed the methylation of the OH-10. Roquesalin F (**8**) has a $[M + H]^+$ of 334.2023, 14 Daltons smaller than that of **9** and 14 Daltons larger than that of **4**. Comparison of its ^{1}H NMR spectrum with that of **9** proved the replacement of the methoxyl group at C-10 by a hydroxyl group. The absolute configurations of **8** and **9** were also assigned as 10*S* based on the ECD calculation, and with the comparison of compound **4**.

Inspection of the NMR data of roquesalin H (**10**) indicates the same substituted benzene ring as in most metabolites identified in this study. The presence of signals at δ_H 5.08 and δ_C 71.8 ppm indicates that the original CH_2O at the benzene ring is not oxidized. ^{1}H-^{1}H COSY provided evidence for a coupling system from CH_2-9 to CH_3-16.

Together with the resonances at δ_H 3.06 and δ_C 50.2 ppm for a methoxyl group, the signal at δ_C 110.5 ppm for C-8 indicates the ketal character at this position. HMBC correlations from H-1 and CH_3-17 to C-8 confirmed this conclusion and the formation of a tetrahydro benzofuran ring. In addition, signals for olefinic protons in **1** – **9** disappeared. Instead, appearance of signals at δ_H 3.29 (H-9, d, 2.0 Hz) and 3.30 ppm indicates the presence of an epoxide ring. ^1H and ^{13}C NMR spectra of roquesalin I (**12**) are very similar to those of **10**, with the exception for the disappearance of the methyl group at OH-8 of **12**. The coupling constant of 2.0 Hz for H-9 and H-10 suggests a *trans* configuration of the epoxy group, which was also confirmed by NOESY correlation between H-9 and H-11. The relative configuration of **12** was determined *via* NMR chemical shift calculation coupled with DP4+ analysis (Tables S12 and S13, Figure S61) and its absolute configuration was then assigned as 8*R*,9*R*,10*S* based on the ECD calculation. Accordingly, the methylated derivative **10** has also the same absolute configuration.

Roquesalin J (**14**) was found to have a $[M + H]^+$ of 304.1921 and a deduced molecular formula $C_{18}H_{26}NO_3$, suggesting the lack of one hydroxyl group than **4** ($C_{18}H_{26}NO_4$). The ^1H NMR spectrum of **14** also shows high similarity with that of **4**, with the exception for the coupling system of the alkyl chain, beginning from H-8 in **14** instead of H-9 in **4**. HMBC correlations from H-8 to C-7, C-9, and C-10, from H-2' to C-8 as well as H-9 to C-7 confirmed the double bond between C-9 and C-10 in **14**. The coupling constant of 14.5 Hz for H-9 and H-10 proves its *E*-form. The specific rotation of **14** was determined to be zero, indicating the presence of an enantiomeric pair.

11. Precursor supply for *A. nidulans* ZL164 and ZL169

For biotransformation, the transformants ZL164 and ZL169 as well as the control strain ZL132 were cultivated in rice medium. Stachysalicyloid B was dissolved in DMSO to give 50 mM stock solution. After cultivation for 5 days, 50 µL stock solution were added to the cultures, as well as to blank rice medium, to give a final concentration of about 160 µM. After cultivation at 25°C for additional 3 and 7 days, the secondary metabolites were extracted with EtOAc twice and dissolved in methanol for LC-MS analysis.

12. Proof of the conversion of 12 to 10 and 11*

To provide evidence for the conversion of **12** to the methylated products **10** and **11***, compound **12** isolated from the fungal culture of ZL162 was dissolved in DMSO to give a 20 mM stock solution. Incubation of 1 µL stock solution with 100 µL CD_3OH and $H_2{}^{18}O$ were performed at room temperature for 16 h. The sample with CD_3OH was subsequently analyzed by LC-MS on positive and negative modes. While the sample with $H_2{}^{18}O$ was analyzed directly by MS on positive and negative modes.

13. Measurement of optical rotations

The optical rotation was measured with the polarimeter Jasco DIP-370 at 20°C using the D-line of the sodium lamp at λ = 589.3 nm. Prior to the measurement, the

polarimeter was calibrated with MeOH as solvent.

14. The experimental and calculated electronic circular dichroism (ECD) spectroscopic analysis

The experimental ECD spectra were taken on a J-1500 CD spectrometer (Jasco Deutschland GmbH, Pfungstadt, Germany). The samples were dissolved in MeOH and measured in the range of 200 – 400 nm by using a 1 mm path length quartz cuvette (Hellma Analytics, Müllheim, Germany).

For ECD calculation, conformers of a given compound were generated by the Confab program[12] embedded in the Openbabel 3.1.1 software. The conformers were further optimized with xtb at GFN2 level[13] and the conformers with population over 1% were subjected to geometry optimization using the Gaussian 16 package (https://gaussian.com/gaussian16/) at B3LYP/6-31G(d) level and frequency analysis, then proceeded to calculation of excitation energies, oscillator strength, and rotatory strength at B3LYP/TZVP level in the polarizable continuum model (PCM, methanol). The calculated ECD spectra were Boltzmann weighted and generated using SpecDis 1.71 software.[9] The experimental and calculated ECD spectra are given in Figures S62 – S64.

15. Physiochemical properties of the compounds described in this study

Stachysalicyloid B (**1**): Colorless oil; HRMS(ESI) *m/z*: 271.1674 $[M + Na]^+$ calcd. for $C_{16}H_{24}O_2Na$; found 271.1674. NMR data are listed in Table S6 and NMR spectra as Figures S4 – S8.

Stachysalicyloid D (**2**): Pale yellow oil; HRMS(ESI) *m/z*: 247.1698 $[M + H]^+$ calcd. for $C_{16}H_{23}O_2$; found 247.1698. NMR data are listed in Table S6 and NMR spectra as Figure. S9 – S10.

Roquesalin A (**3**): Pale yellow oil; $[\alpha]_D^{20}$ = +80 (c 0.1, MeOH); HRMS(ESI) *m/z*: 287.1623 $[M + Na]^+$ calcd. for $C_{16}H_{24}O_3Na$; found 287.1623. NMR data are listed in Table S6 and NMR spectra as Figures S11 – S15.

Roquesalin B (**4**): Pale yellow oil; $[\alpha]_D^{20}$ = +55 (c 0.2, MeOH); HRMS(ESI) *m/z*: 320.1862 $[M + H]^+$ calcd. for $C_{18}H_{26}NO_4$; found 320.1867. NMR data are listed in Table S6 and NMR spectra as Figures S16 – S21.

Roquesalin C (**5**): Pale yellow oil; $[\alpha]_D^{20}$ = +60 (c 0.1, MeOH); HRMS(ESI) *m/z*: 334.2018 $[M + H]^+$ calcd. for $C_{19}H_{28}NO_4$; found 334.2023. NMR data are listed in Table S7 and NMR spectra as Figures S22 – S26.

Roquesalin D (**6**): Pale solid; $[\alpha]_D^{20}$ = +70 (c 0.2, MeOH); HRMS(ESI) *m/z*: 261.1490 $[M + H]^+$ calcd. for $C_{16}H_{21}O_3$; found 261.1490. NMR data are listed in Table S7 and NMR spectra as Figures S27 – S31.

Roquesalin E (**7**): Pale solid; $[\alpha]_D^{20}$ = +70 (c 0.1, MeOH); HRMS(ESI) *m/z*: 259.3248 $[M + H]^+$ calcd. for $C_{16}H_{19}O_3$; found 259.3250. NMR data are listed in Table S7 and

NMR spectra as Figures S32 – S37.

Roquesalin F (**8**): Pale yellow oil; $[\alpha]_D^{20}$ = +60 (c 0.1, MeOH); HRMS(ESI) *m/z*: 334.2018 $[M + H]^+$ calcd. for $C_{19}H_{28}NO_4$; found 334.2023. NMR data are listed in Table S7 and ^1H NMR spectrum as Figure S38.

Roquesalin G (**9**): Pale yellow oil; $[\alpha]_D^{20}$ = +50 (c 0.1, MeOH); HRMS(ESI) *m/z*: 348.2175 $[M + H]^+$ calcd. for $C_{20}H_{30}NO_4$; found 348.2179. NMR data are listed in Table S8 and NMR spectra as Figures S39 – S42.

Roquesalin H (**10**): Pale yellow oil; $[\alpha]_D^{20}$ = +36.7 (c 0.3, MeOH); HRMS(ESI) *m/z*: 315.1573 $[M + Na]^+$ calcd. for $C_{17}H_{24}O_4Na$; found 315.1575. NMR data are listed in Table S8 and NMR spectra as Figures S43 – S47.

Roquesalin I (**12**): Pale yellow oil; $[\alpha]_D^{20}$ = +30 (c 0.4, MeOH); HRMS(ESI) *m/z*: 301.1416 $[M + Na]^+$ calcd. for $C_{16}H_{22}O_4Na$; found 301.1416. NMR data are listed in Table S9 and NMR spectra as Figures S48 – S53.

Roquesalin J (**14**): Pale yellow oil; $[\alpha]_D^{20}$ = 0 (c 0.3, MeOH); HRMS(ESI) *m/z*: 304.1913 $[M + H]^+$ calcd. for $C_{18}H_{26}NO_3$; found 304.1921. NMR data are listed in Table S9 and NMR spectra as Figures S54 – S58.

Supplementary Tables

Table S1. Sequence identities of proteins encoded by the *rus* cluster from *Penicillium roqueforti* FM164 with those of known clusters.

**Protein**	**Length in aa**	**Homologous proteins**	**Identity/%** (cover/%)	**Putative function**
RusA (XP_038926972.1)*	2355	AnuA (CDM34450.1, *Penicillium roqueforti*)	43.2 (90)	Highly reducing polyketide synthase
		FogA (XP_040639024.1, *Aspergillus ruber*)	51.0 (99)	
		StrA (BDX35601.1, *Stachybotrys* sp.)	51.5 (99)	
RusB (CDM37418.1)	273	AnuF (CDM34455.1, *P. roqueforti*)	50.6 (98)	Short-chain dehydrogenase/reductase
		FogB (XP_040639025.1, *A. ruber*)	54.6 (99)	
		StrB (BDX35602.1, *S.* sp.)	53.9 (99)	
RusC (CDM37417.1)	196	AnuC (CDM34452.1, *P. roqueforti*)	60.1 (90)	Cupin domain-containing protein
		FogC (XP_040639025, *A. ruber*)	58.6 (99)	
		StrC (BDX35603.1, *S.* sp.)	63.7 (96)	
RusD (CDM37412.1)	284	AnuB (CDM34451.1, *P. roqueforti*)	56.0 (89)	Short-chain dehydrogenase/reductase
		FogD (XP_040639026.1, *A. ruber*)	58.4 (99)	
		StrD (BDX35604.1, *S.* sp.)	58.3 (98)	
RusE (CDM37416.1)	489	AnuG (CDM34456.1, *P. roqueforti*)	30.6 (92)	Flavin-containing monooxygenases
		FogF (XP_040639028.1, *A. ruber*)	41.0 (93)	
RusF (CDM37407.1)	499	A0A1Y1C7S2.1 (*Aspergillus stellatus*)	31.3 (88)	Cytochrome P450
RusG (XP_038926975.1)*	568	Q0CSA3.1 (*Aspergillus terreus*)	38.6 (96)	Flavin-containing monooxygenases
RusH (CDM37419.1)	373	S0DPL8.1 (*Fusarium fujikuroi*)	41.9 (8)	Transcription factor

*Due to the truncated annotation in the genome of *P. roqueforti* FM164, we referenced the homologous protein sequences of RusA and RusG from *P. roqueforti* LCP96 04111. The respective proteins share identical sequences with each other on the amino acid level.

Table S2. Primers used in this study.

**Primers**	**Sequence 5'-3'**
ZL_pZL80_FOR	AGAATCCTATAAATTGGGAAAACTAGAGACCCCGGTCTGAGTGAAGTTCCGGTCGGAG
ZL_pZL80_REV	TCATAGTAAAGTGATTCGCGTCATTAAAGCGGCCGCGGTGTTGTGGTGTGAAGGGTG
ZL_pZL132_FOR	AATCACCCTTCACACCACAACACCGCGGCCGCTTTATGTTTAGATGTGTCTATGTGGCG
ZL_pZL132_REV	CCTTTTCGCCCTTTGTCATAGTAAAGTGATTCGCGTCATACTCCGGTGAATTGATTTGG
ZL_pZL99-1_FOR	GGTCTCACATCAATCACCCTTCACACCACAACACCATGAAGTCCGTTTTAATTACAGGG
ZL_pZL93-3_FOR_new	ATGAGTCGTGGTCATTAGAGT
ZL_pZL93-2_REV	TGATGCCTCAATTCGCCTG
ZL_pZL93-2_FOR	AGCAACGTAGGCAGCTTC
ZL_pZL93-1_REV	ATCCAAGCTGTCACTGCTG
ZL_pZL111-2_REV	GACTAACCATTACCCCGCCACATAGACACATCTAAACAATGGCTAAAATGGCTTCAACT
ZL_pZL92-1_FOR	TGACTAACAGCTACCCCGCTTGAGCAGACATCACCATGGGTTCTATCGACGTTTCTTC
ZL_pZL92-1_REV	TCTTGCCAGACAGCGTGTAG
ZL_pZL92-2_FOR	ACCACACTCAATGATCTGTTCAG
ZL_pZL133-2_REV	TCCCAGCCTCAACACCATATTTTAATCCCATGTGGGCGCCCGTTCATTGGCTGAGTGAG
ZL_pZL134_REV	CCCCGCCACATAGACACATCTAAACATAAAGCGGCCGCATGAGTCGTGGTCATTAGAGT
ZL_pZL134-1_R	CCGCCACATAGACACATCTAAACATAAAGCGGCCGCGGTGATGGGAATGTTAAAAGGAG
ZL_pZL92-2_REV	GCCTCAACACCATATTTTAATCCCATGTGGGCGCCTAGCGCTACGGATATAGCTCAAG
ZL_pZL120_FOR	AGTTACTCTAATGACCACGACTCATGCGGCCGCTTTAACTGATGAACGTTGTCCTTGAT
ZL_pZL92-2_REV	GCCTCAACACCATATTTTAATCCCATGTGGGCGCCTAGCGCTACGGATATAGCTCAAG
ZL_pZL161_FOR	AAGTTACTCTAATGACCACGACTCATGCGGCCGCTTTATCTGACTGTCTGACACGAAGC
ZL_pZL161_REV	TTCATTCGGTAAGCCCTACTCT
ZL_pZL162_REV	TAATTATCAAGGACAACGTTCATCAGTTAAAGCGGCCGCGTTGATCGCCAAGCTTCAAC
ZL_pZL143_FOR	CAATCACCCTTCACACCACAACACCGCGGCCGCTTTAACTGATGAACGTTGTCCTTGAT
ZL_pZL164_FOR	GCTTGACTAACAGCTACCCCGCTTGAGCAGACATCACCATGCATATGCAAGGAACCATG
ZL_pZL164_REV	CAGCCTCAACACCATATTTTAATCCCATGTGGGCGCCAGTGCATGATTTGGATGTACTG
ZL_pZL169-FOR	CTTGACTAACAGCTACCCCGCTTGAGCAGACATCACCATGGGTTCTACATCGAACAACT
ZL_pZL169_REV	AATATCCCAGCCTCAACACCATATTTTAATCCCATGTGAGTAGCATCAAATCAGCTCCT
GT_wa_DS	TAGATGCAGTCTCTCAAGAAC
GT_wa_US	AGCGGACTCGAAGTATGTT

Table S3. Plasmids used and constructed in this study.

Plasmid	Insert	Primer 1	Primer 2	PCR Template	Vector	Source/Ref.
pJN017	-	-	-	-	-	[6]
pYTR	-	-	-	-	-	[4]
pZL80	*hlyA*(p) from *A. oryzae*	ZL_pZL80_FOR	ZL_pZL80_REV	gDNA of *A. oryzae* RIB40	pJN017 digested with NotI	This study
pZL132	*gpdA*(p) from pYTR	ZL_pZL132_FOR	ZL_pZL132_REV	Plasmid pYTR	pZL80 digested with NotI	
pZL133	1st fragment of *rusA*	ZL_pZL92-1_FOR	ZL_pZL92-1_REV	gDNA of *P. roqueforti* FM164	pZL132 digested with SfoI	
	2nd fragment of *rusA*	ZL_pZL92-2_FOR	ZL_pZL133-2_REV			
pZL134	*rusD*	ZL_pZL99-1_FOR	ZL_pZL134_REV		pZL133 digested with NotI	
pZL124	*rusB-rusC-rusH*	ZL_pZL120_FOR	ZL_pZL111-2_REV		pZL134 digested with NotI	
pZL92-1	1st fragment of *rusA+rusF*	ZL_pZL92-1_FOR	ZL_pZL92-1_REV		pZL132 digested with SfoI	
	2nd fragment of *rusA+rusF*	ZL_pZL92-2_FOR	ZL_pZL92-2_REV			
pZL111	1st fragment of *rusD-rusG-rusE-rusC-rusB-rusH*	ZL_pZL99-1_FOR	ZL_pZL93-3_FOR_new		pZL92-1 digested with NotI	
	2nd fragment of *rusD-rusG-rusE-rusC-rusB-rusH*	ZL_pZL93-2_REV	ZL_pZL93-2_FOR			
	3rd fragment of *rusD-rusG-rusE-rusC-rusB-rusH*	ZL_pZL93-1_REV	ZL_pZL111-2_REV			
pZL125	1st fragment of *rusD-rusG-rusE-rusC-rusB-rusH*	ZL_pZL99-1_FOR	ZL_pZL93-3_FOR_new		pZL133 digested with NotI	
	2nd fragment of *rusD-rusG-rusE-rusC-rusB-rusH*	ZL_pZL93-2_REV	ZL_pZL93-2_FOR			
	3rd fragment of *rusD-rusG-rusE-rusC-rusB-rusH*	ZL_pZL93-1_REV	ZL_pZL111-2_REV			

Table S3 continued

pZL153-1	*rusD*	ZL_pZL99-1_FOR	ZL_pZL134_REV	gDNA of *P. roqueforti* FM164	pZL92-1 digested with NotI	This study
pZL154	*rusB-rusC-rusH*	ZL_pZL120_FOR	ZL_pZL111-2_REV		pZL153-1 digested with NotI	
pZL126	*rusE*	ZL_pZL161_FOR	ZL_pZL161_REV		pZL124 digested with NotI	
pZL161	*rusE*	ZL_pZL161_FOR	ZL_pZL161_REV		pZL154 digested with NotI	
pZL162	*rusG*	ZL_pZL93-2_REV	ZL_pZL162_REV		pZL154 digested with NotI	
pZL164	*rusE*	ZL_pZL164_FOR	ZL_pZL164_REV		pZL132 digested with SfoI	
pZL169	*rusG*	ZL_pZL169_FOR	ZL_pZL169_REV		pZL132 digested with SfoI	

Table S4. Media and solutions used in this study.

Medium/Solution	Ingredients
PD	24 g/L potato dextrose broth (Sigma)
GMM	1.0% glucose, 50 mL/L salt solution, 1 mL/L trace element solution, 1.6% agar
LB	1% NaCl, 1% tryptone, 0.5% yeast extract
YPD	1% yeast extract, 2% peptone, 2% glucose
SC-Ura	6.7 g/L yeast nitrogen base with ammonium sulfate, 650 mg/L CSM-His-Leu-Ura, 20 mg/L histidine, 60 mg/L leucine
SMM	1.0% glucose, 50 mL/L salt solution, 1 mL/L trace element solution, 1.2 M sorbitol
Salt solution	12% $NaNO_3$, 1.04% KCl, 1.04% $MgSO_4 \cdot 7H_2O$, 3.04% KH_2PO_4
Trace elements solution	2.2% $ZnSO_4 \cdot 7H_2O$, 1.1% H_3BO_3, 0.5% $MnCl_2 \cdot 4H_2O$, 0.16% $FeSO_4 \cdot 7H_2O$, 0.16% $CoCl_2 \cdot 5H_2O$, 0.16% $CuSO_4 \cdot 5H_2O$, 0.11% $(NH_4)_6Mo_7O_{24} \cdot 4H_2O$, 5% Na_4EDTA
LETS solution	20 mM EDTA pH 8.0, 0.5% (*w/v*) SDS, 0.1 M LiCl, 10 mM Tris-HCl, pH 8.0
Osmotic buffer	1.2 M $MgSO_4$ in 10 mM sodium phosphate, pH 5.8
Trapping buffer	0.6 M sorbitol in 0.1 M Tris-HCl, pH 7.0
STC buffer	1.2 M sorbitol, 10 mM $CaCl_2$, 10 mM Tris-HCl, pH 7.5
PEG solution	60% PEG 4000, 50 mM $CaCl_2$, and 50 mM Tris-HCl, pH 7.5

Table S5. Strains used in this study.

Strain	Genotype	Created with plasmid	Source/Ref.
E. coli DH5α	F⁻*endA1 glnV44 thi-1 recA1 relA1 gyrA96 deoR nupG purB20* φ80d*lacZ*ΔM15 Δ(*lacZYA-argF*)U169, hsdR17($r_K^- m_K^+$), λ⁻	-	14
S. cerevisiae BJ5464-npgA	*MATα ura3-52 leu2-Δ1 trp1 his3-Δ200 pep4::HIS3 prb1-Δ1.6R can1 GAL npgA*	-	15
P. roqueforti FM164	WT	-	2
A. nidulans			
LO8030	*pyroA4*, *riboB2*, *AfpyrG89*, *nkuA*::*argB*, sterigmatocystin cluster (ANIA_07804 – ANIA_07825)Δ, emericellamide cluster (ANIA_02545 – ANIA_02549)Δ, asperfuranone cluster (ANIA_01039 – ANIA_01029)Δ, monodictyphenone cluster (ANIA_10023 – ANIA_10021)Δ, terrequinone cluster (ANIA_08512 – ANIA_08520)Δ, austinol cluster part 1 (ANIA_08379 – ANIA_08384)Δ, austinol cluster part 2 (ANIA_09246 – ANIA_09259)Δ, F9775 cluster (ANIA_07906 – ANIA_07915)Δ, asperthecin cluster (ANIA_06000 – ANIA_06002) Δ	-	16
ZL132	Δ*wA*-PKS::*gpdA*(p)-*Afribo-hlyA*(p)-*gpdA*#(p) in LO8030	pZL132	This study
ZL124	Δ*wA*-PKS::*gpdA*(p):*rusA-Afribo-hlyA*(p):*rusD-rusBC-gpdA*(p):*rusH* in LO8030	pZL124	This study
ZL111	Δ*wA*-PKS::*gpdA*(p):*rusA-rusF-Afribo-hlyA*(p):*rusD-rusBCEG-gpdA*#(p):*rusH* in LO8030	pZL111	This study
ZL126	Δ*wA*-PKS::*gpdA*(p):*rusA-Afribo-hlyA*(p):*rusD-rusBCE-gpdA*#(p):*rusH* in LO8030	pZL126	This study
ZL125	Δ*wA*-PKS::*gpdA*(p):*rusA-Afribo-hlyA*(p):*rusD-rusBCEG-gpdA*#(p):*rusH* in LO8030	pZL125	This study
ZL161	Δ*wA*-PKS::*gpdA*(p):*rusA-rusF-Afribo-hlyA*(p):*rusD-rusBCE-gpdA*#(p):*rusH* in LO8030	pZL161	This study
ZL162	Δ*wA*-PKS::*gpdA*(p):*rusA-rusF-Afribo-hlyA*(p):*rusD-rusBCG-gpdA*#(p):*rusH* in LO8030	pZL162	This study
ZL164	Δ*wA*-PKS::*gpdA*(p):*rusE-Afribo-hlyA*(p)-*gpdA*#(p) in LO8030	pZL164	This study
ZL169	Δ*wA*-PKS::*gpdA*(p):*rusG-Afribo-hlyA*(p)-*gpdA*#(p) in LO8030	pZL169	This study

WT: Wild type

Table S6. ^{1}H (500 MHz) and ^{13}C NMR (125 MHz) spectroscopic data of stachysalicyloid B (**1**), stachysalicyloid D (**2**), roquesalin A (**3**) in $CDCl_3$, and roquesalin B (**4**) in DMSO-d_6 with key HMBC and ^{1}H-^{1}H COSY correlations.

Compound	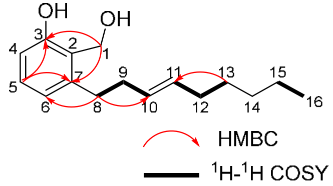		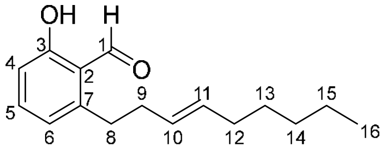		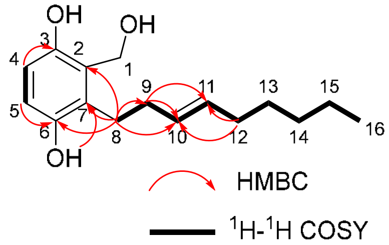		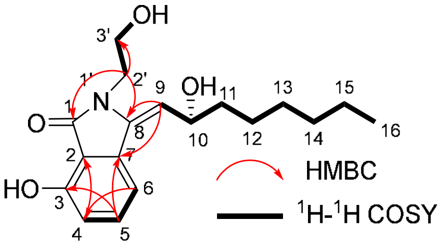	
	**stachysalicyloid B**		**stachysalicyloid D**		**roquesalin A**		**roquesalin B**	
Position	δ_H, multi., J in Hz	δ_C	δ_H, multi., J in Hz	δ_C	δ_H, multi., J in Hz	δ_C	δ_H, multi., J in Hz	δ_C
1	4.88, s	60.0	10.29, s	195.4	4.89, br s	60.2	-	165.2
1-OH	n. d.	-	-	-	n. d.	-	-	-
2	-	123.0	-	118.0	-	124.4	-	114.7
3	-	156.6	-	163.4	-	150.5	-	154.5
3-OH	7.78, brs	-	11.95, br s	-	6.98, br s	-	n. d.	-
4	6.73, d, 8.0	114.5	6.84, d, 8.5	116.2	6.65, d, 8.5	115.9	6.91, d, 8.0	116.5
5	7.09, t, 8.0	128.9	7.41, dd, 8.5, 7.5	137.5	6.64, d, 8.5	114.6	7.45, t, 8.0	133.6
6	6.72, d, 8.0	121.7	6.74, d, 7.5	121.4	-	147.0	7.30, d, 8.0	115.1
6-OH	-	-	-	-	4.49, br s	-	-	-
7	-	140.6	-	146.8	-	126.7	-	136.3
8	3.04, t, 8.0	33.4	2.98, t, 8.0	35.9	2.68, t, 8.0	26.5	-	134.2
9	2.18, m	34.7	2.34, m	32.6	2.16, m	33.3	5.51, d, 8.5	116.1
10	5.42, dt, 15.0, 7.0	128.9	5.42, dt, 15.0, 7.0	127.9	5.45[a], dt, 15.0, 7.0	129.0	4.85, dt, 9.0, 7.0	65.6
10-OH	-	-	-	-	-	-	5.00, br s	-
11	5.42, dt, 15.0, 7.0	131.7	5.42, dt, 15.0, 7.0	132.7	5.45[a], dt, 15.0, 7.0	132.1	1.62, m; 1.52, m	38.0
12	1.98, m	32.6	1.97, m	32.0	1.97, m	32.6	1.35, m	24.8
13	1.31, m	29.3	1.32, m	29.2	1.33, m	29.2.	1.29, m	28.7
14	1.23, m	31.5	1.26, m	31.5	1.26, m	31.5	1.24, m	31.2
15	1.29, m	22.7	1.29, m	22.7	1.29, m	22.6	1.24, m	22.0
16	0.89, t, 7.0	14.2	0.89, t, 7.0	14.2	0.87, t, 7.0	14.2	0.83, t, 6.5	13.9
2'	-	-	-	-	-	-	3.70, td, 6.5, 2.0	40.7
3'	-	-	-	-	-	-	3.51, t, 6.5	58.1
3'-OH	-	-	-	-	-	-	4.79, br s	-

n. d.: Signals not detected. [a] Signals overlapping with each other.

The ^{1}H NMR data of **1** and **2** correspond well to those of stachysalicyloids B and D, respectively.[11]

Table S7. ^{1}H (500 MHz) and ^{13}C NMR (125 MHz) spectroscopic data of roquesalin C (**5**) and in DMSO-d_6, and roquesalin D (**6**), roquesalin E (**7**), roquesalin F (**8**) in $CDCl_3$ with key HMBC and ^{1}H-^{1}H COSY correlations.

Compound	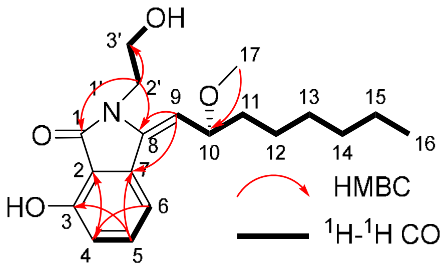		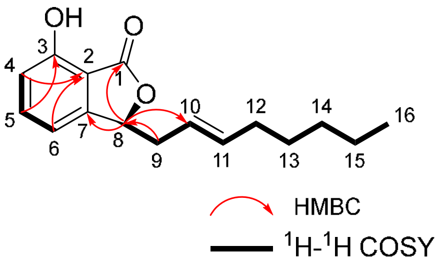		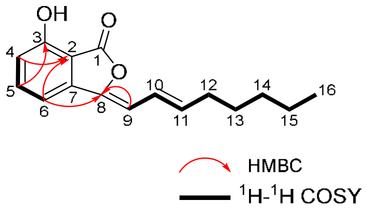		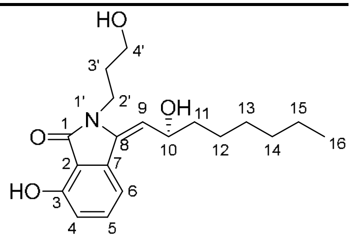
	**roquesalin C**		**roquesalin D**		**roquesalin E**		**roquesalin F**
Position	δ_H, multi., *J* in Hz	δ_C	δ_H, multi., *J* in Hz	δ_C	δ_H, multi., *J* in Hz	δ_C	δ_H, multi., *J* in Hz
1	-	165.1	-	172.2	-	168.2	-
2	-	114.8	-	111.5	-	142.7	-
3	-	155.0	-	156.6	-	156.2	-
3-OH	n. d.	-	n. d.	-	n. d.	-	8.37, brs
4	6.89, d, 8.0	116.8	6.91, d, 8.0	113.5	6.91, d, 8.0	111.6	6.97, d, 8.0
5	7.44, t, 8.0	133.7	7.53, t, 8.0	136.9	7.54, t, 8.0	137.2	7.49, t, 8.0
6	7.34, d, 8.0	115.0	6.92, d, 8.0	115.5	7.12, d, 8.0	115.8	7.32, d, 8.0
7	-	136.3	-	150.0	-	140.1	-
8	-	136.1	5.49, t, 5.5	82.4	-	143.5	-
9	5.39, d, 8.5	113.3	2.63, m	37.5	6.22, d, 11.5	110.0	5.58, d, 8.5
10	4.61, dt, 8.5, 6.5	75.3	5.33, dt, 15.0, 7.5	122.0	6.62, ddt, 15.0, 11.5, 1.5	123.1	5.07, m
11	1.68, m; 1.52, m	35.3	5.57, dt, 15.0, 7.5	136.6	6.04, dt, 15.0, 7.5	140.8	1.77, m; 1.69, m
12	1.34, m	24.6	1.98, m	32.6	2.23, m	33.4	1.37, m
13	1.25, m	28.6	1.30, m[b]	29.0	1.46, m	28.8	1.30, m[d]
14	1.22, m[a]	31.1	1.19, m	31.4	1.33, m[c]	31.5	1.30, m[d]
15	1.22, m[a]	21.9	1.27, m[b]	22.6	1.33, m[c]	22.7	1.30, m[d]
16	0.79, t, 6.5	13.8	0.87, t, 7.5	14.2	0.90, t, 7.5	14.2	0.88, t, 6.5
17	3.19, s	55.3	-	-	-	-	-
2'	3.72, td, 6.5, 5.5	40.8	-	-	-	-	3.91, m
3'	3.49, t, 6.5	58.3	-	-	-	-	1.84, m
3'-OH	n. d.	-	-	-	-	-	-
4'	-	-	-	-	-	-	3.57, m
4'-OH	-	-	-	-	-	-	n. d.

n. d.: Signals not detected. [a, b, c, d] Signals with the same letter overlapping with each other.

Table S8. ^{1}H (500 MHz) and ^{13}C NMR (125 MHz) spectroscopic data of roquesalin G (**9**) in DMSO-d_6, and roquesalin H (**10**) in $CDCl_3$ with key HMBC and ^{1}H-^{1}H COSY correlations.

Compound

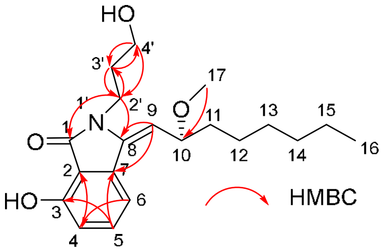

roquesalin G

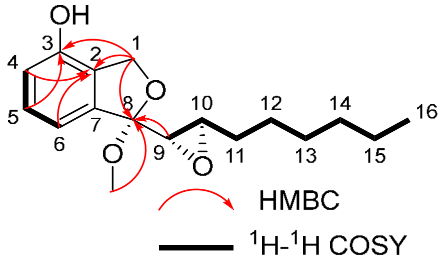

roquesalin H

Position	δ_H, multi., *J* in Hz	δ_C	δ_H, multi., *J* in Hz	δ_C
1	-	164.9	5.08, d, 3.0	71.8
1-OH	-	-	-	-
2	-	114.8	-	127.1
3	-	154.9	-	150.4
3-OH	9.91, br s	-	n. d.	-
4	6.93, d, 8.0	116.9	6.45, dd, 8.0, 0.5	115.7
5	7.46, t, 8.0	133.7	7.03, t, 7.5	129.8
6	7.38, d, 8.0	115.1	6.86, dd,7.5, 0.5	114.3
7	-	136.2	-	137.3
8	-	135.8	-	110.5
9	5.40, d, 8.5	113.0	3.29, d, 2.0	61.2
10	4.64, dt, 8.5, 6.5	75.3	3.30, m	56.6
11	1.73, m; 1.52, m	35.3	1.66, m; 1.58, m	31.3
12	1.37, m	24.6	1.46, m	25.9
13	1.27, m	28.6	1.35, m	29.1
14	1.23, m[a]	31.1[b]	1.24, m	31.8
15	1.23, m[a]	21.9	1.28, m	22.6
16	0.82, t, 6.5	13.8	0.87, t, 7.0	14.1
17	3.22, s	55.3	3.06, s	50.2
2'	3.71, m	31.1[b]	-	-
3'	1.68, m	35.3	-	-
4'	3.44, td, 6.5, 5.5	58.3	-	-
4'-OH	4.53, br s	-	-	-

n. d.: Signals not detected. [a, b] Signals with the same letter overlapping with each other.

Table S9. ^{1}H (500 MHz) and ^{13}C NMR (125 MHz) spectroscopic data of roquesalin I (**12**) in $CDCl_3$ and roquesalin J (**14**) DMSO-d_6 in with key HMBC and ^{1}H-^{1}H COSY correlations.

Compound

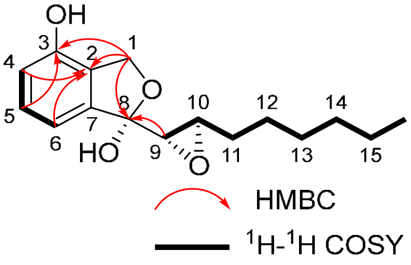

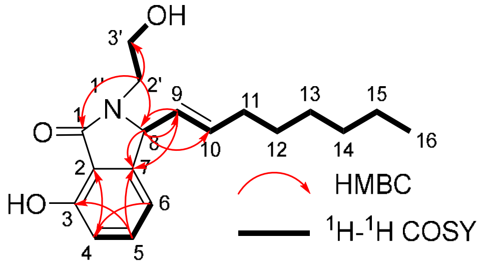

	roquesalin I		roquesalin J	
Position	δ_H, multi., *J* in Hz	δ_C	δ_H, multi., *J* in Hz	δ_C
1	5.01, d, 13.0; 4.98, d, 13.0	70.3	-	167.6[b]
2	-	126.9	-	116.6
3	-	150.7	-	157.3[b]
3-OH	n. d.	-	n. d.	-
4	6.68, d, 7.0	116.4	6.69, d, 8.0	115.9[c]
5	7.13, t, 8.0	129.9	7.27, t, 8.0	132.9
6	6.83, d,7.0	114.4	6.62, d, 8.0	112.0[c]
7	-	140.0	-	147.1
8	-	106.1	4.97, d, 9.5	63.0
9	3.15, d, 2.0	61.0	5.08, dd, 15.0, 9.5	127.0
10	3.21, m	56.3	5.97, dt, 15.0, 7.5	136.5
11	1.62, m; 1.55, m	31.4	2.06, dt,7.5, 7.0	31.5
12	1.44, m	26.0	1.38, m	28.4
13	1.33, m	29.2	1.29, m	28.0
14	1.27, m	31.8	1.25, m[a]	30.9
15	1.29, m	22.7	1.25, m[a]	22.0
16	0.87, t, 6.5	14.2	0.85, t, 6.5	13.8
2'	-	-	3.67, dt, 14.5, 5.5; 3.08, dt, 14.5, 5.5	42.8[b]
3'	-	-	3.51, m	58.8
3'-OH	n. d.	-	n. d.	-

n. d.: Signals not detected. [a] Signals overlapping with each other. [b] Signals were deduced from in HMBC correlations. [c] Signals were deduced from HSQC correlations.

Table S10. Conformational analysis of the optimized isomers of **4** in methanol.

Conformations	*G* (hartree)	*ΔG* (kcal/mol)	Boltzmann distributions (%)
**4**-1	-1056.616626	0	23.9
**4**-2	-1056.616431	0.122364333	19.4
**4**-3	-1056.616244	0.239708591	15.9
**4**-4	-1056.615916	0.445531674	11.3
**4**-5	-1056.615849	0.487574804	10.5
**4**-6	-1056.615703	0.579191176	9.0
**4**-7	-1056.615369	0.788779316	6.3
**4**-8	-1056.614853	1.112574166	3.6

Table S11. The coordinates of the optimized conformers of **4**.

	**4**-1			**4**-2			**4**-3			**4**-4		
	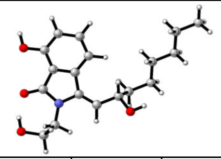			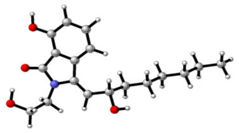			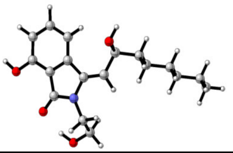			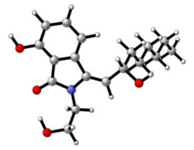		
C	0.96564	3.54026	-0.1246	-1.72594	3.56632	0.53583	-4.19503	-2.03031	1.38047	3.13352	-3.1775	-0.48534
C	-0.23835	2.84794	-0.22412	-0.49637	3.04093	0.92468	-3.14609	-2.84791	0.96623	2.03754	-3.38661	0.3477
C	-0.29342	1.45413	-0.16925	-0.21507	1.67647	0.83244	-2.05076	-2.34287	0.2615	1.22232	-2.33525	0.77102
C	0.90289	0.75956	-0.01337	-1.20996	0.83752	0.33779	-2.03171	-0.97894	-0.0083	1.53719	-1.05037	0.33755
C	2.12221	1.45243	0.09456	-2.45169	1.36227	-0.06341	-3.0997	-0.15064	0.3778	2.6408	-0.83224	-0.50697
C	2.1715	2.84736	0.03631	-2.72816	2.72796	0.03303	-4.1917	-0.66169	1.08174	3.45339	-1.88805	-0.92677
O	3.36664	3.47309	0.13964	-3.9382	3.18729	-0.36088	-5.19741	0.16992	1.43978	4.50568	-1.63244	-1.73804
C	1.19927	-0.68375	0.06929	-1.25153	-0.62213	0.12434	-1.03899	-0.13579	-0.70146	0.9106	0.2592	0.6024
N	2.60018	-0.76903	0.27325	-2.52287	-0.88281	-0.44785	-1.62951	1.14624	-0.78885	1.66526	1.1961	-0.14945
C	3.20616	0.46981	0.25496	-3.2947	0.25606	-0.54412	-2.8285	1.21404	-0.10436	2.73062	0.60755	-0.79776
O	4.42103	0.64614	0.34755	-4.45696	0.27126	-0.94967	-3.47982	2.24654	0.04319	3.56228	1.22566	-1.46254
C	0.38914	-1.7464	-0.04561	-0.34715	-1.56997	0.41088	0.20128	-0.43274	-1.1199	-0.1224	0.59021	1.39103
C	-1.09647	-1.73261	-0.23562	1.01926	-1.37031	0.99135	0.87936	-1.763	-0.95432	-0.98604	-0.33588	2.19376
C	-1.83224	-1.57829	1.1079	2.04475	-0.97633	-0.08405	2.39372	-1.64786	-1.12865	-2.09527	-0.99636	1.35463
C	-3.36083	-1.55007	0.99712	3.45054	-0.74047	0.46987	3.06942	-0.79983	-0.04948	-3.04572	-0.00591	0.68295
C	3.35518	-1.99339	0.52703	-2.99339	-2.17678	-0.93529	-1.06258	2.30311	-1.47649	1.35905	2.61613	-0.30249
C	4.21432	-2.4436	-0.65337	-4.04481	-2.82739	-0.03772	-0.45479	3.34162	-0.53526	2.31423	3.53758	0.45444
O	5.438	-1.72542	-0.75042	-5.34734	-2.29258	-0.23753	-1.43404	4.18605	0.05786	3.54112	3.74531	-0.23389
O	-1.41381	-2.98564	-0.86038	1.35839	-2.62292	1.60488	0.31476	-2.67202	-1.92006	-1.53573	0.47388	3.24342
C	-3.91879	-0.33247	0.25548	4.43939	-0.27286	-0.59914	4.58658	-0.71236	-0.22178	-4.17381	-0.69611	-0.08395
C	-5.44733	-0.29494	0.23056	5.85165	-0.04634	-0.05831	5.2684	0.13282	0.85482	-5.13027	0.28543	-0.76255
C	-6.01229	0.92015	-0.5075	6.8437	0.42052	-1.12537	6.7863	0.22448	0.6865	-6.26511	-0.39906	-1.5269
C	-7.54071	0.95119	-0.52677	8.25289	0.6437	-0.57581	7.45848	1.07116	1.76769	-7.21546	0.59063	-2.20148
H	0.97365	4.62468	-0.16812	-1.91159	4.63265	0.61735	-5.03216	-2.45608	1.92503	3.74462	-4.01743	-0.80031
H	-1.15759	3.41034	-0.34304	0.26327	3.71422	1.3055	-3.1902	-3.90758	1.19157	1.81138	-4.39645	0.67095
H	-1.2489	0.95848	-0.2392	0.75508	1.31275	1.13478	-1.27971	-3.0034	-0.10519	0.37639	-2.542	1.4085
H	3.24422	4.42914	0.08012	-3.98286	4.1441	-0.23882	-5.88227	-0.3235	1.90851	4.97228	-2.45333	-1.94056
H	0.80502	-2.74562	0.02022	-0.57641	-2.60851	0.20017	0.7901	0.34368	-1.59476	-0.41161	1.63109	1.47571
H	-1.38224	-0.9244	-0.91568	0.99687	-0.59725	1.76869	0.67912	-2.14851	0.05462	-0.37556	-1.12451	2.64799
H	-1.52236	-2.41139	1.74692	2.06525	-1.76877	-0.84011	2.81834	-2.65876	-1.11032	-1.62706	-1.63302	0.59672
H	-1.48297	-0.66274	1.59652	1.69165	-0.07127	-0.58908	2.60037	-1.23945	-2.1248	-2.66264	-1.66816	2.01202
H	-3.71869	-2.47046	0.51924	3.40576	0.00443	1.275	2.64773	0.21201	-0.05261	-3.46928	0.65754	1.44363
H	-3.77763	-1.57092	2.01	3.83259	-1.66308	0.92295	2.83772	-1.22002	0.9372	-2.47883	0.63381	-0.00336
H	4.0004	-1.82101	1.39247	-3.41701	-2.03	-1.93224	-0.31731	1.94125	-2.1826	1.39853	2.85918	-1.36759
H	2.64773	-2.77292	0.80248	-2.12939	-2.82913	-1.04481	-1.85929	2.77226	-2.05907	0.33449	2.7763	0.02698
H	4.47307	-3.49792	-0.52326	-4.09933	-3.89455	-0.26939	0.14085	2.8259	0.23073	1.84464	4.51801	0.57141
H	3.62645	-2.35452	-1.57747	-3.73041	-2.72781	1.01048	0.22034	3.98678	-1.10383	2.48587	3.12636	1.45891
H	5.27043	-0.81671	-0.44584	-5.24792	-1.34988	-0.45631	-2.2416	3.65955	0.18448	3.75608	2.92209	-0.70583
H	-2.33949	-2.96589	-1.1272	2.16937	-2.50066	2.11039	0.75797	-3.52364	-1.81332	-2.1175	-0.08317	3.77414
H	-3.54217	0.5822	0.73075	4.47362	-1.01316	-1.40826	5.01188	-1.72394	-0.21379	-3.74412	-1.36595	-0.83989
H	-3.54993	-0.31307	-0.7772	4.06937	0.65553	-1.05194	4.81507	-0.295	-1.21061	-4.74015	-1.33843	0.60269
H	-5.82469	-1.21268	-0.23857	5.81619	0.69371	0.75164	4.84179	1.14423	0.84679	-5.55648	0.95748	-0.00641
H	-5.82687	-0.30298	1.2603	6.21944	-0.97522	0.39645	5.03854	-0.28429	1.84384	-4.56465	0.92594	-1.45165
H	-5.63225	1.83576	-0.03825	6.87814	-0.31927	-1.93435	7.21208	-0.78629	0.69439	-5.83817	-1.07048	-2.28187
H	-5.63189	0.92672	-1.53621	6.47553	1.34862	-1.57926	7.01537	0.6417	-0.30166	-6.82998	-1.03869	-0.8378
H	-7.91877	1.8284	-1.05903	8.94189	0.9759	-1.35729	8.54155	1.12076	1.62512	-8.01651	0.07679	-2.74016
H	-7.94768	0.06308	-1.02039	8.25411	1.4036	0.212	7.07605	2.09673	1.76109	-7.68265	1.25348	-1.4663
H	-7.94819	0.97855	0.48872	8.65937	-0.27679	-0.14506	7.274	0.65825	2.76447	-6.68371	1.22115	-2.92107

	**4**-5			**4**-6			**4**-7			**4**-8		
	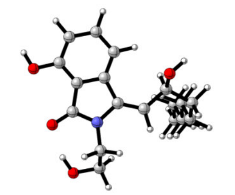			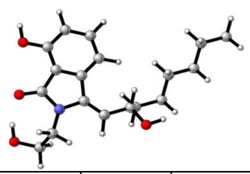			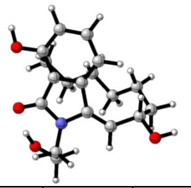			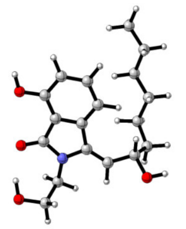		
C	-3.07892	-2.3306	1.79854	1.00135	3.55403	0.03049	-2.02503	-3.01265	-0.55234	-0.65435	-3.06358	1.03823
C	-2.08832	-3.03513	1.11854	-0.21332	2.88473	-0.09586	-0.68908	-3.40754	-0.55928	-1.40976	-1.95849	1.42163
C	-1.27497	-2.42503	0.16045	-0.27887	1.49853	-0.24601	0.3481	-2.49463	-0.75186	-0.96724	-0.65327	1.20477
C	-1.47666	-1.07185	-0.08783	0.91811	0.78832	-0.27935	0.01597	-1.15831	-0.95173	0.26261	-0.47101	0.58145
C	-2.49566	-0.35999	0.56816	2.14842	1.45718	-0.14911	-1.33274	-0.75956	-0.9825	1.03676	-1.58325	0.20502
C	-3.30605	-0.97577	1.52375	2.20768	2.84402	0.00927	-2.36656	-1.67393	-0.76928	0.59294	-2.8893	0.42758
O	-4.27438	-0.25532	2.13569	3.41359	3.44485	0.13337	-3.6468	-1.23389	-0.77135	1.37383	-3.92611	0.04515
C	-0.80214	-0.13959	-1.01137	1.20422	-0.65326	-0.41601	0.84345	0.05371	-1.12939	1.01173	0.74737	0.20345
N	-1.54211	1.06273	-0.93936	2.61764	-0.76256	-0.36872	-0.08128	1.09868	-1.3834	2.21343	0.27576	-0.38786
C	-2.51642	1.01481	0.04054	3.22959	0.45959	-0.18435	-1.38893	0.6892	-1.22523	2.29322	-1.10115	-0.38736
O	-3.21499	1.97323	0.3645	4.44537	0.61254	-0.06548	-2.35673	1.44859	-1.26293	3.26185	-1.73617	-0.8036
C	0.31428	-0.30729	-1.73733	0.37237	-1.69773	-0.54083	2.15457	0.27476	-0.95573	0.72192	2.05031	0.34126
C	1.17835	-1.5344	-1.71931	-1.1247	-1.66333	-0.57429	3.2027	-0.67032	-0.46275	-0.53028	2.69684	0.84307
C	2.59881	-1.22798	-2.19974	-1.71341	-1.63646	0.84789	3.44224	-0.5298	1.05721	-1.41874	3.16331	-0.32877
C	3.3431	-0.17921	-1.36391	-3.24509	-1.61187	0.90229	2.23645	-0.89628	1.94003	-2.07355	2.02437	-1.11768
C	-1.30114	2.26212	-1.73665	3.38891	-1.9865	-0.57224	0.25463	2.48248	-1.70187	3.2385	1.0973	-1.0273
C	-0.57896	3.3724	-0.97538	4.03937	-2.52206	0.70229	0.23822	3.40802	-0.49135	4.5272	1.21189	-0.21554
O	-1.45016	4.11847	-0.13365	5.23956	-1.83764	1.03897	-1.07359	3.62654	0.02286	5.36479	0.06974	-0.34713
O	0.56107	-2.53407	-2.55614	-1.51895	-2.85126	-1.2776	4.39587	-0.33399	-1.18587	-0.0892	3.82697	1.61221
C	3.49318	-0.53879	0.11679	-3.8789	-0.34795	0.31516	1.25772	0.25944	2.20832	-3.30548	1.42374	-0.43109
C	4.34858	0.46574	0.88969	-5.40086	-0.3177	0.46082	-0.16492	-0.21096	2.51885	-3.72692	0.0798	-1.02426
C	4.48676	0.12916	2.37569	-6.04165	0.9399	-0.12935	-1.17344	0.93349	2.64482	-4.8944	-0.57371	-0.28401
C	5.34297	1.1381	3.14149	-7.56291	0.9636	0.02106	-2.61958	0.44419	2.73565	-5.26837	-1.94281	-0.85203
H	-3.69523	-2.83586	2.53553	1.01671	4.63267	0.15045	-2.80764	-3.74556	-0.38432	-1.03011	-4.06622	1.21567
H	-1.95555	-4.08923	1.33486	-1.13251	3.45915	-0.07235	-0.45193	-4.4536	-0.40199	-2.37109	-2.11934	1.89615
H	-0.55791	-3.00394	-0.40199	-1.24189	1.01974	-0.32978	1.37016	-2.84137	-0.74212	-1.58475	0.17239	1.51744
H	-4.75704	-0.8133	2.75874	3.29851	4.3986	0.23129	-4.24889	-1.9695	-0.60083	0.9428	-4.76214	0.26368
H	0.66021	0.51033	-2.3591	0.77874	-2.70191	-0.58657	2.52654	1.28854	-1.06534	1.45173	2.78207	0.01084
H	1.2306	-1.92269	-0.69644	-1.47154	-0.79456	-1.14139	2.94	-1.70847	-0.67814	-1.09618	2.03015	1.49668
H	3.16812	-2.16544	-2.19284	-1.33881	-2.52059	1.37369	4.28795	-1.18486	1.29825	-2.19681	3.82098	0.07985
H	2.54039	-0.90433	-3.24486	-1.31323	-0.76494	1.37631	3.77571	0.49379	1.26083	-0.79925	3.78406	-0.98364
H	4.33761	-0.03526	-1.80088	-3.65087	-2.49794	0.39882	1.69669	-1.72852	1.47779	-2.36266	2.3913	-2.10764
H	2.83731	0.78965	-1.44753	-3.55146	-1.70994	1.94951	2.60059	-1.28358	2.89647	-1.33235	1.23856	-1.29937
H	-0.73461	1.96964	-2.61893	2.72674	-2.73407	-1.00387	1.23252	2.49793	-2.18189	3.46796	0.65482	-2.00014
H	-2.26796	2.63606	-2.08229	4.16793	-1.7791	-1.31059	-0.47268	2.8323	-2.43792	2.81649	2.0825	-1.21307
H	0.24825	2.93337	-0.40037	3.30981	-2.47337	1.52265	0.87686	2.99425	0.29796	5.09926	2.07143	-0.5757
H	-0.14901	4.07605	-1.69328	4.30298	-3.57256	0.55246	0.65778	4.3754	-0.786	4.27124	1.39696	0.83685
H	-2.13718	3.51315	0.19308	5.13523	-0.90969	0.76565	-1.68148	2.98757	-0.38613	4.78998	-0.70491	-0.47267
H	1.12803	-3.31622	-2.54131	-2.46565	-2.80158	-1.44999	5.11205	-0.88296	-0.84488	-0.87213	4.31669	1.89122
H	2.50563	-0.5979	0.58843	-3.45012	0.53305	0.80948	1.6384	0.87641	3.03117	-3.11519	1.29236	0.63961
H	3.93431	-1.53997	0.20649	-3.62587	-0.25312	-0.74772	1.2187	0.91843	1.33931	-4.13712	2.13593	-0.49252
H	5.34634	0.52016	0.43539	-5.82884	-1.20476	-0.02366	-0.49311	-0.88514	1.72031	-3.98889	0.21065	-2.08208
H	3.9132	1.46807	0.7849	-5.665	-0.39568	1.52319	-0.16995	-0.81027	3.4385	-2.8675	-0.60118	-1.00562
H	3.4888	0.07681	2.82773	-5.61098	1.82506	0.35435	-0.93013	1.53974	3.52596	-4.6312	-0.67814	0.77589
H	4.91929	-0.8734	2.47992	-5.77651	1.01578	-1.19092	-1.07645	1.6052	1.7846	-5.76608	0.09108	-0.31545
H	5.42549	0.87506	4.19972	-7.9956	1.87144	-0.40829	-3.31713	1.27671	2.86411	-6.10359	-2.39147	-0.30711
H	6.35694	1.18723	2.73216	-8.02308	0.10723	-0.48184	-2.91023	-0.09076	1.82643	-5.56041	-1.86875	-1.90428
H	4.91565	2.14408	3.08246	-7.85656	0.92277	1.07468	-2.75819	-0.23814	3.58052	-4.42304	-2.63575	-0.79325

Table S12. Experimental and calculated ^{13}C NMR chemical shifts of **12**.

No.	Experimental (δ_C, ppm)	Calculated (δ_C, ppm)		Corrected (δ_C, ppm)	
		(8*S*,9*R*,10*S*)-**12**	(8*S*,9*S*,10*R*)-**12**	(8*S*,9*R*,10*S*)-**12**	(8*S*,9*S*,10*R*)-**12**
1	70.3	71.9	72.3	70.3	70.8
2	126.9	124.5	126.5	125.5	127.7
3	150.7	147.8	147.8	149.9	150.1
4	116.4	113.0	112.6	113.4	113.1
5	129.9	128.3	127.7	129.5	129.0
6	114.4	113.3	113.8	113.8	114.4
7	140	140.4	139.4	142.2	141.3
8	106.1	108.5	108.0	108.7	108.3
9	61	65.0	64.2	63.1	62.3
10	56.3	60.4	58.0	58.3	55.8
11	31.4	33.4	34.1	29.9	30.7
12	26	29.8	30.2	26.2	26.6
13	29.2	32.2	32.9	28.6	29.4
14	31.8	35.6	35.8	32.3	32.5
15	22.7	27.2	26.9	23.4	23.2
16	14.2	16.4	16.5	12.1	12.3
**R^2**		**0.9989**	**0.9992**		
**MAE**		**2.70**	**2.52**		
**CMAE**				**1.30**	**1.00**

Table S13. Experimental and calculated ^{1}H NMR chemical shifts of **12**.

No.	Experimental (δ_H, ppm)	Calculated (δ_H, ppm)		Corrected (δ_H, ppm)	
		(8*S*,9*R*,10*S*)-**12**	(8*S*,9*S*,10*R*)-**12**	(8*S*,9*R*,10*S*)-**12**	(8*S*,9*S*,10*R*)-**12**
1	5.01	5.22	5.33	5.02	5.10
4	6.68	6.97	6.97	6.57	6.62
5	7.13	7.65	7.61	7.18	7.21
6	6.83	7.37	7.20	6.92	6.83
9	3.15	2.92	3.03	2.97	2.98
10	3.21	3.22	3.21	3.24	3.14
11a	1.62	2.00	1.83	2.16	1.87
11b	1.55	1.00	1.26	1.28	1.34
12	1.44	1.33	1.45	1.57	1.52
13	1.33	0.70	1.22	1.00	1.30
14	1.27	0.95	1.19	1.23	1.27
15	1.29	0.83	1.10	1.12	1.19
16	0.87	0.81	0.89	1.11	1.00
		**0.9909**	**0.9972**		
		**0.33**	**0.19**		
				**0.17**	**0.10**

Supplementary Figures

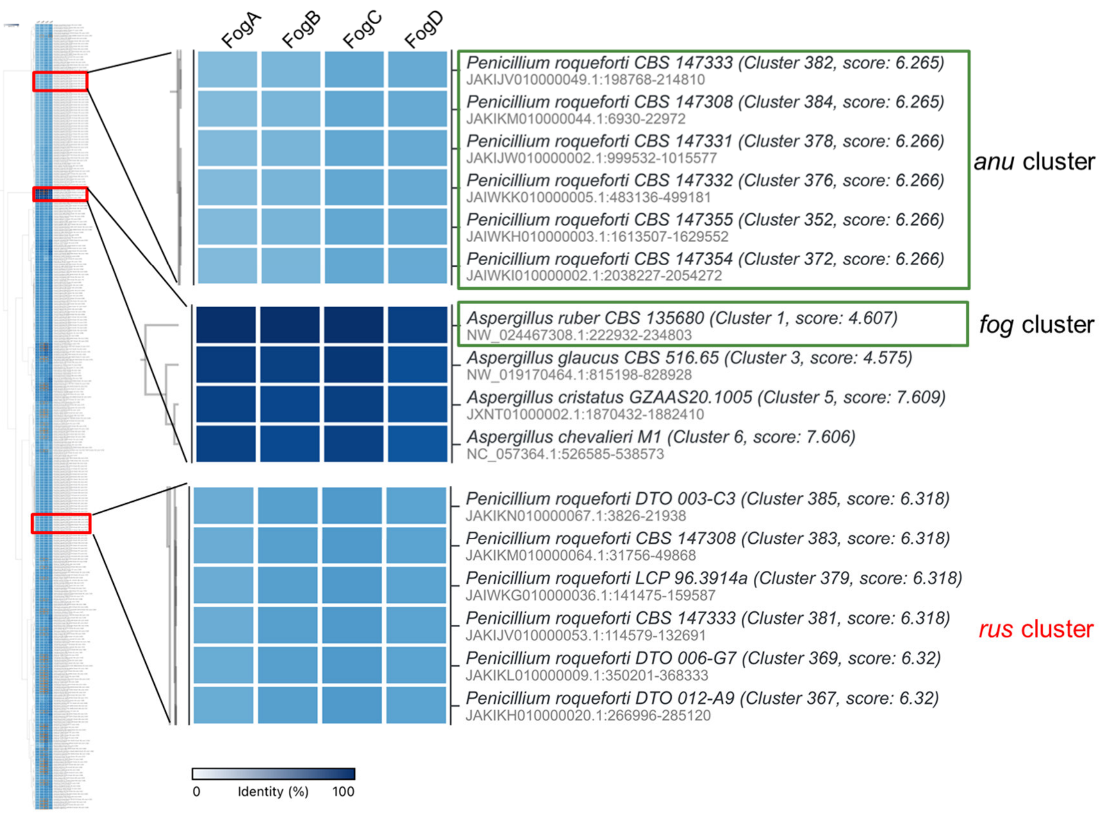

Figure S1. Results of the cblaster analysis against the RefSeq database using the sequences of FogABCD as queries.

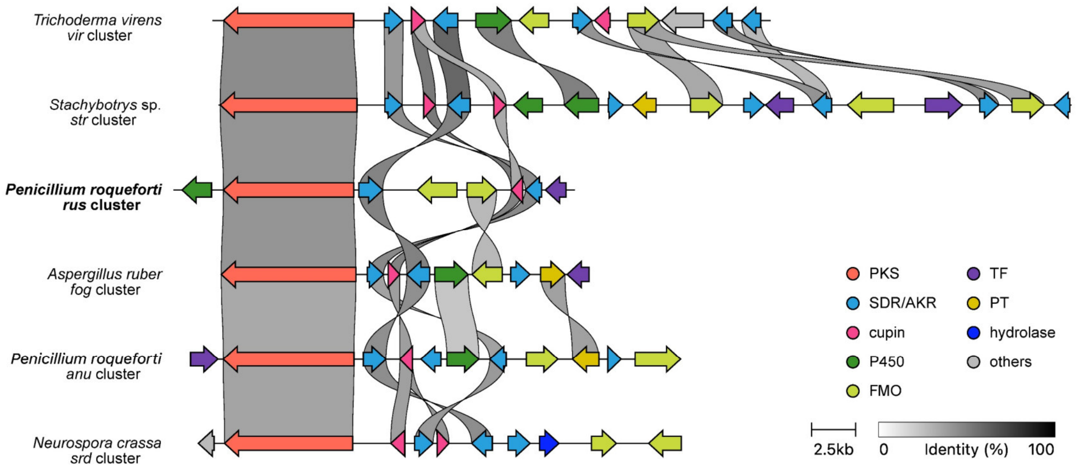

Figure S2. Schematic representation of the *rus* cluster and its homologous gene clusters.
PKS: polyketide synthase; TF: transcription factor; SDR: short-chain dehydrogenase/reductase; AKR: aldo-keto reductase; PT: prenyltransferase; cupin: cupin domain-containing protein; P450: cytochrome P450 monooxygenase; FMO: flavin-containing monooxygenase.

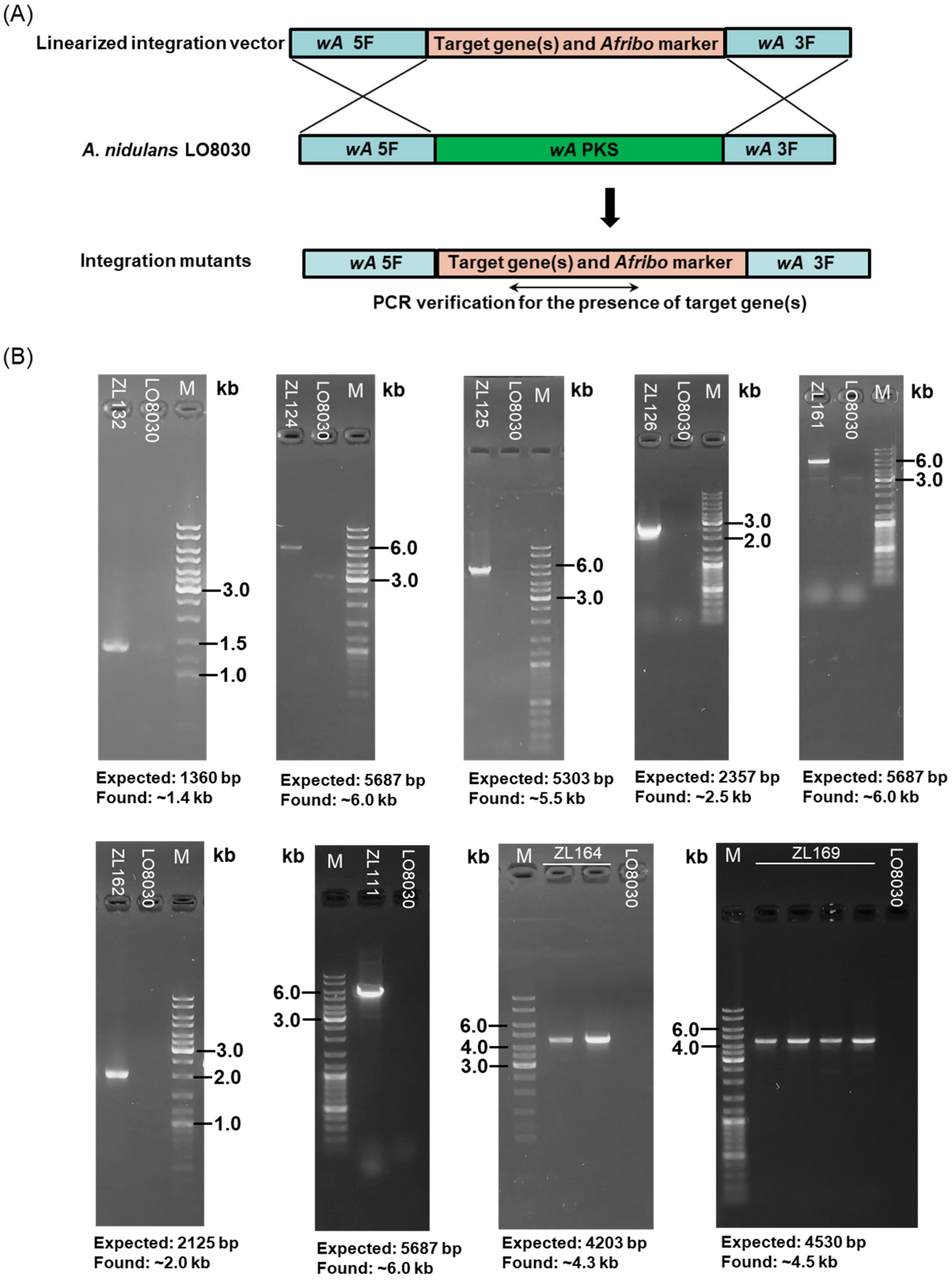

Figure S3. Heterologous expression and PCR verification for *A. nidulans* strains.
(A) Schematic representation of gene integration into the *wA*-PKS locus of *A. nidulans* LO8030. (B) PCR verifications for the presence of the target gene(s) were performed with genomic DNA of the *A. nidulans* transformants using primers listed in Table S2.

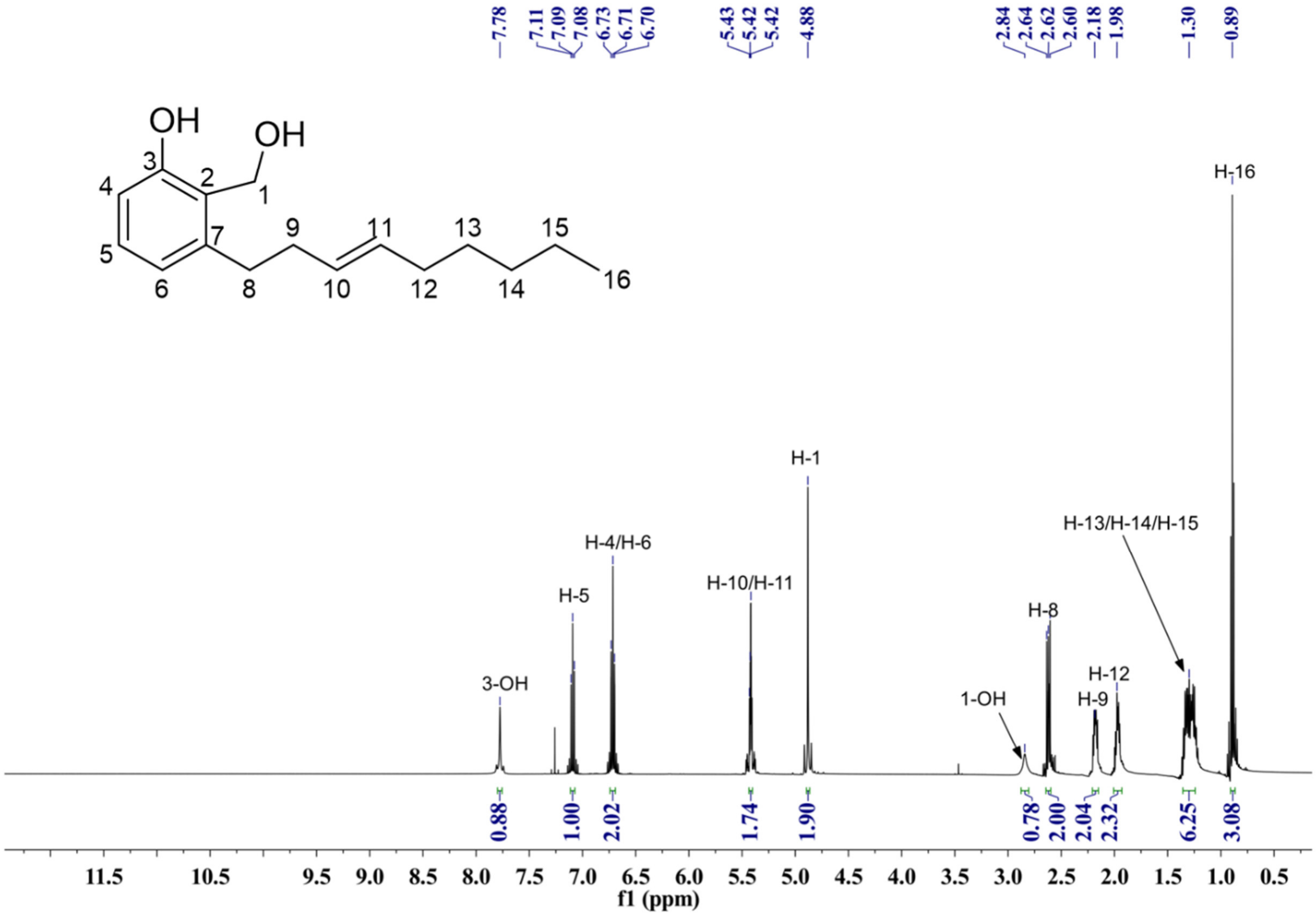

Figure S4. ^{1}H NMR spectrum of stachysalicyloid B (**1**) in $CDCl_3$ (500 MHz).

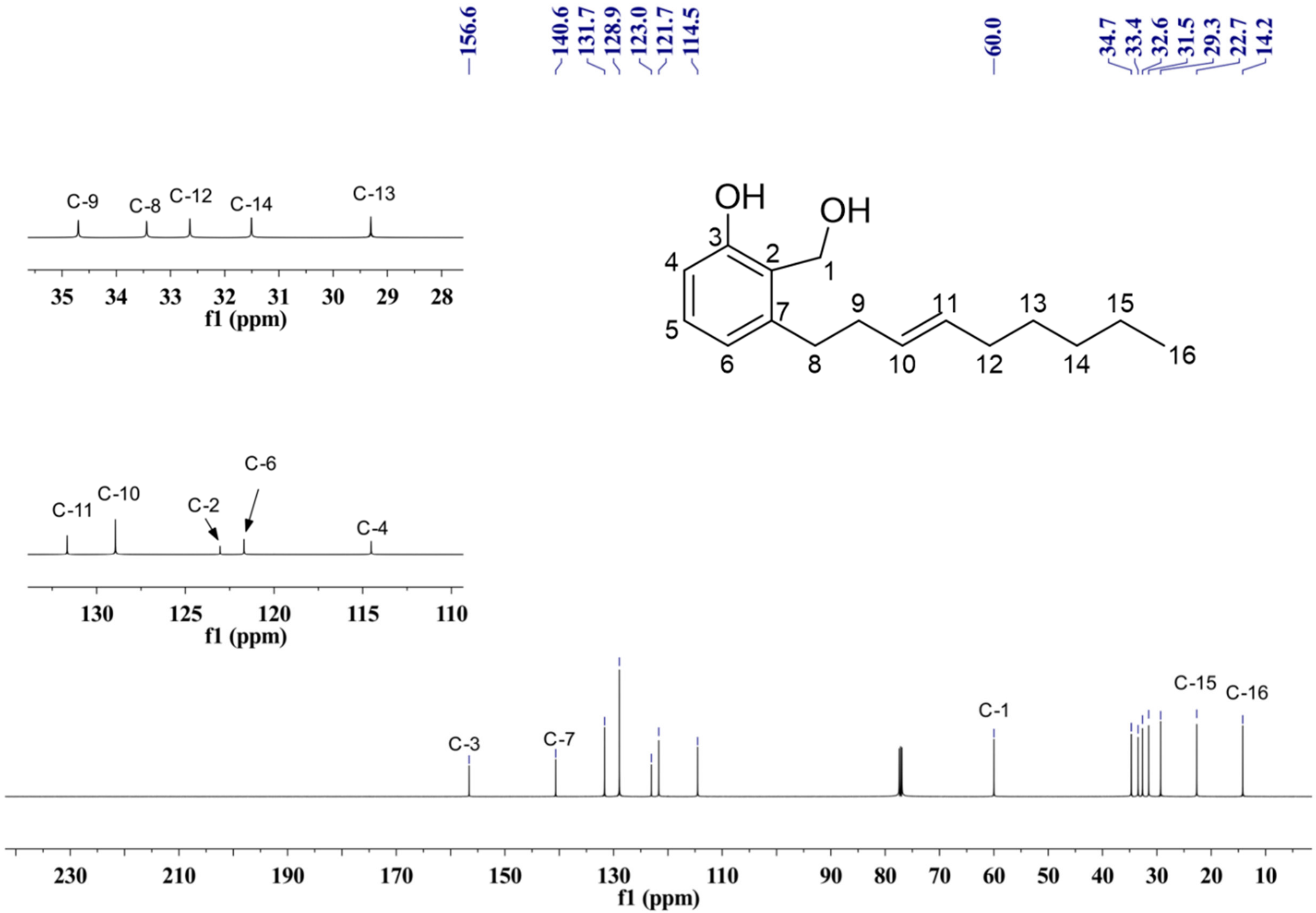

Figure S5. ^{13}C NMR spectrum of stachysalicyloid B (**1**) in $CDCl_3$ (125 MHz).

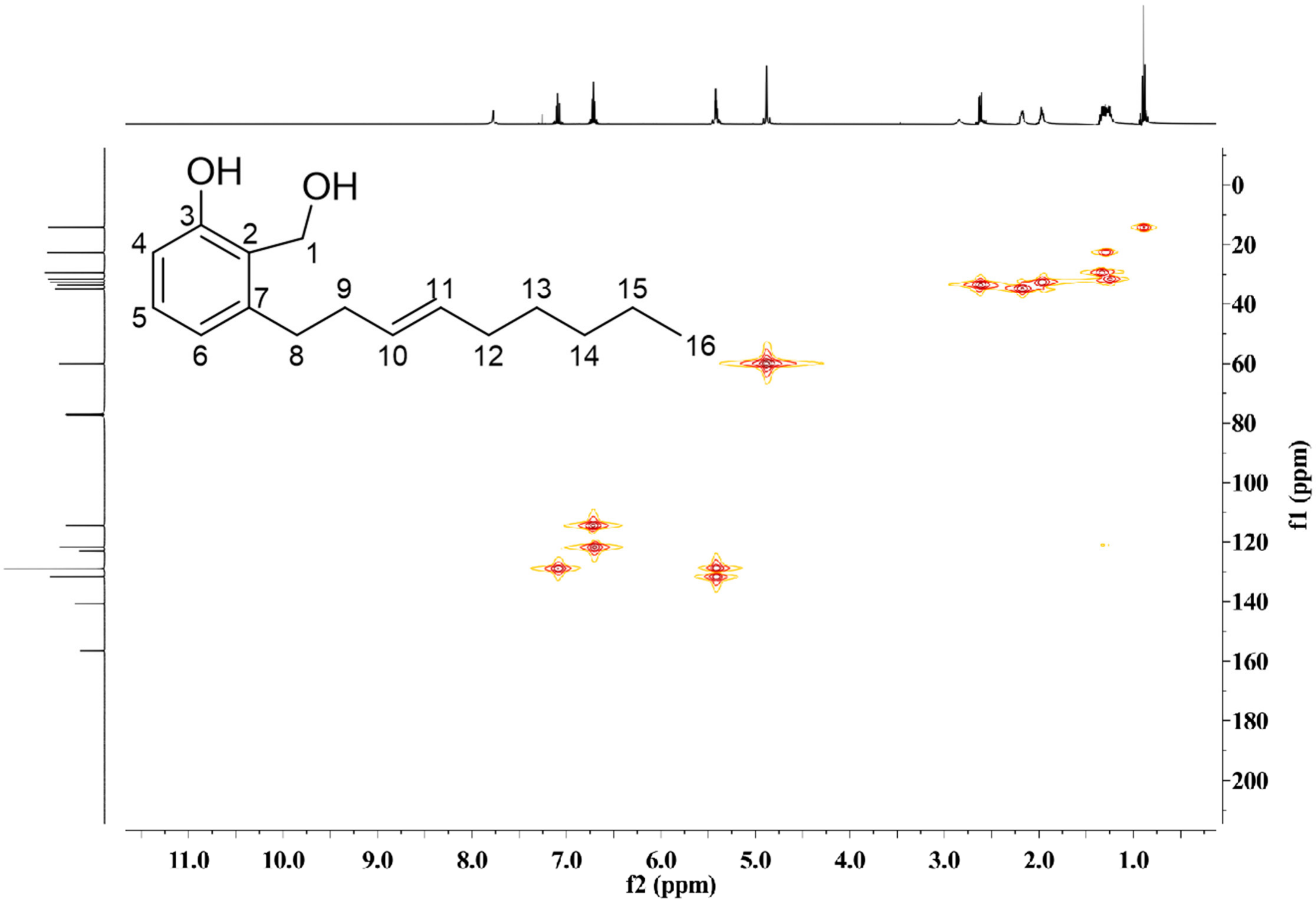

Figure S6. HSQC spectrum of stachysalicyloid B (**1**) in $CDCl_3$.

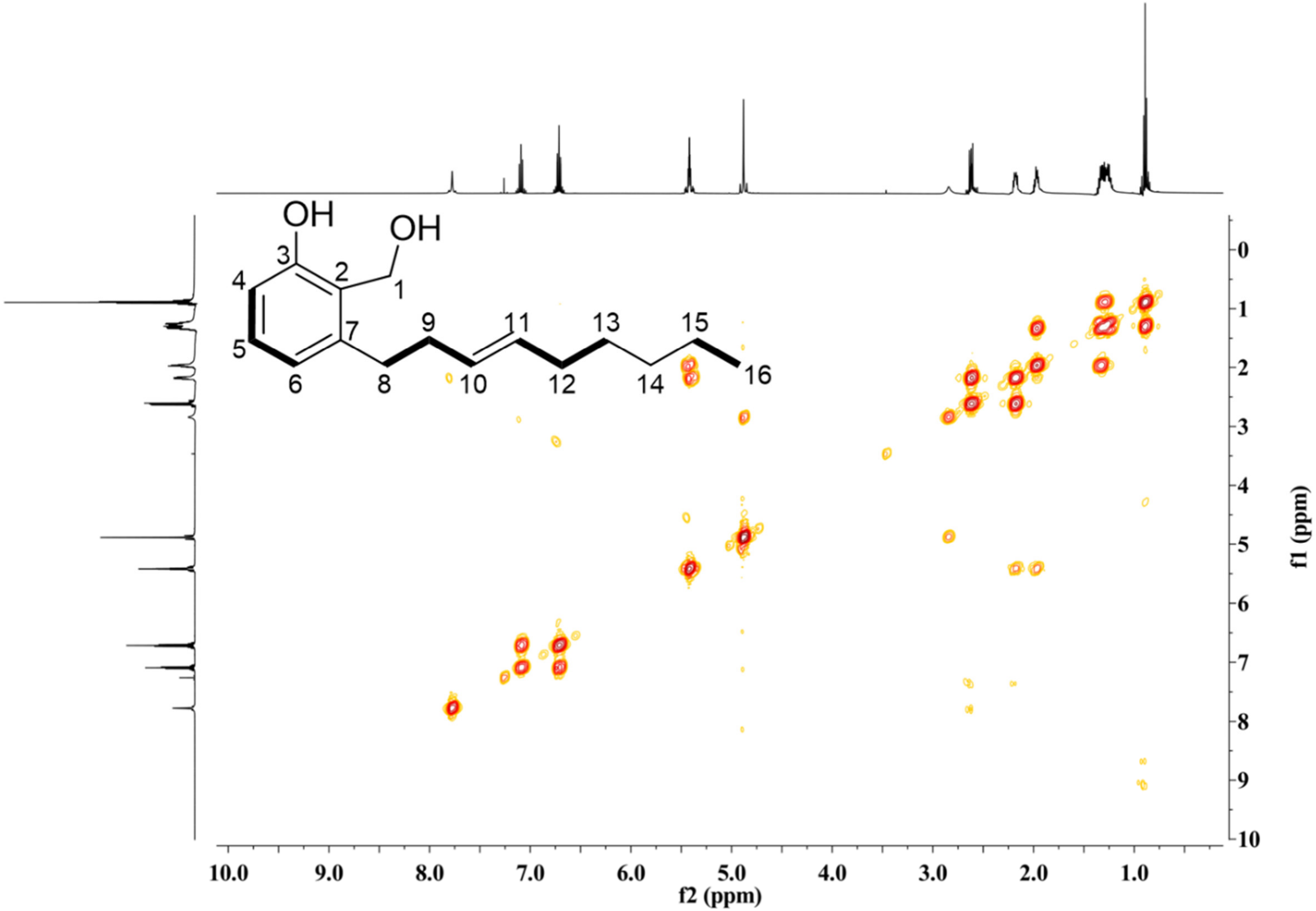

Figure S7. ^1H-^1H COSY spectrum of stachysalicyloid B (**1**) in $CDCl_3$.

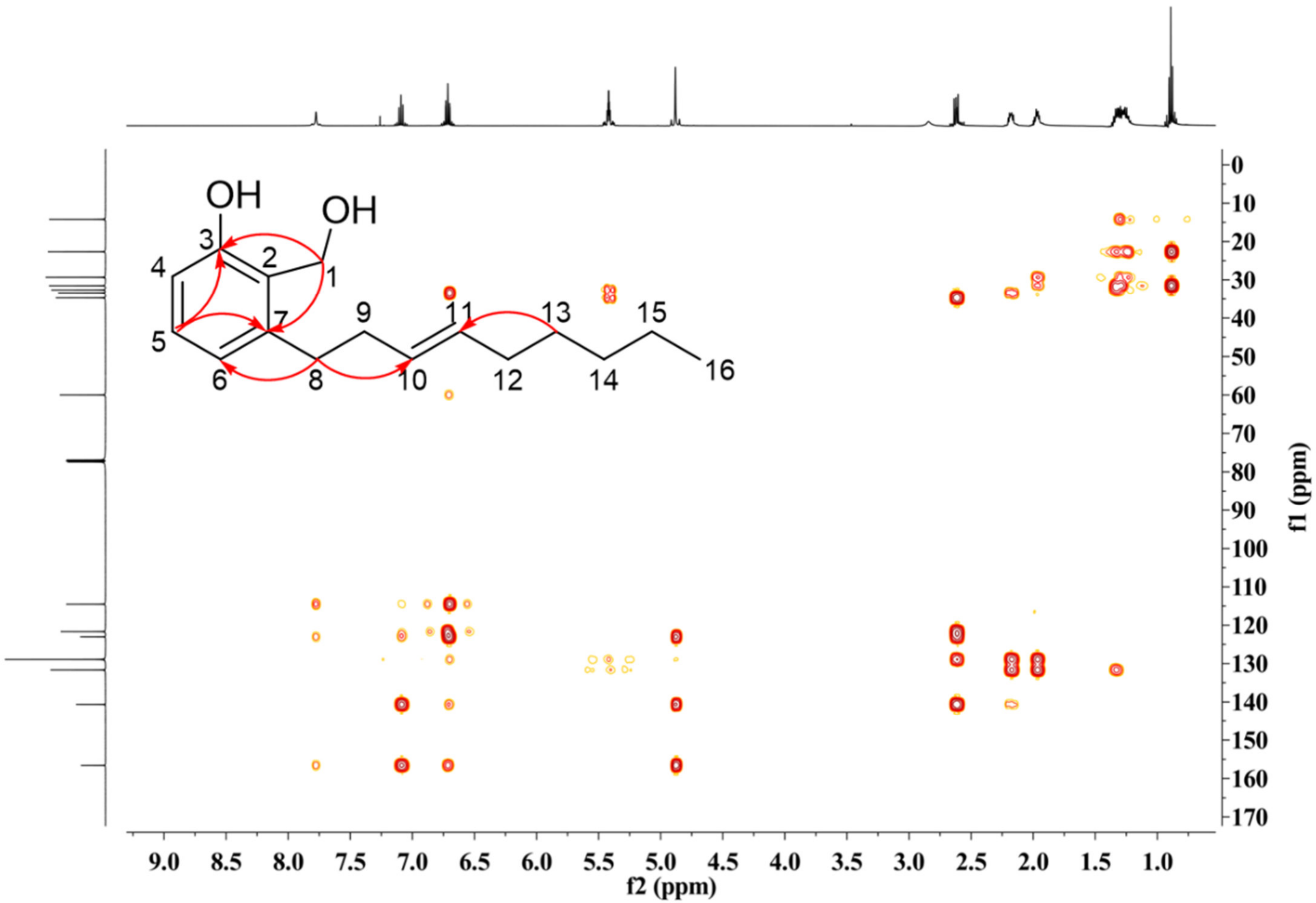

Figure S8. HMBC spectrum of stachysalicyloid B (**1**) in $CDCl_3$.

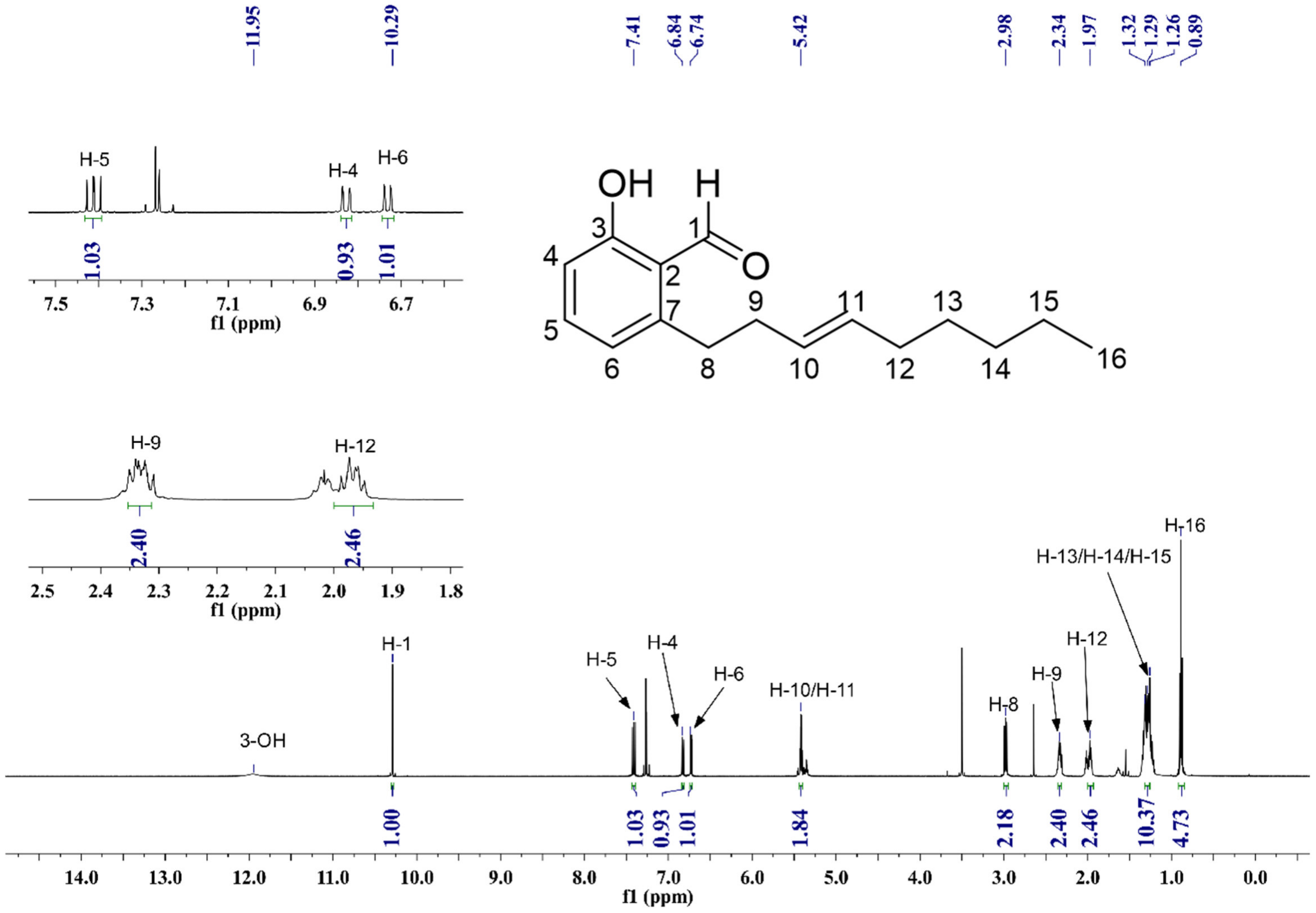

Figure S9. ^{1}H NMR spectrum of stachysalicyloid D (**2**) in $CDCl_3$ (500 MHz).

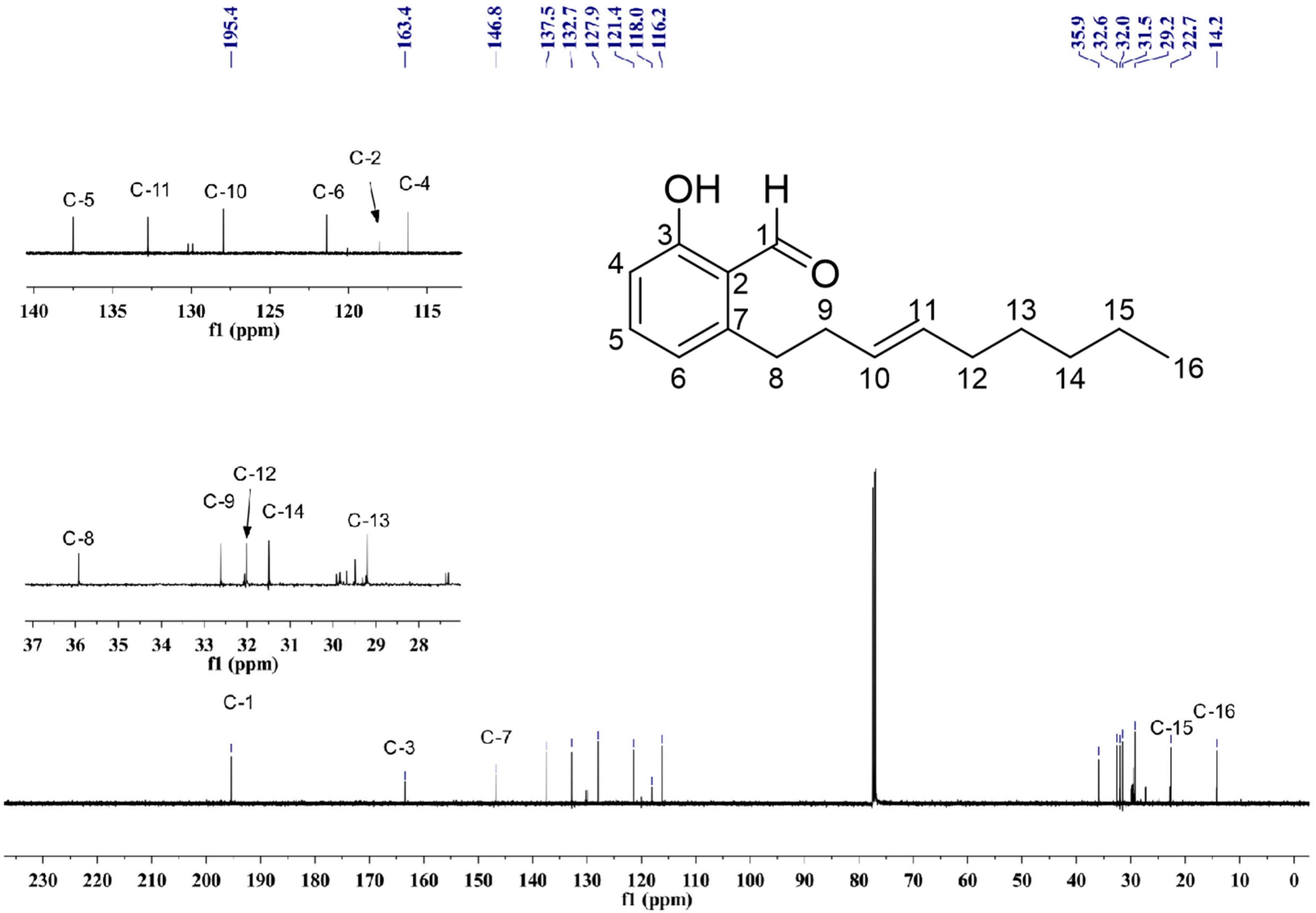

Figure S10. ^{13}C NMR spectrum of stachysalicyloid D (**2**) in $CDCl_3$ (125 MHz).

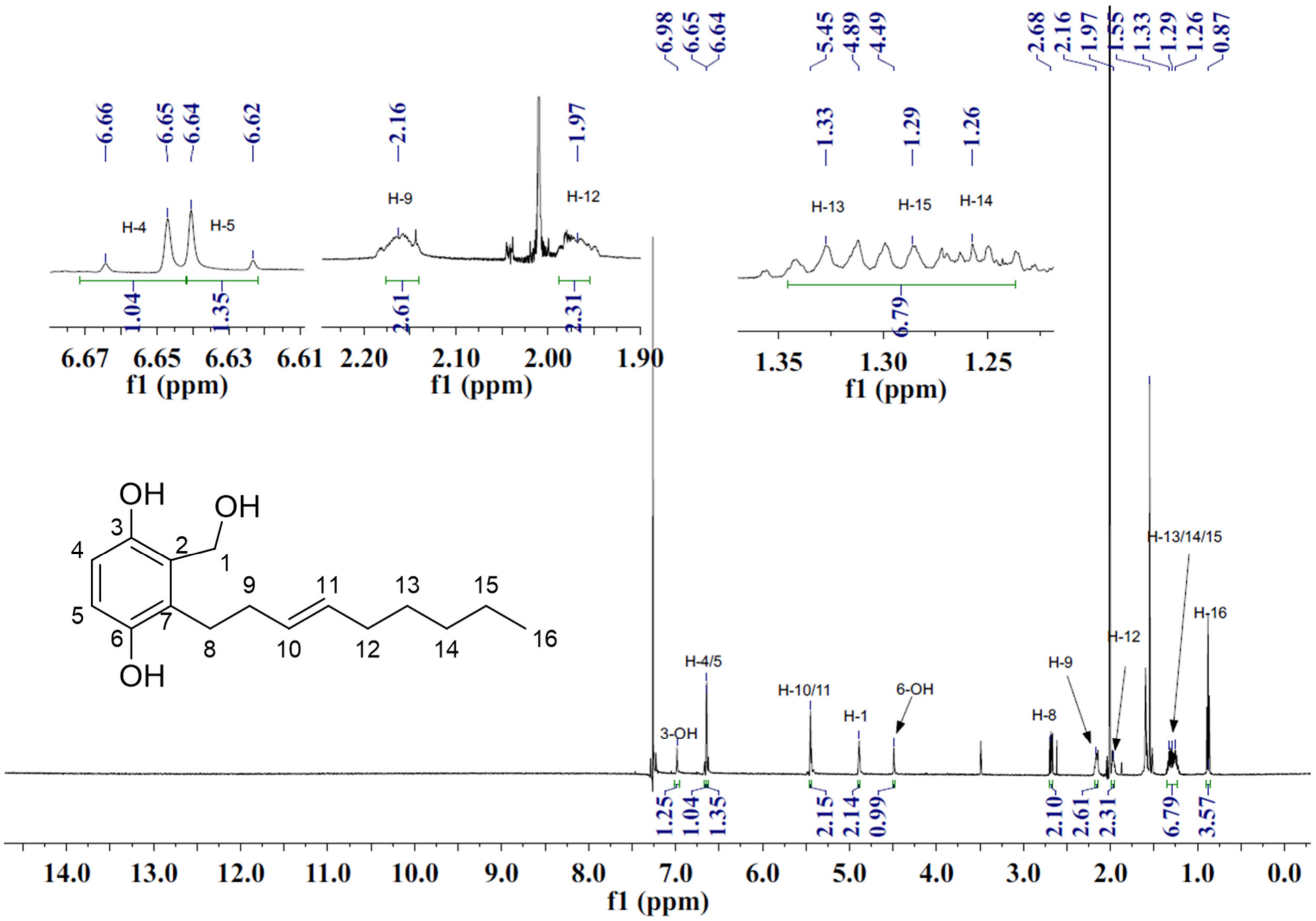

Figure S11. ^{1}H NMR spectrum of roquesalin A (**3**) in $CDCl_3$ (500 MHz).

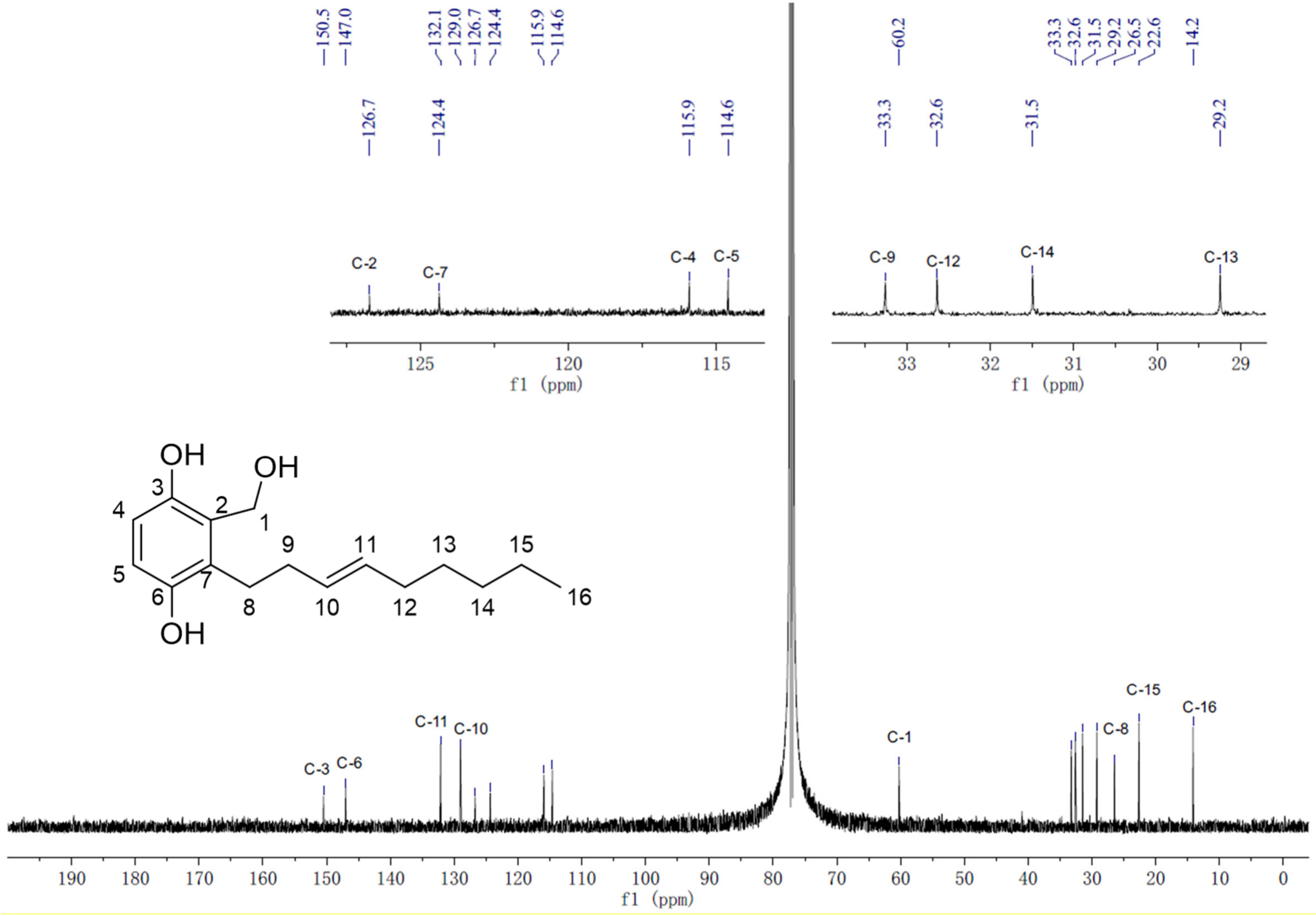

Figure S12. ^{13}C NMR spectrum of roquesalin A (**3**) in $CDCl_3$ (125 MHz).

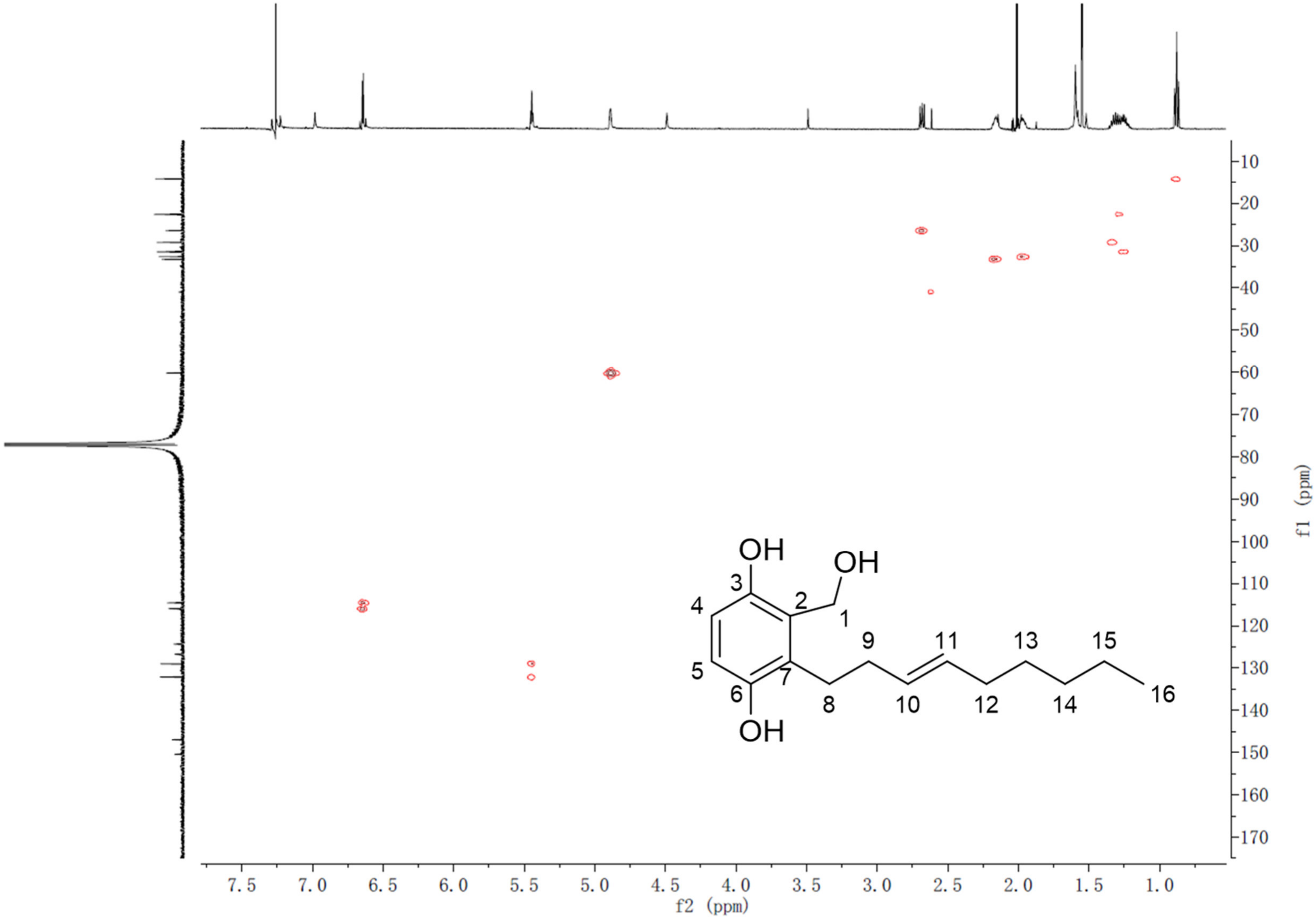

Figure S13. HSQC spectrum of roquesalin A (**3**) in $CDCl_3$.

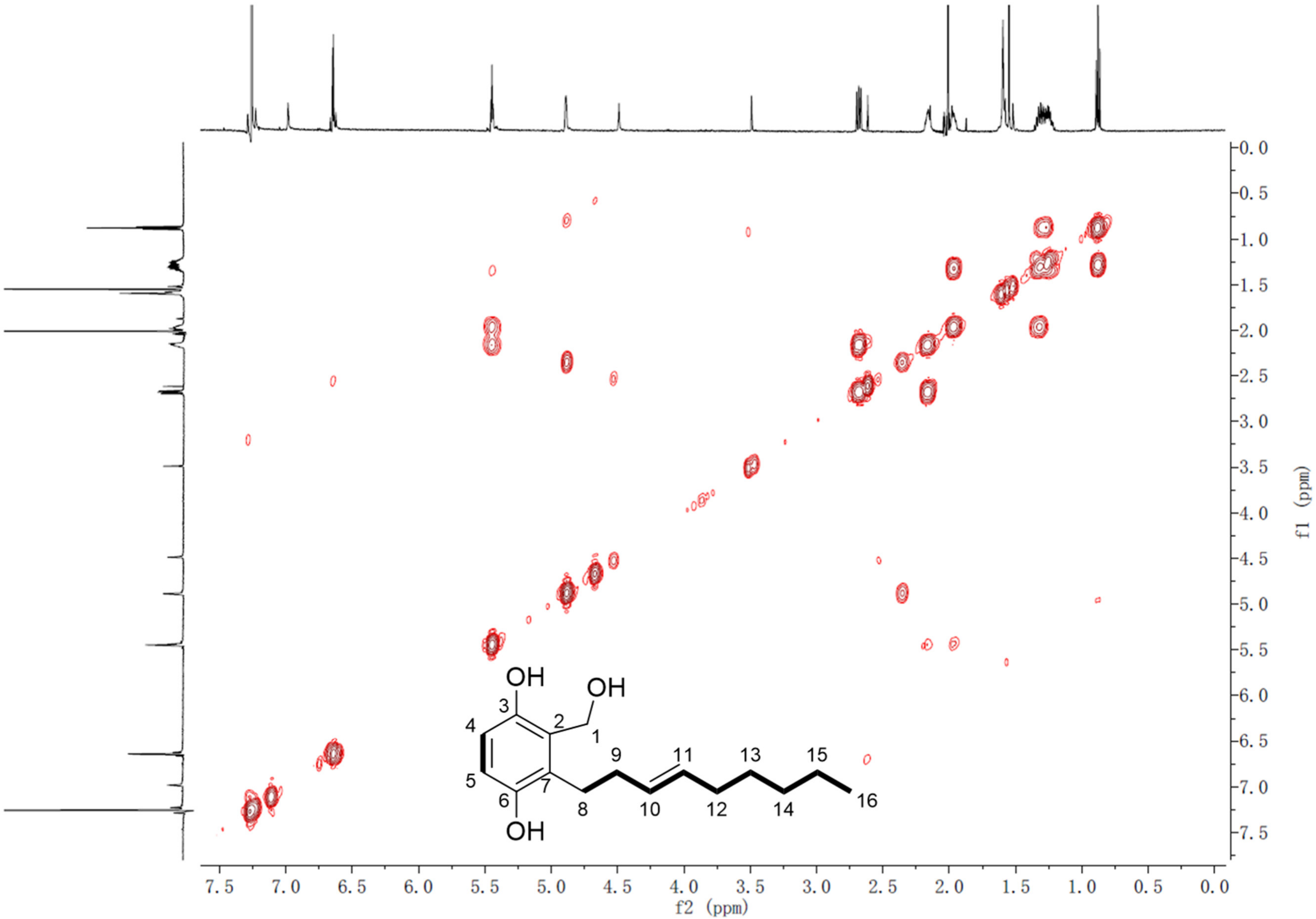

Figure S14. ^{1}H-^{1}H COSY spectrum of roquesalin A (**3**) in $CDCl_3$.

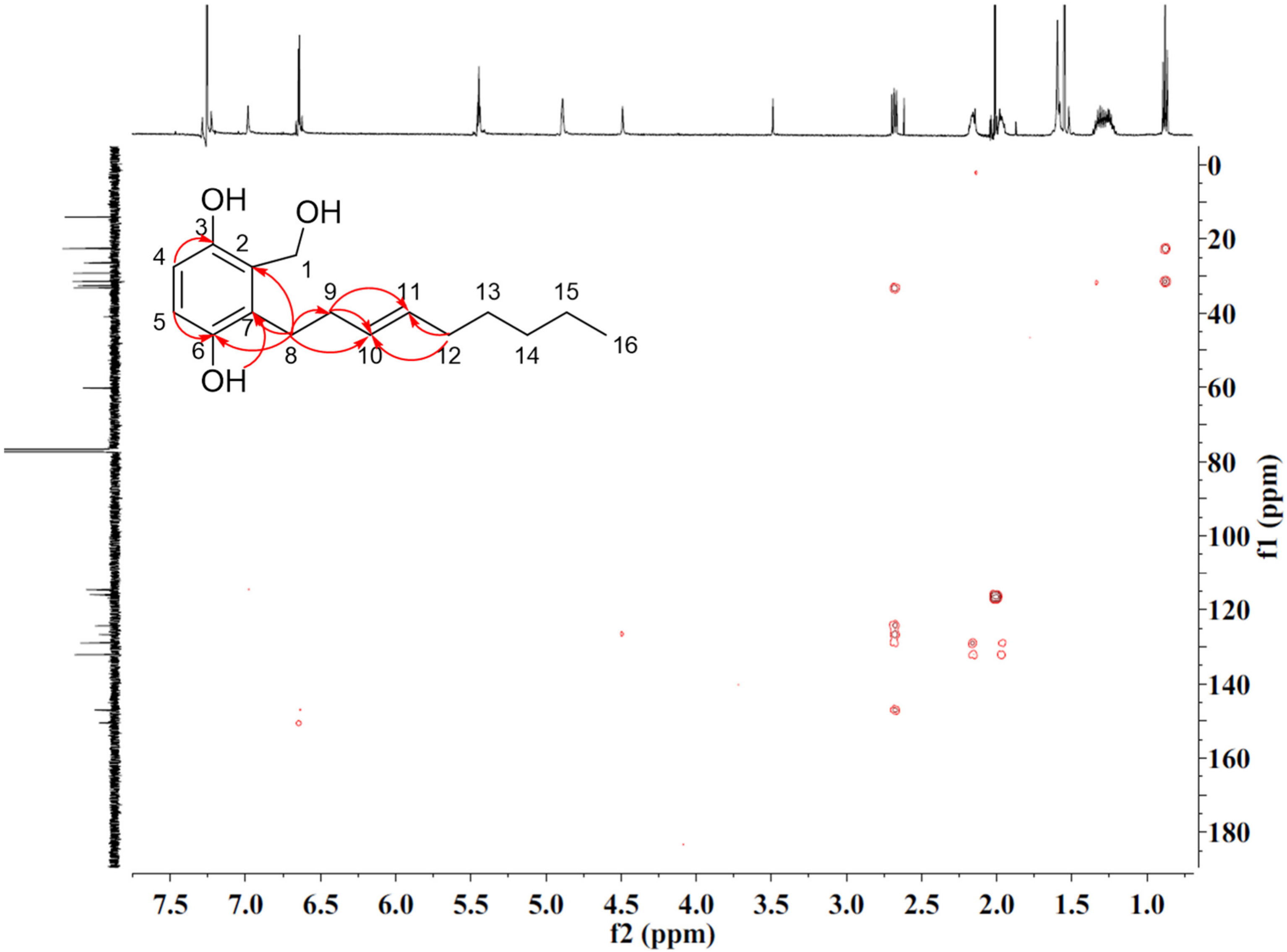

Figure S15. HMBC spectrum of roquesalin A (**3**) in $CDCl_3$.

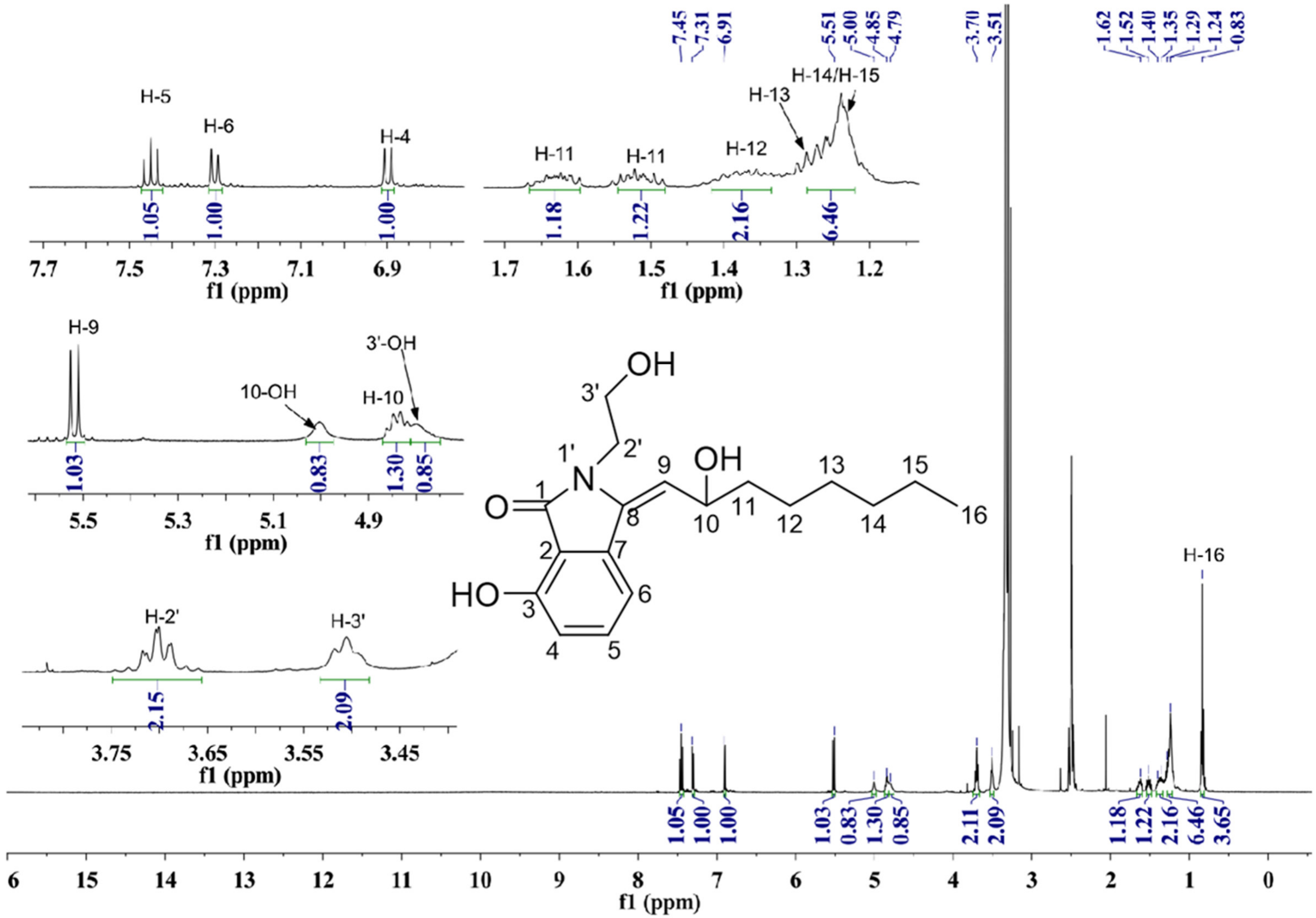

Figure S16. ^{1}H NMR spectrum of roquesalin B (**4**) in DMSO-d_6 (500 MHz).

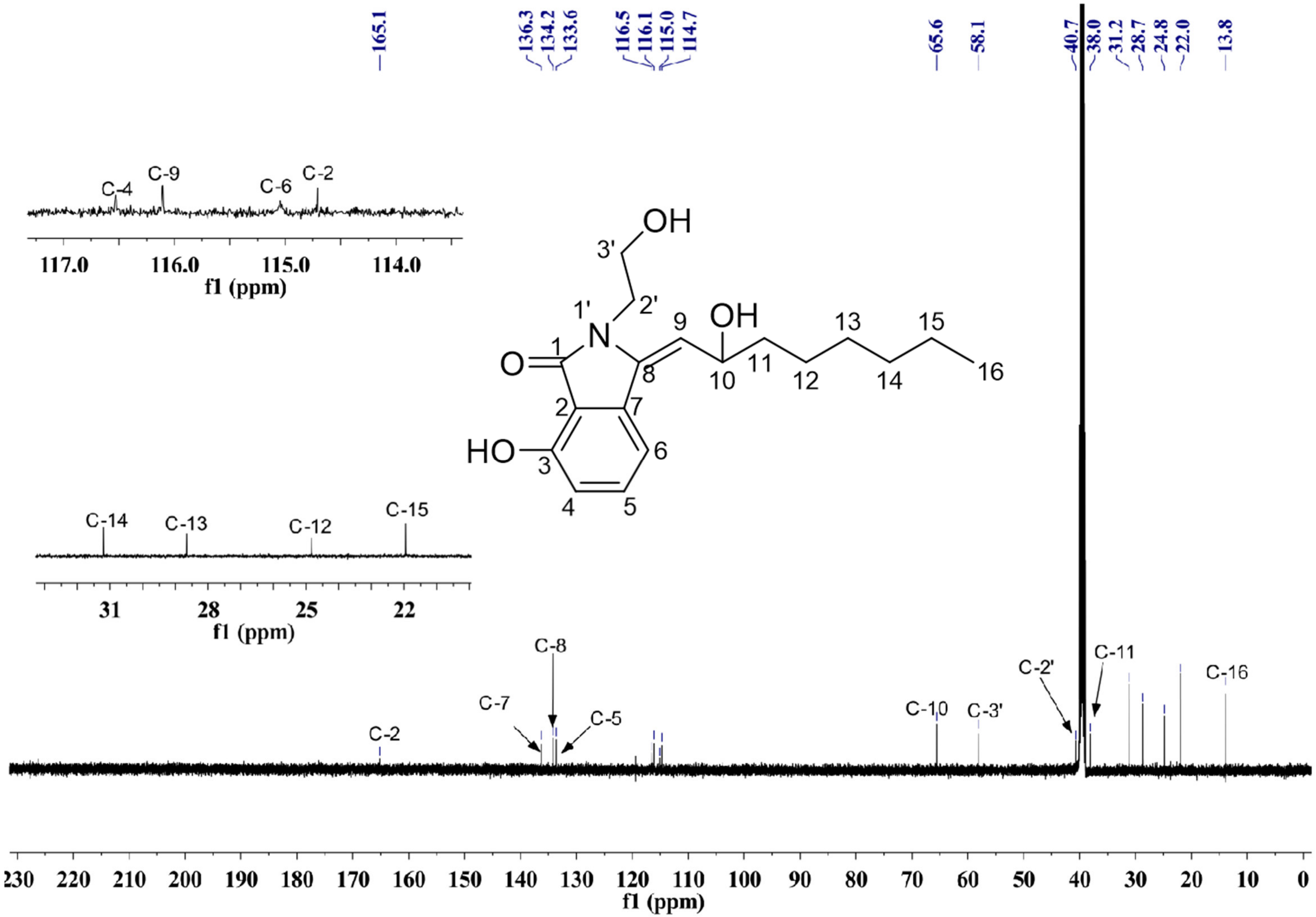

Figure S17. ^{13}C NMR spectrum of roquesalin B (**4**) in DMSO-d_6 (125 MHz).

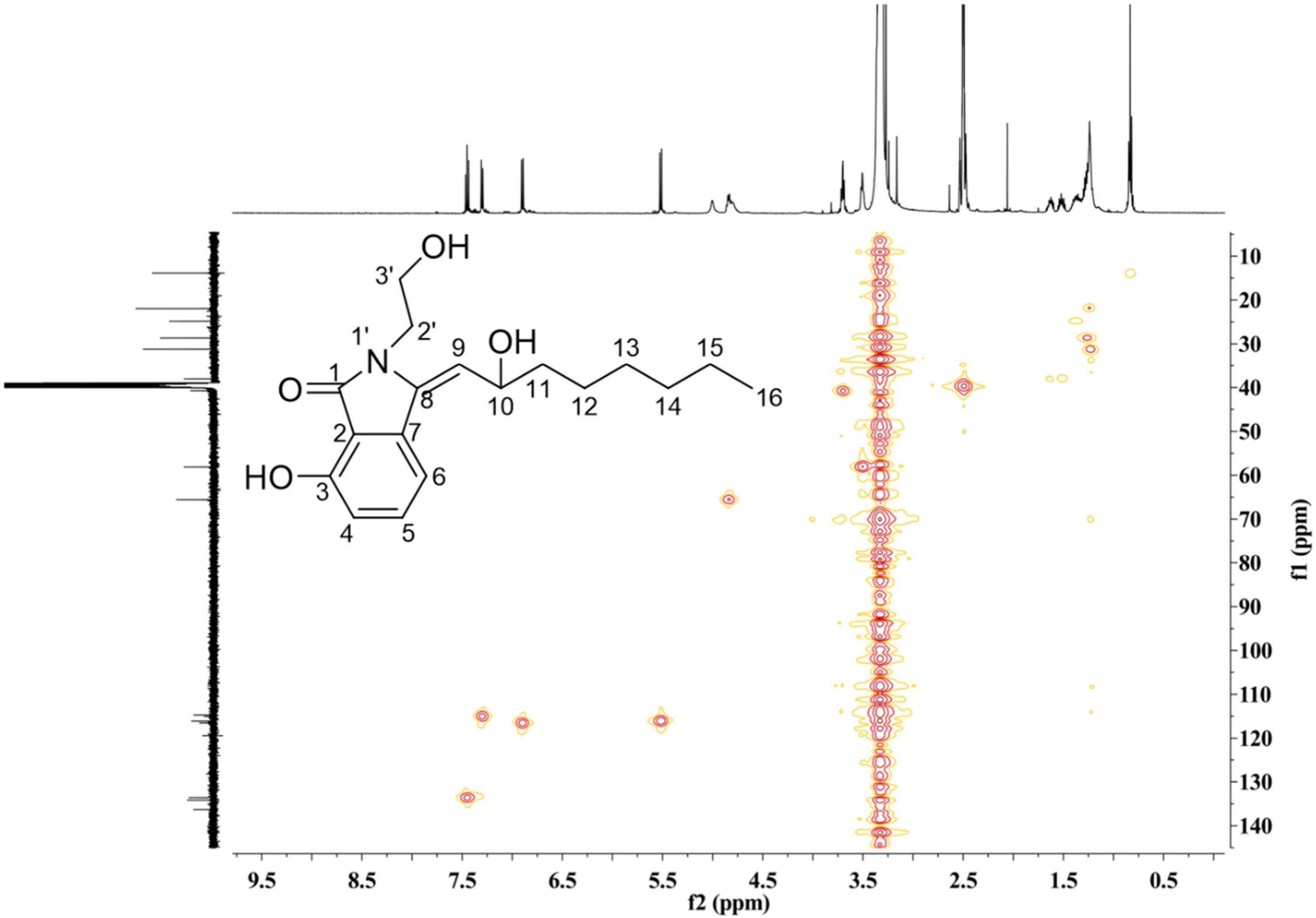

Figure S18. HSQC spectrum of roquesalin B (**4**) in DMSO-d_6.

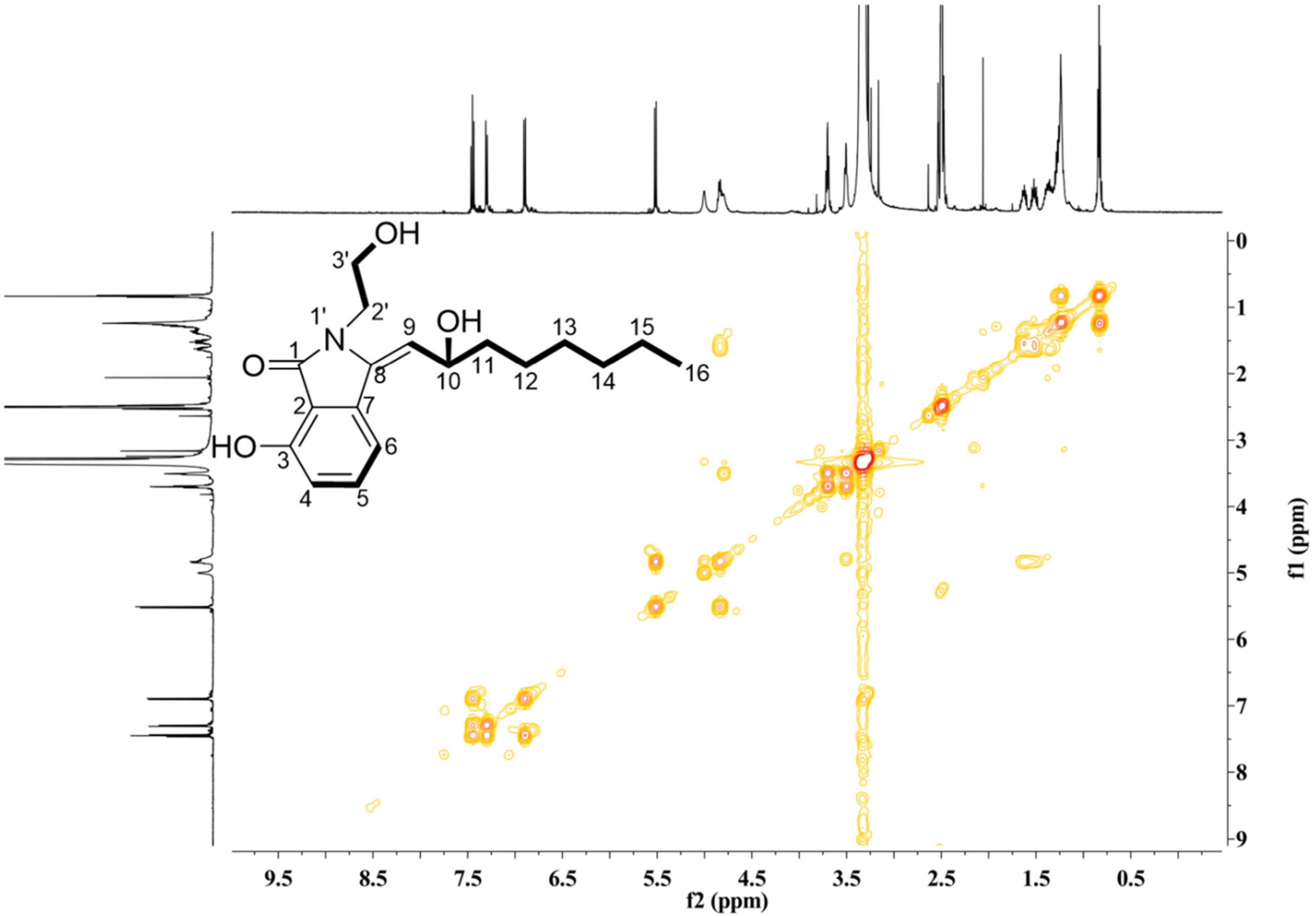

Figure S19. ^1H-^1H COSY spectrum of roquesalin B (**4**) in DMSO-d_6.

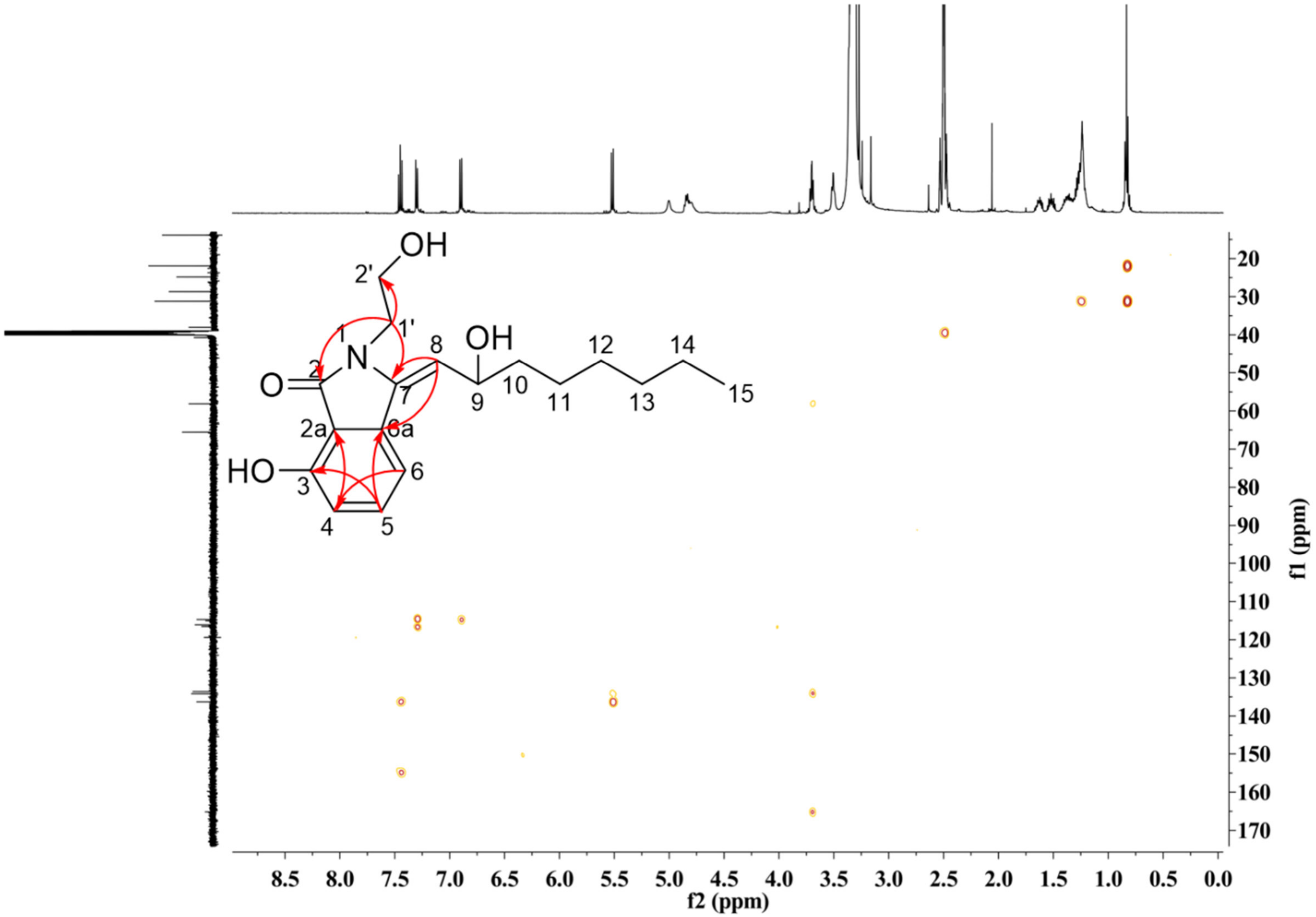

Figure S20. HMBC spectrum of roquesalin B (**4**) in DMSO-d_6.

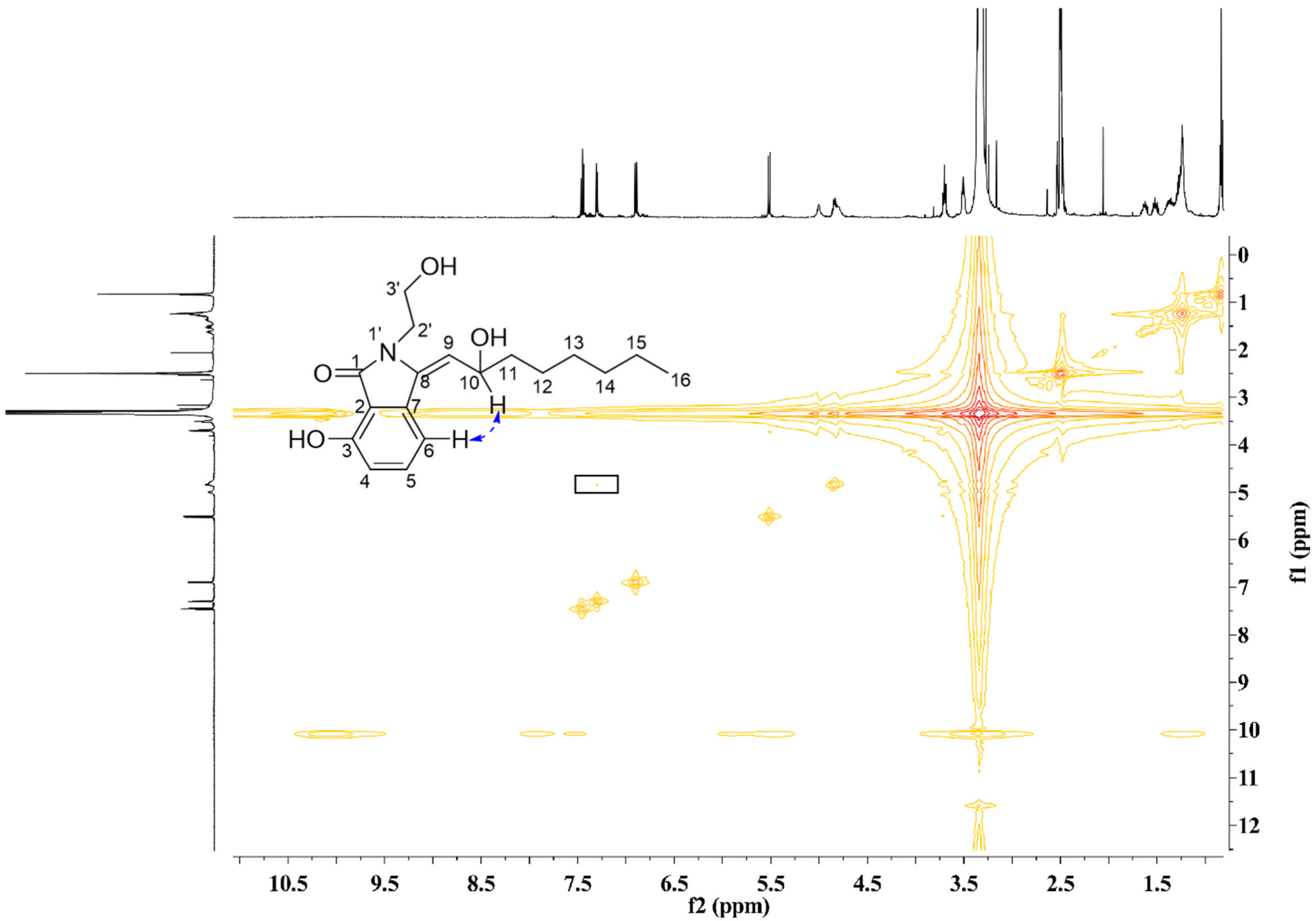

Figure S21. ^{1}H-^{1}H NOESY spectrum of roquesalin B (**4**) in DMSO-d_6.

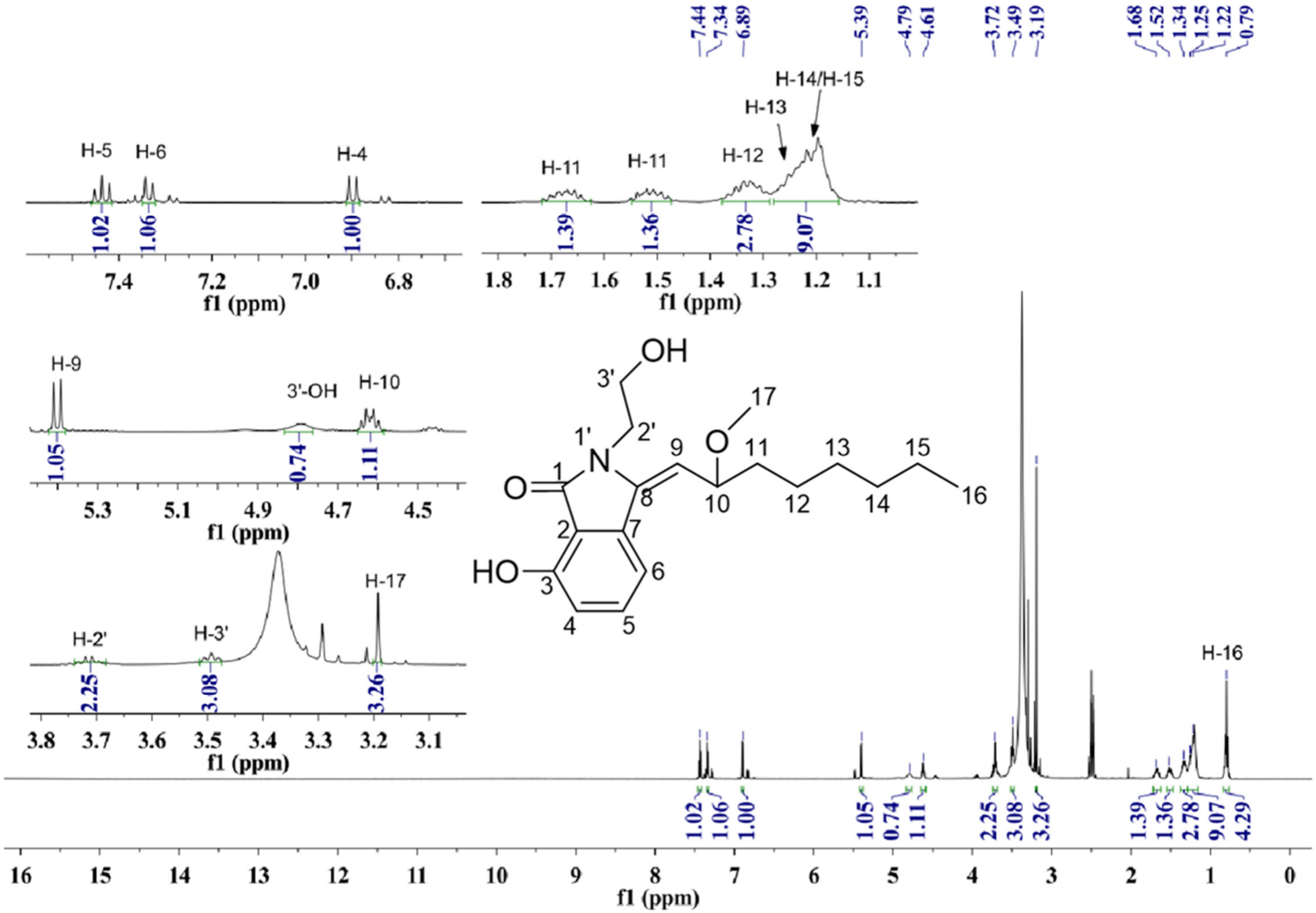

Figure S22. ^{1}H NMR spectrum of roquesalin C (**5**) in DMSO-d_6 (500 MHz).

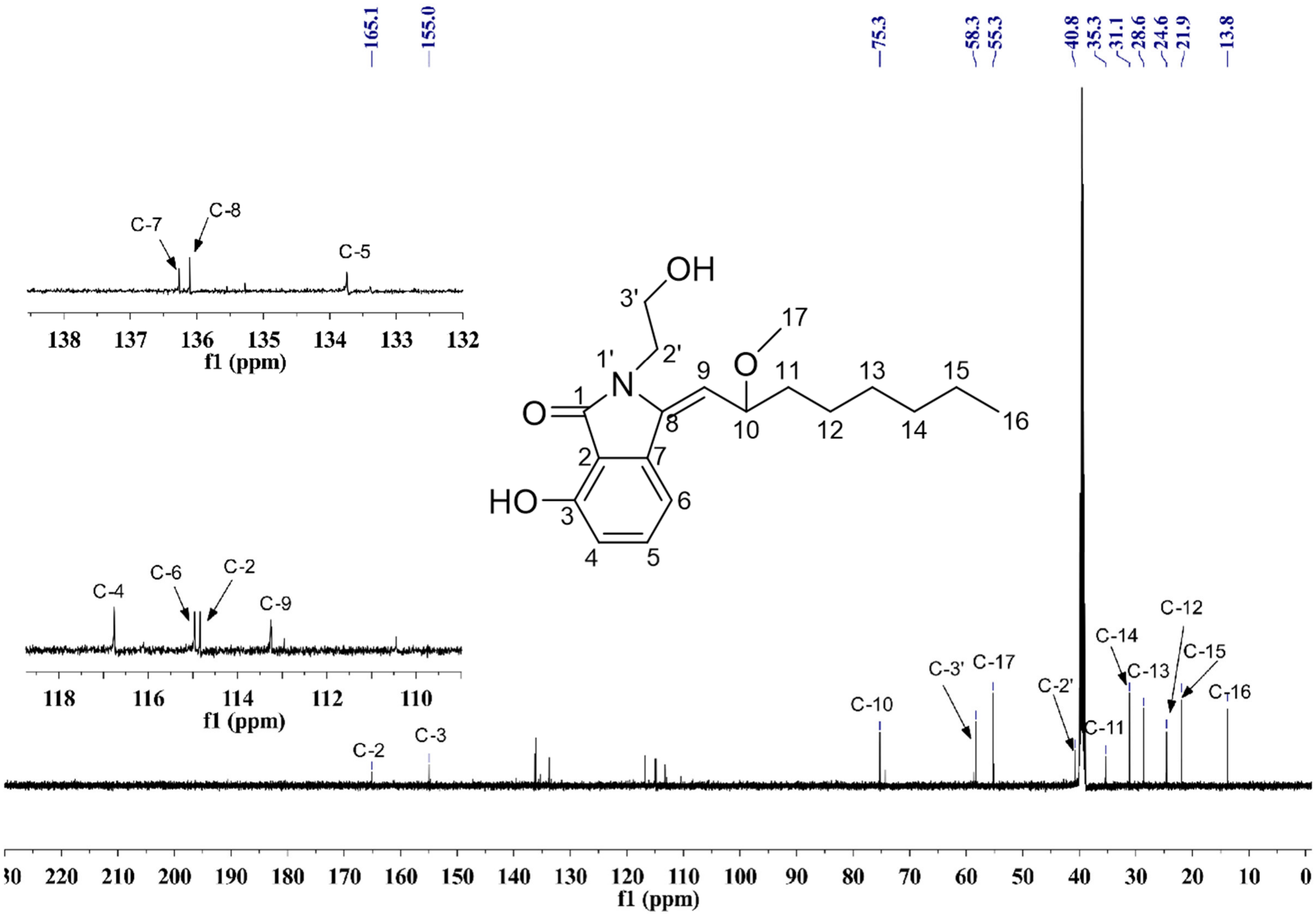

Figure S23. ^{13}C NMR spectrum of roquesalin C (**5**) in DMSO-d_6 (125 MHz).

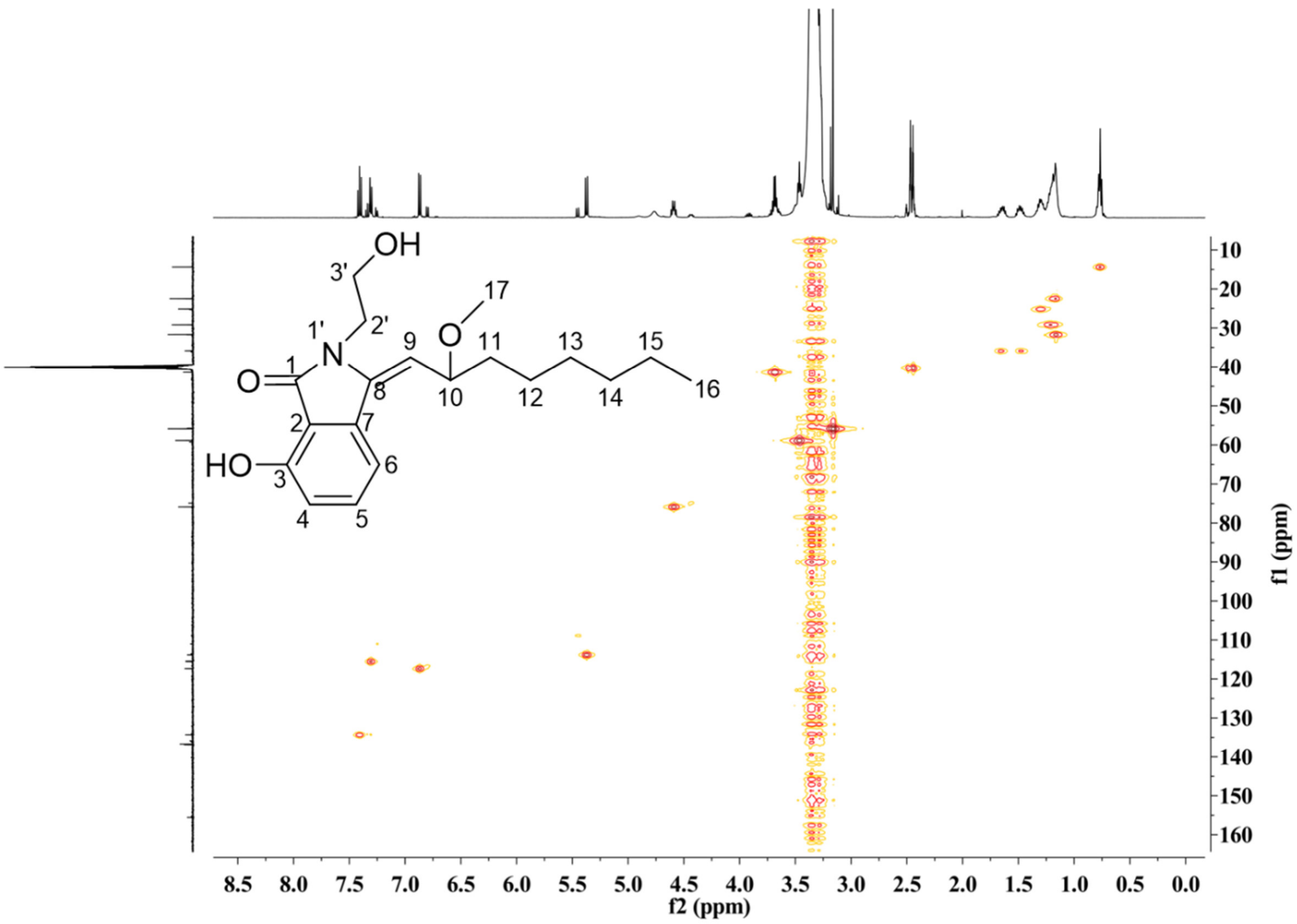

Figure S24. HSQC spectrum of roquesalin C (**5**) in DMSO-d_6.

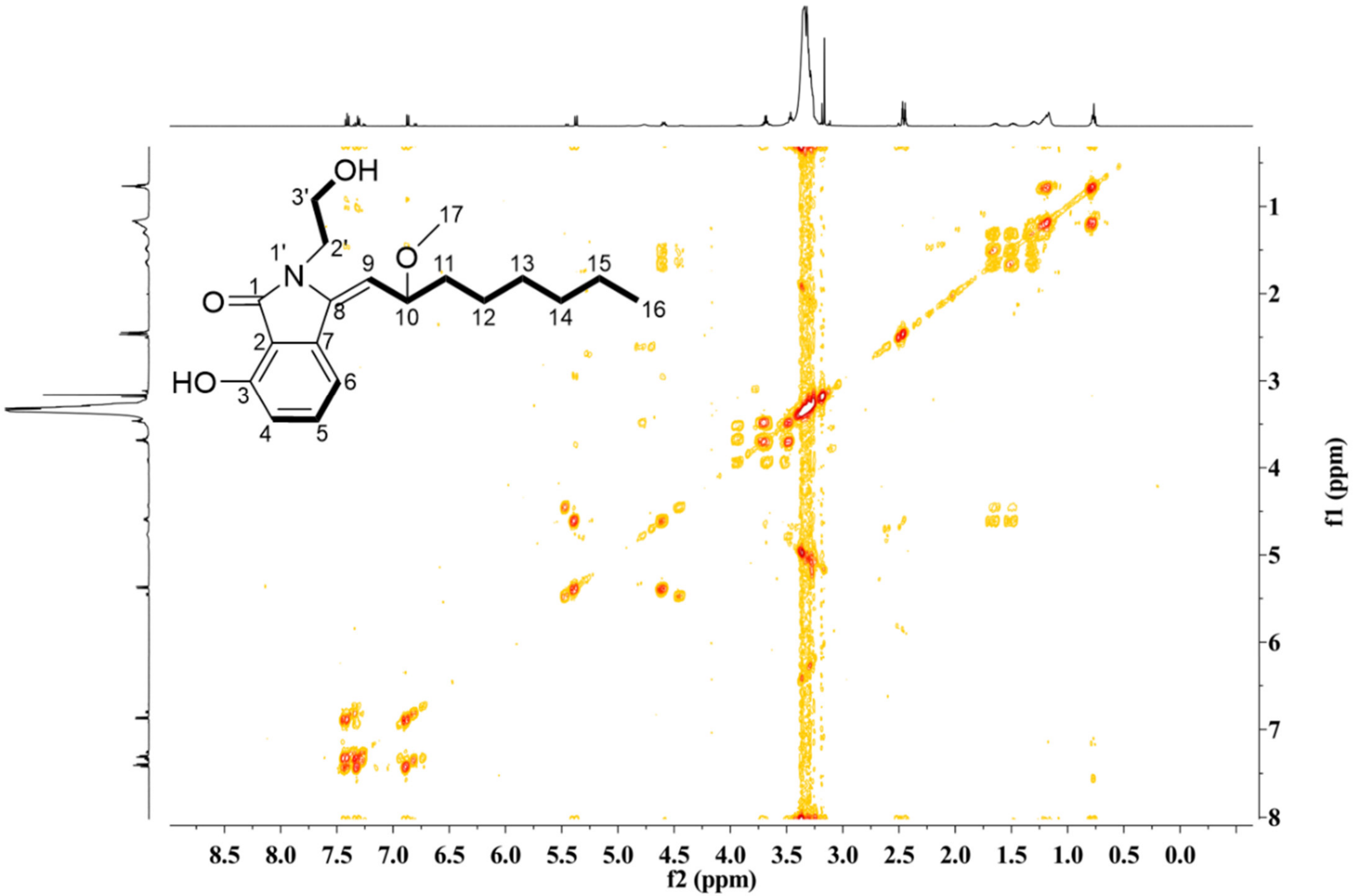

Figure S25. ^{1}H-^{1}H COSY spectrum of roquesalin C (**5**) in DMSO-d_6.

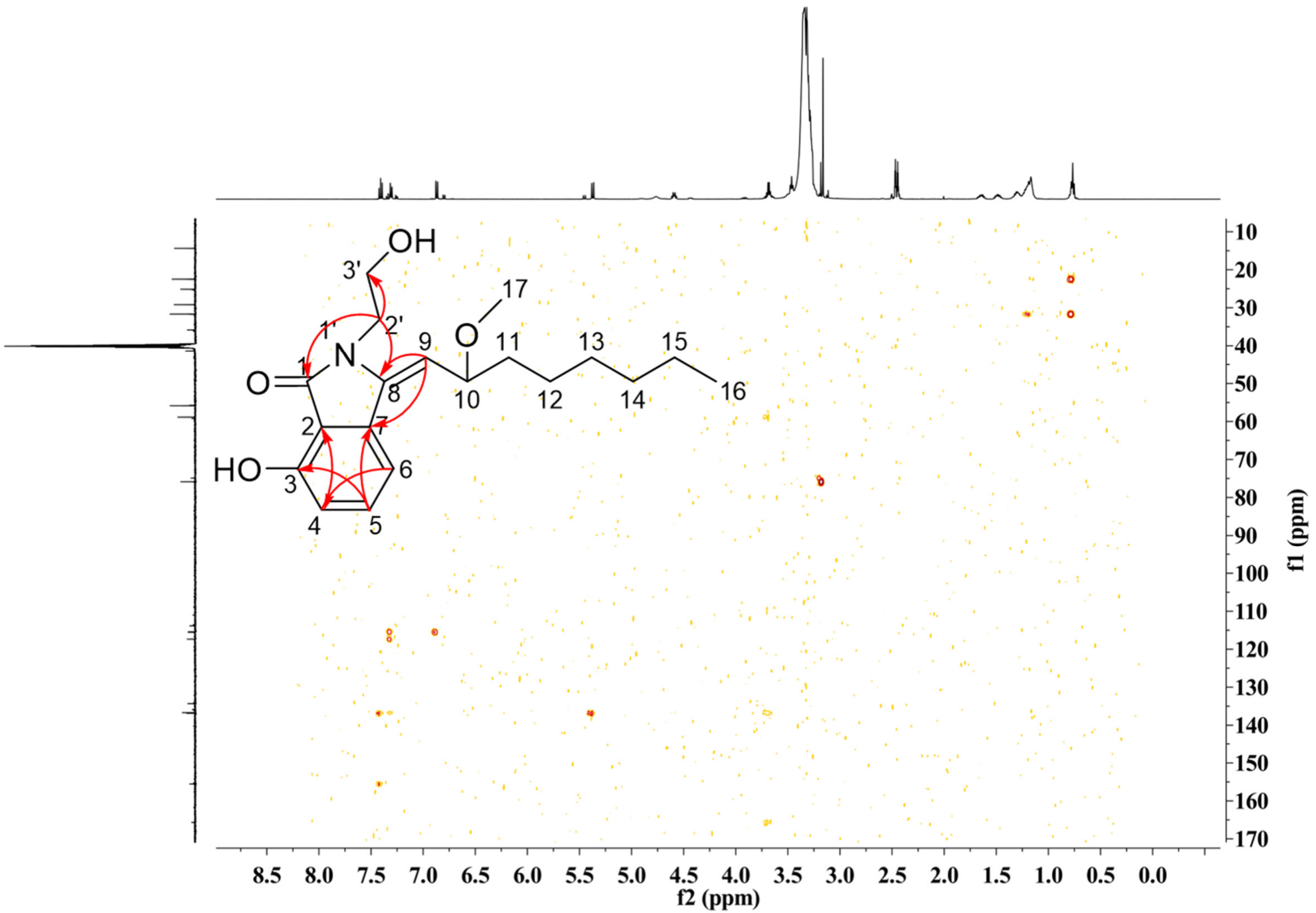

Figure S26. HMBC spectrum of roquesalin C (**5**) in DMSO-d_6.

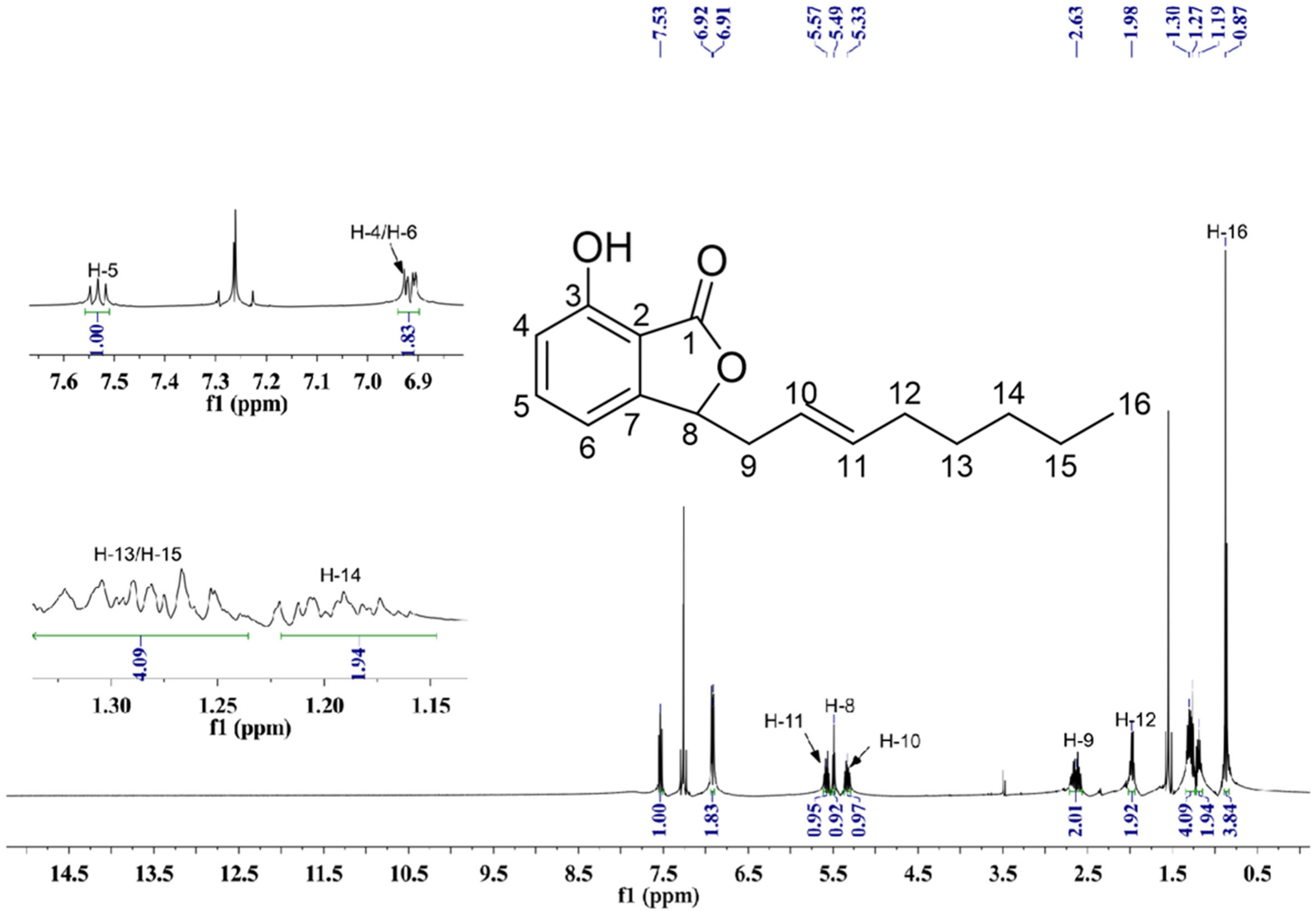

Figure S27. ^{1}H NMR spectrum of roquesalin D (**6**) in $CDCl_3$ (500 MHz).

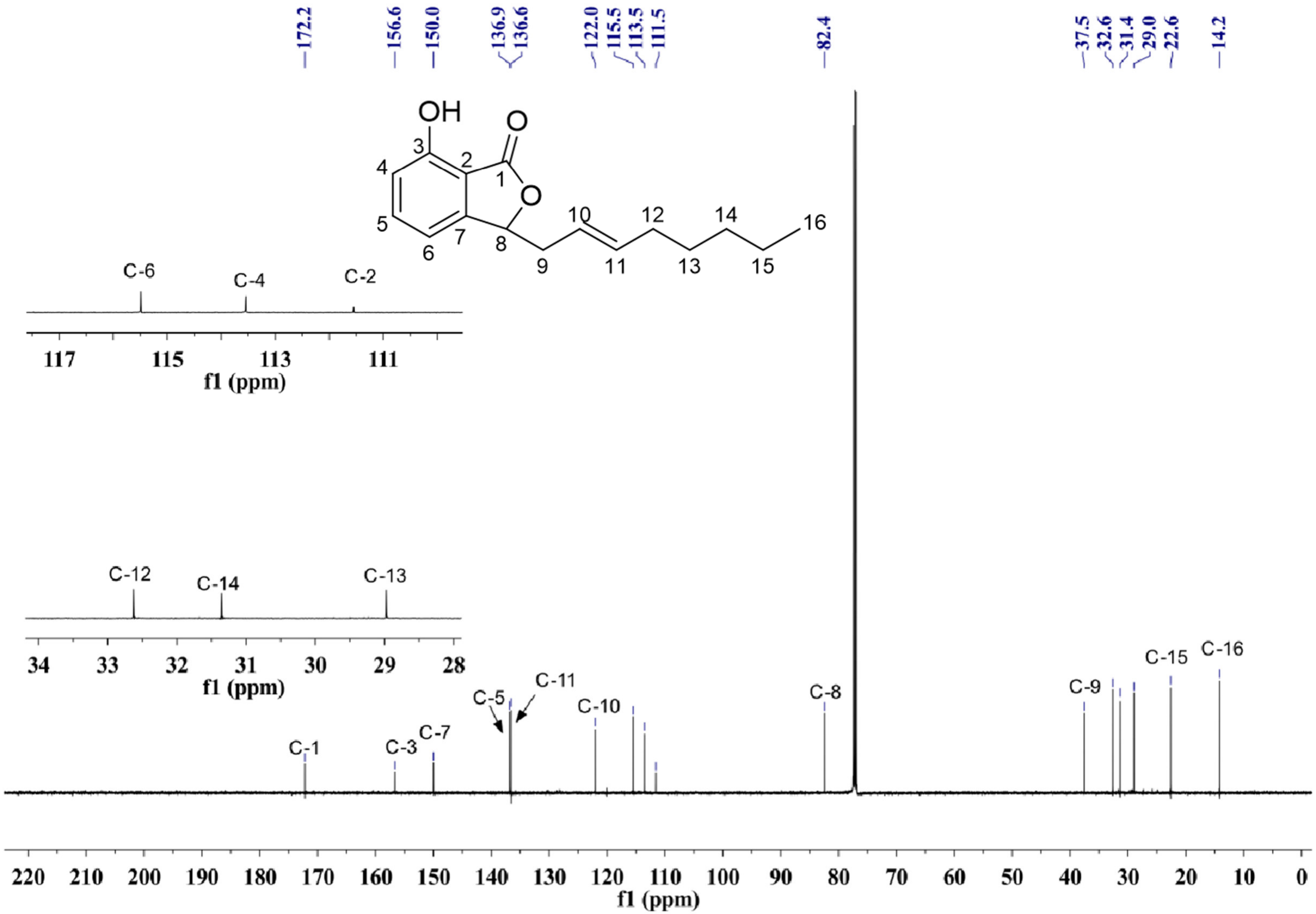

Figure S28. ^{13}C NMR spectrum of roquesalin D (**6**) in $CDCl_3$ (125 MHz).

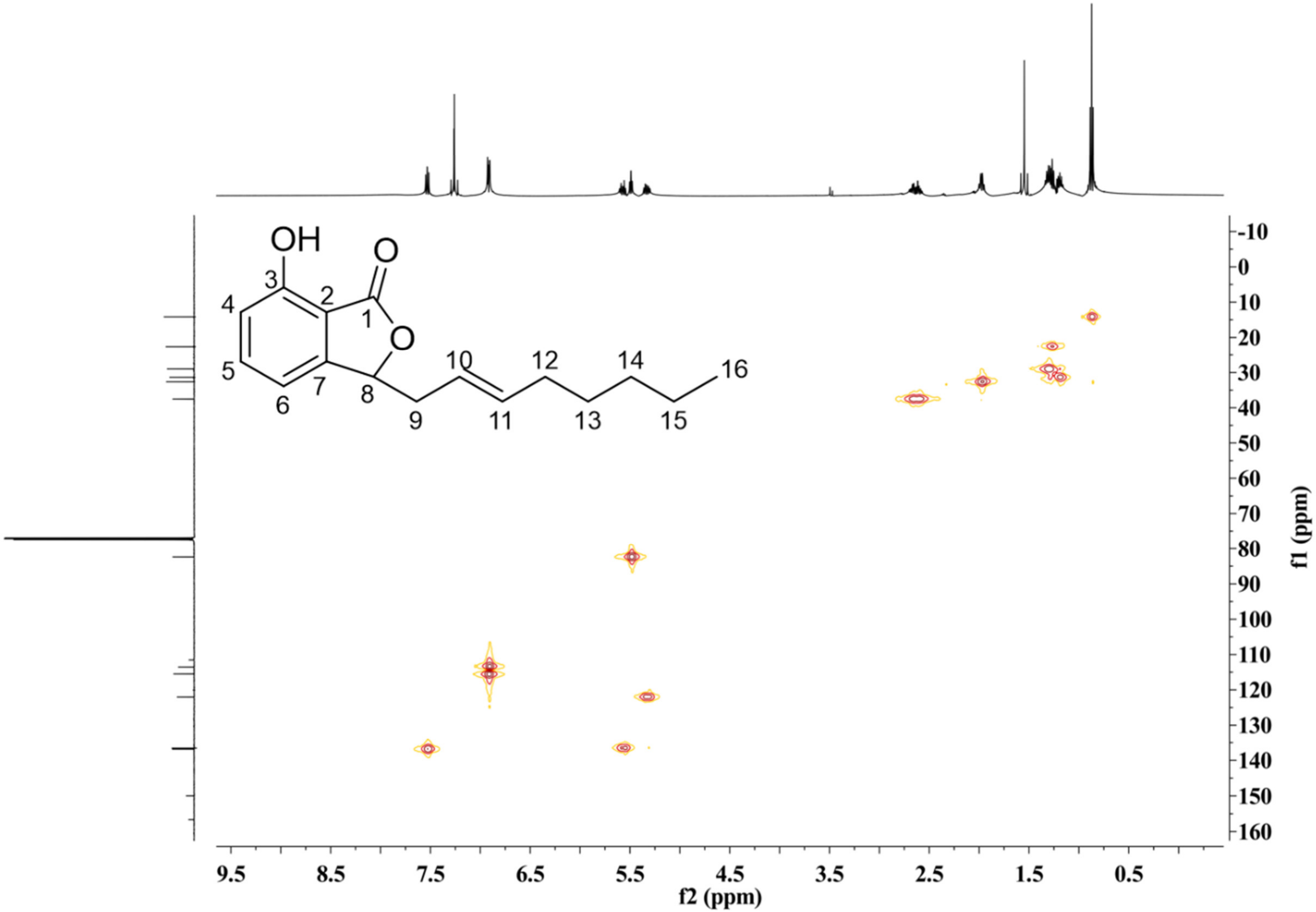

Figure S29. HSQC spectrum of roquesalin D (**6**) in $CDCl_3$.

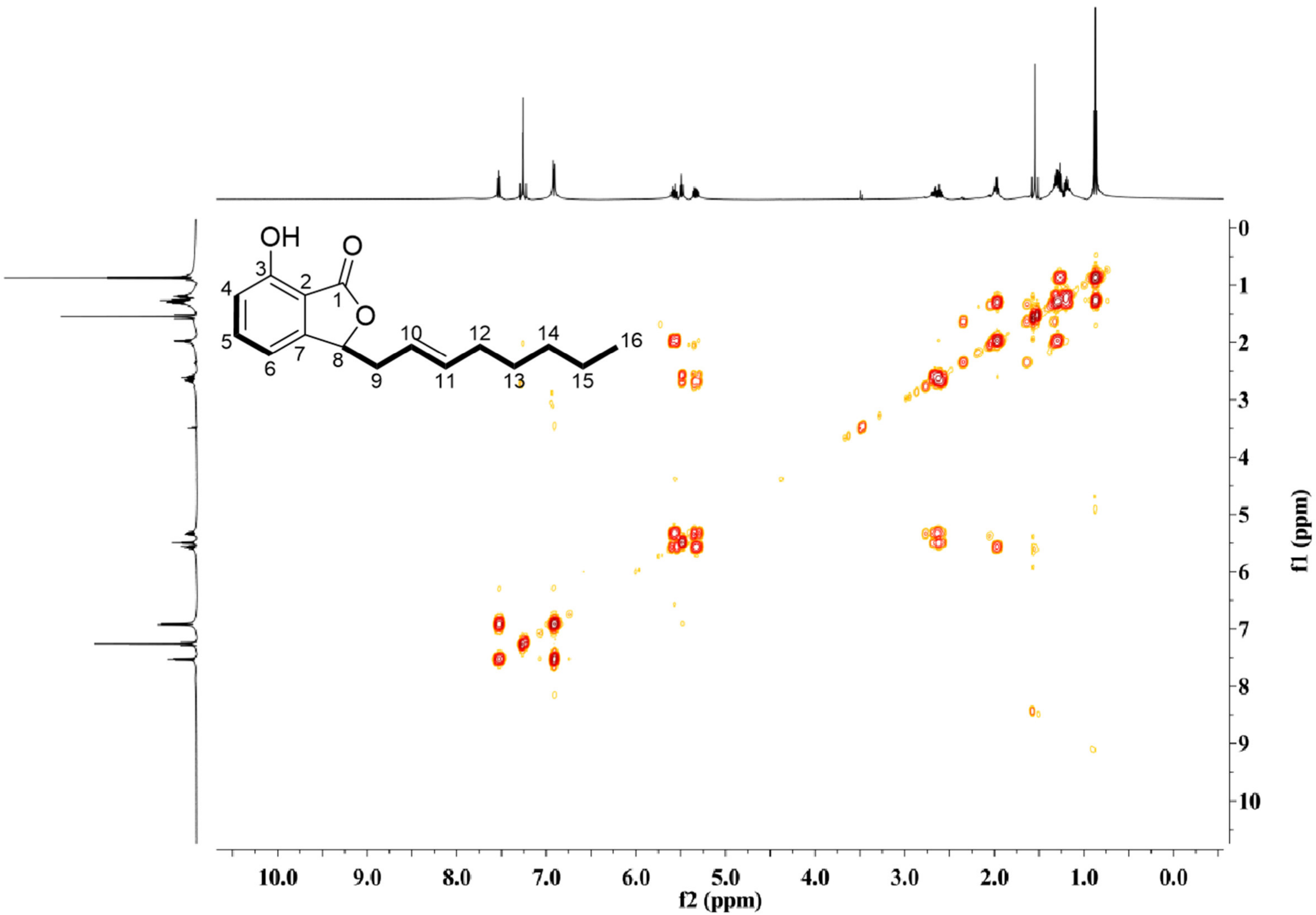

Figure S30. ${}^{1}H$-${}^{1}H$ COSY spectrum of roquesalin D (**6**) in $CDCl_3$.

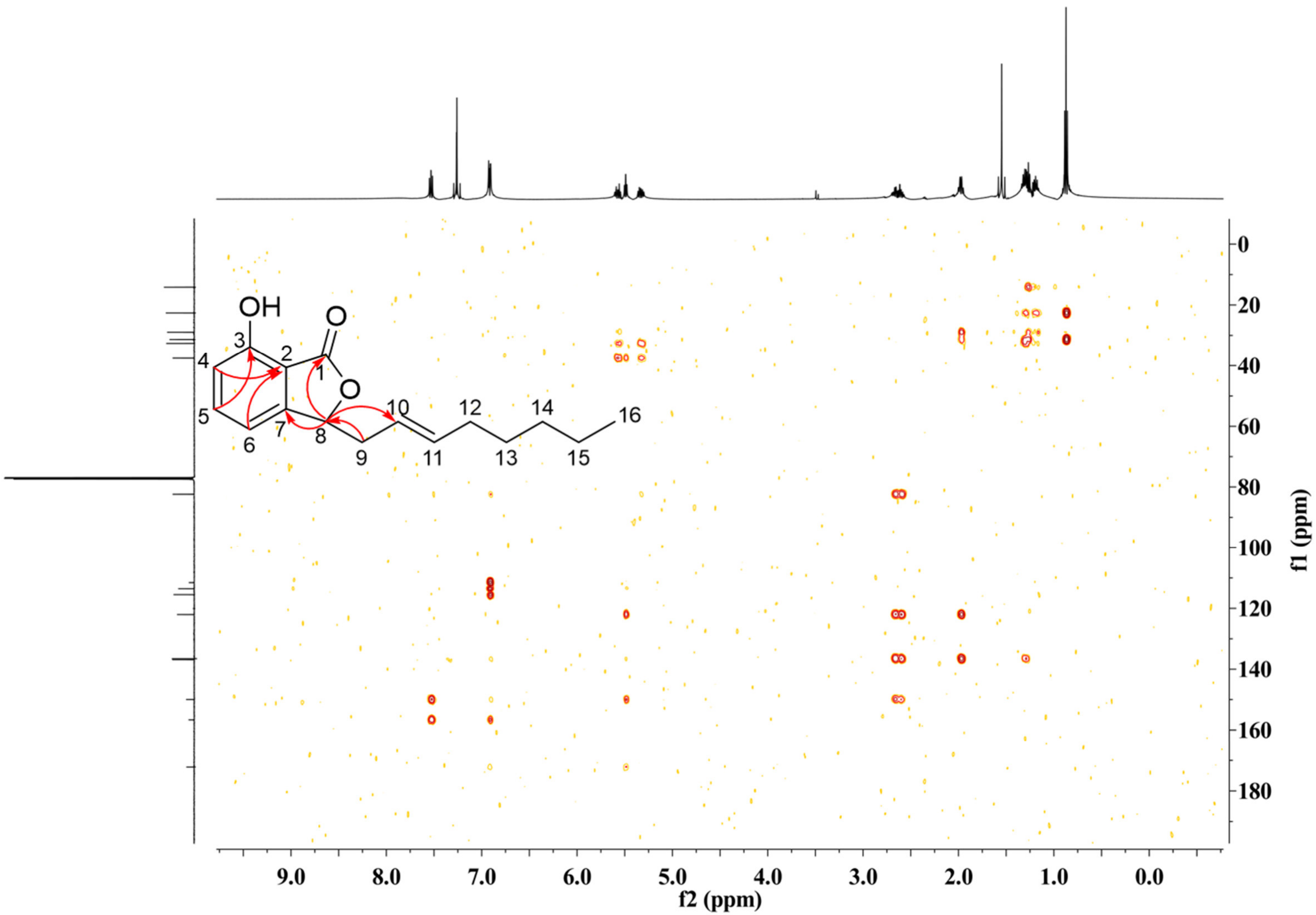

Figure S31. HMBC spectrum of roquesalin D (**6**) in $CDCl_3$.

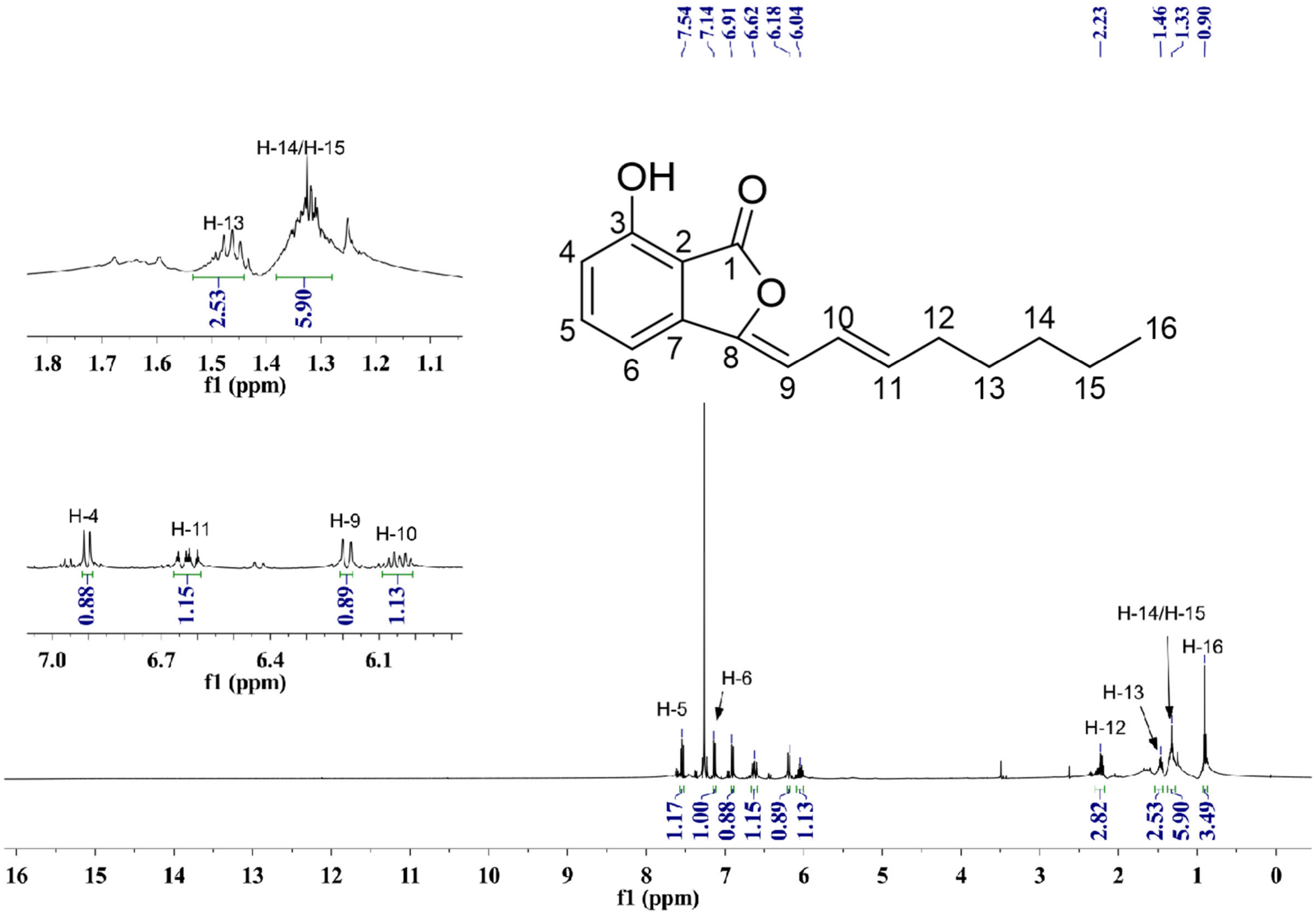

Figure S32. ^{1}H NMR spectrum of roquesalin E (**7**) in $CDCl_3$ (500 MHz).

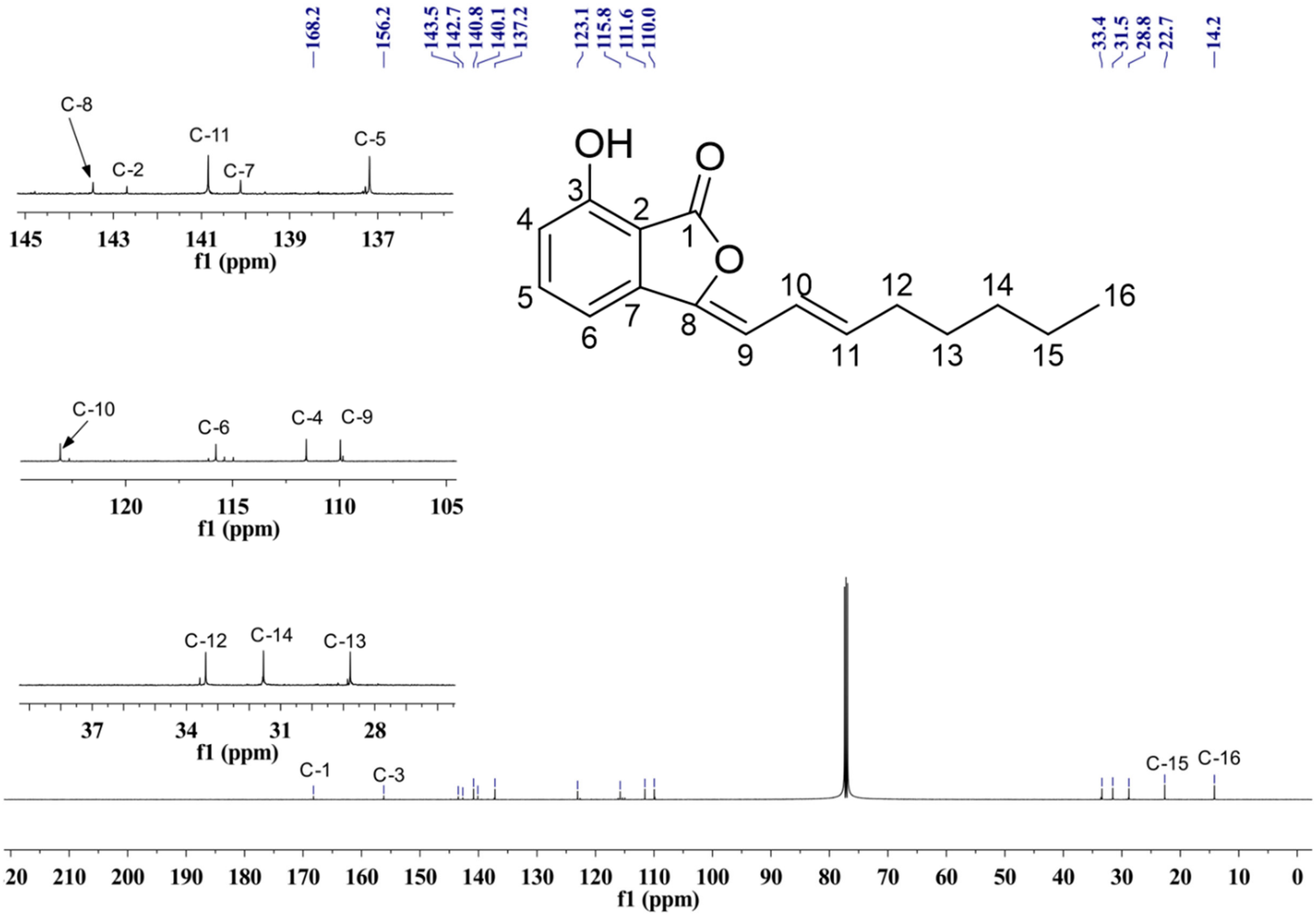

Figure S33. ^{13}C NMR spectrum of roquesalin E (**7**) in $CDCl_3$ (125 MHz).

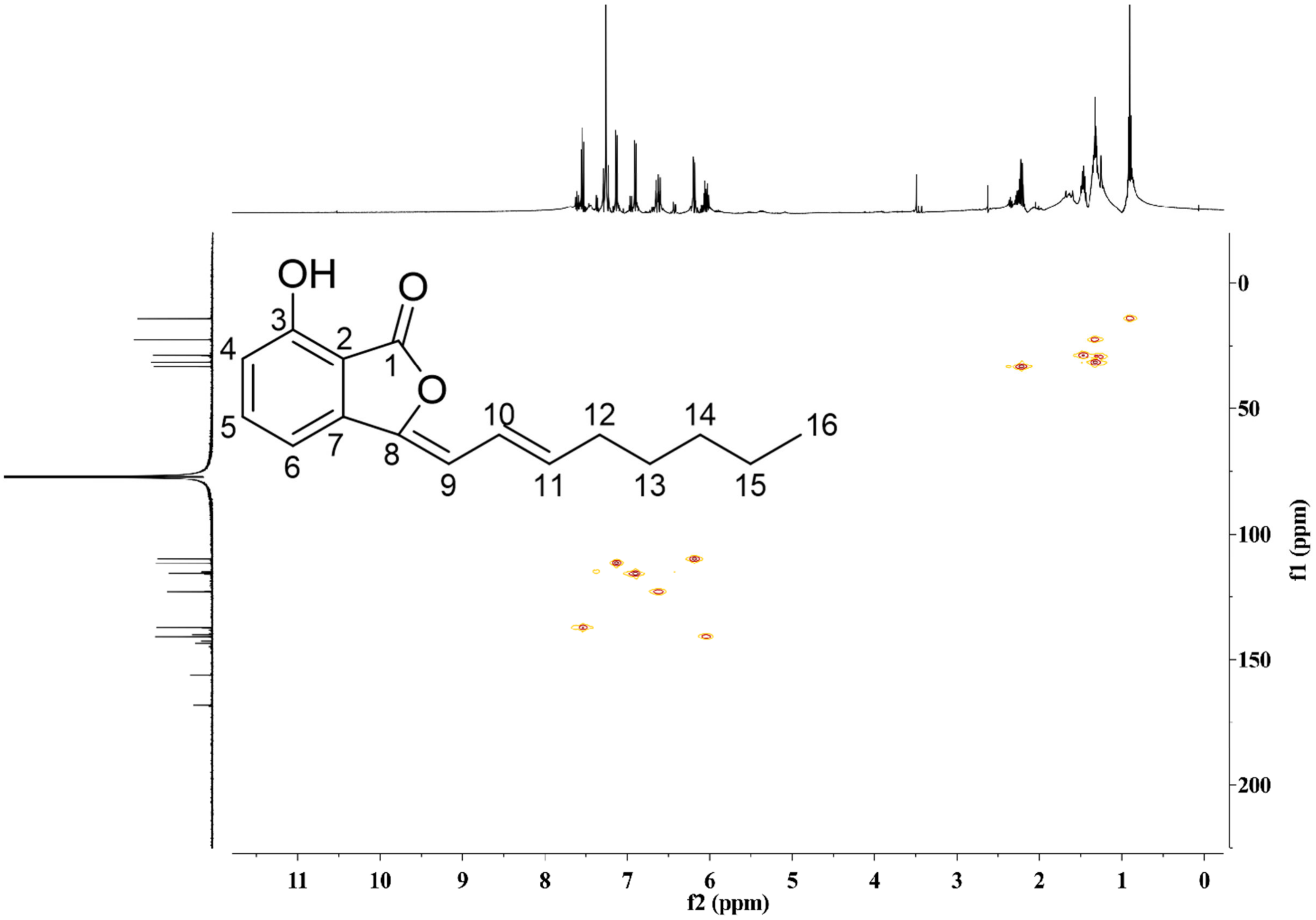

Figure S34. HSQC spectrum of roquesalin E (**7**) in $CDCl_3$.

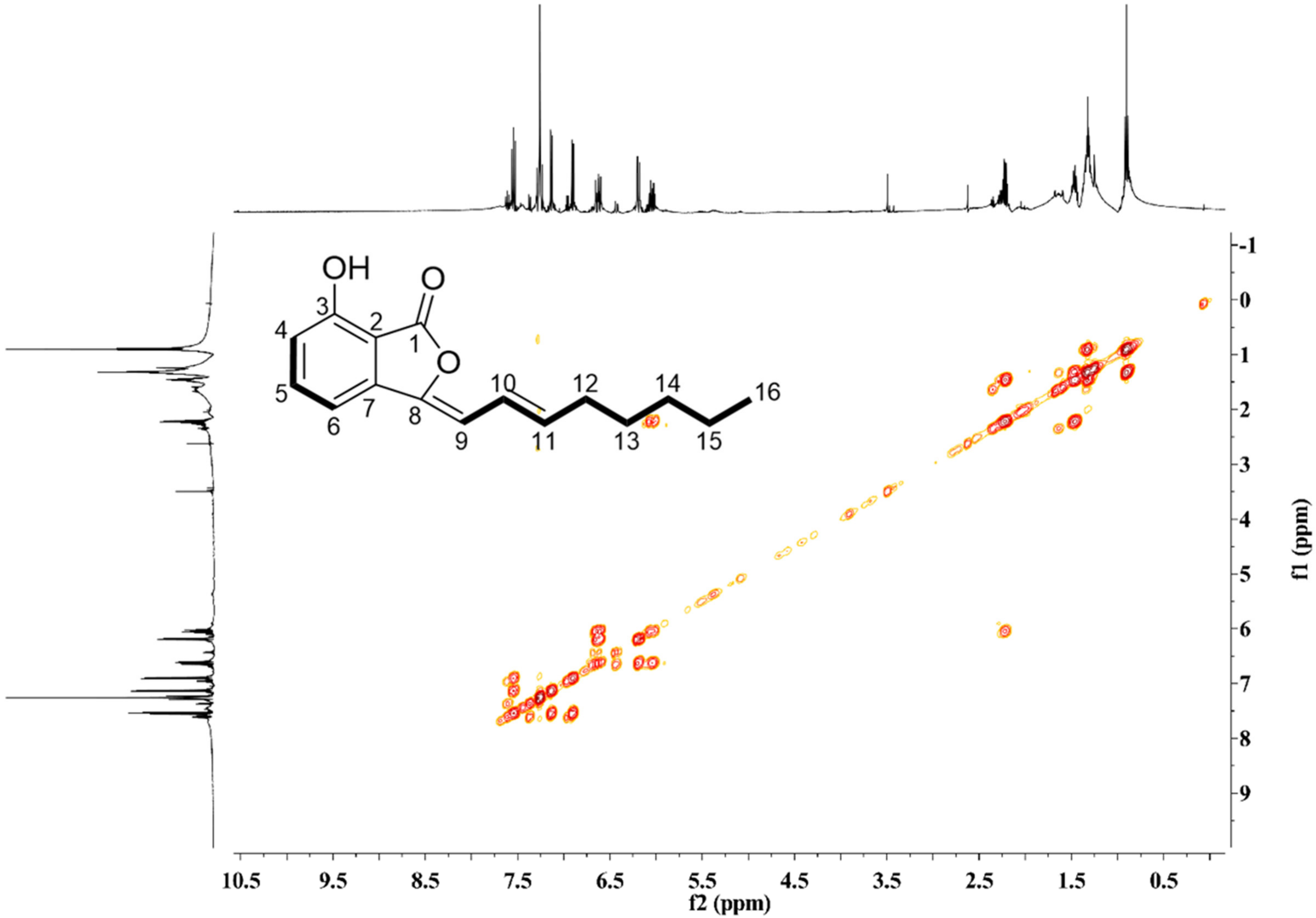

Figure S35. ¹H-¹H COSY spectrum of roquesalin E (**7**) in $CDCl_3$.

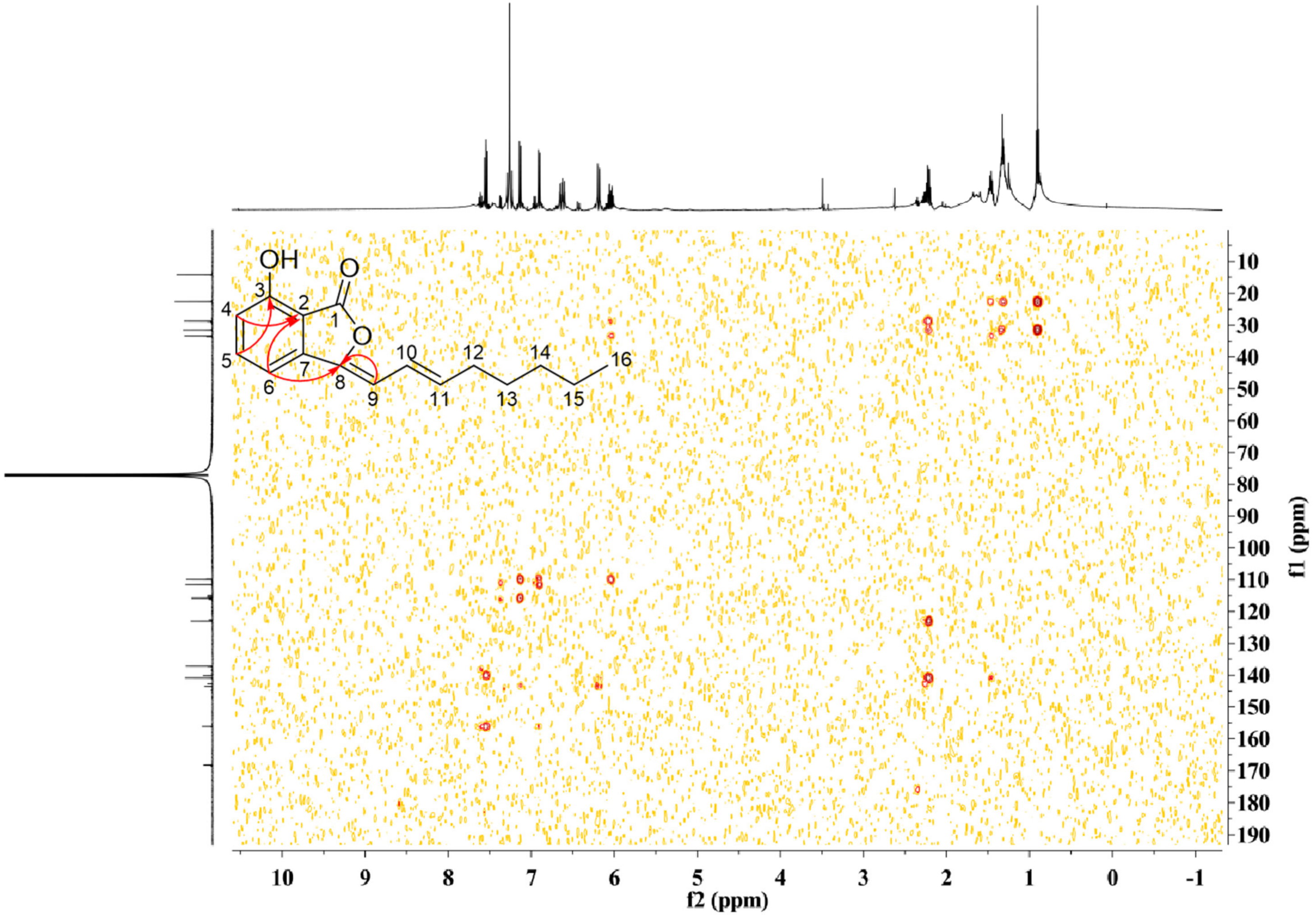

Figure S36. HMBC spectrum of roquesalin E (**7**) in $CDCl_3$.

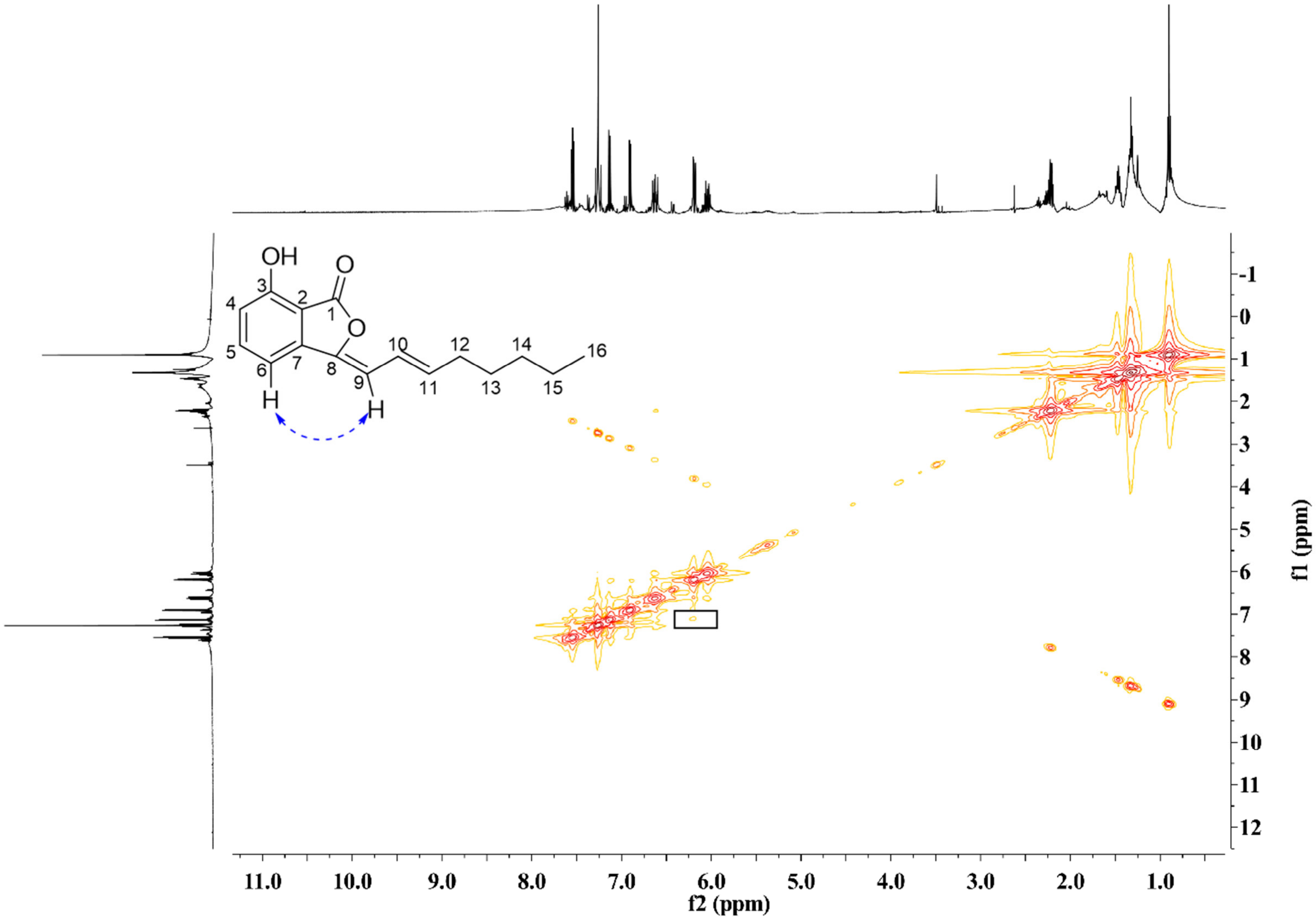

Figure S37. ^1H-^1H NOESY spectrum of roquesalin E (**7**) in $CDCl_3$.

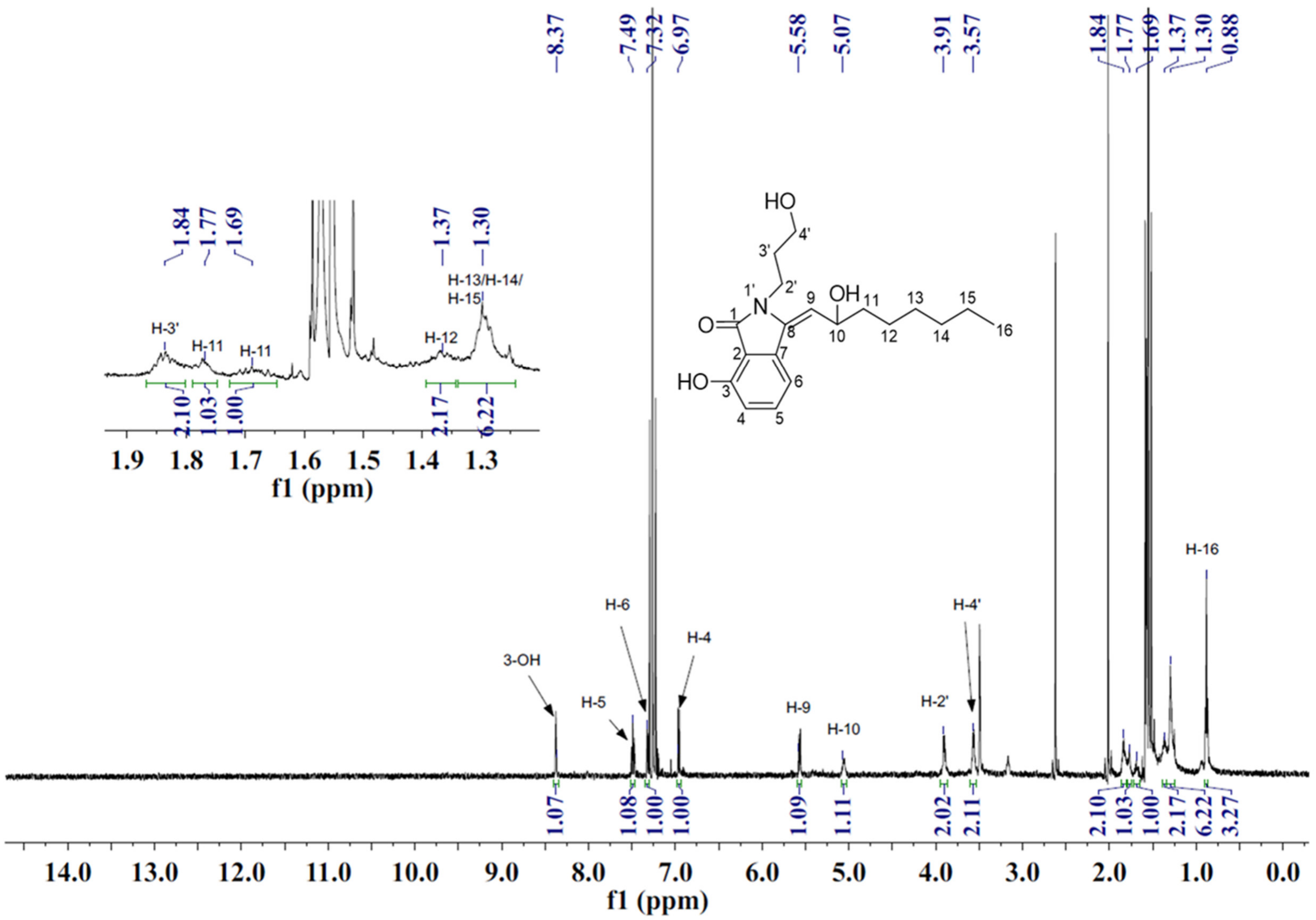

Figure S38. ^{1}H NMR spectrum of roquesalin F (**8**) in $CDCl_3$ (500 MHz).

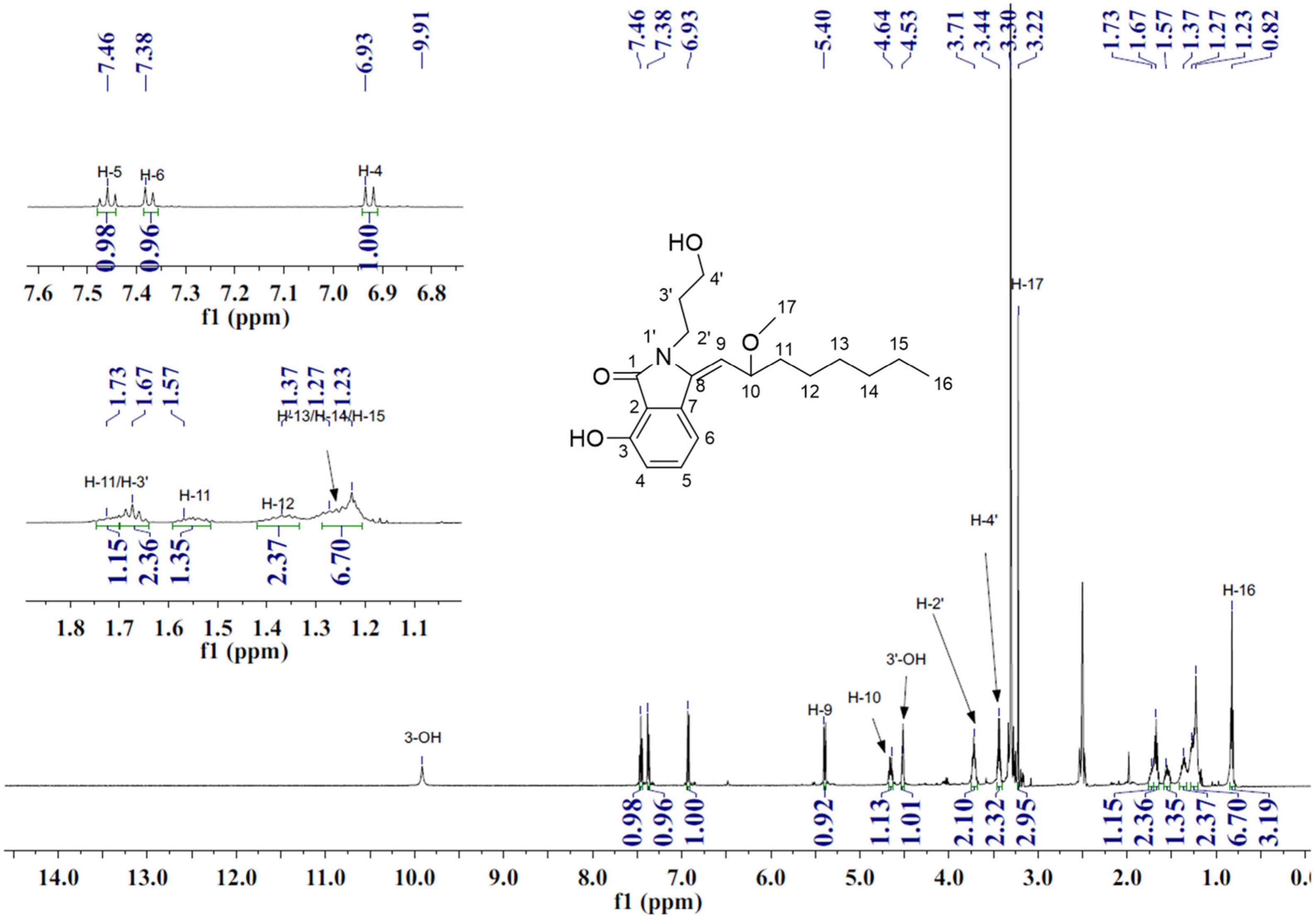

Figure S39. ^{1}H NMR spectrum of roquesalin G (**9**) in DMSO-d_6 (500 MHz).

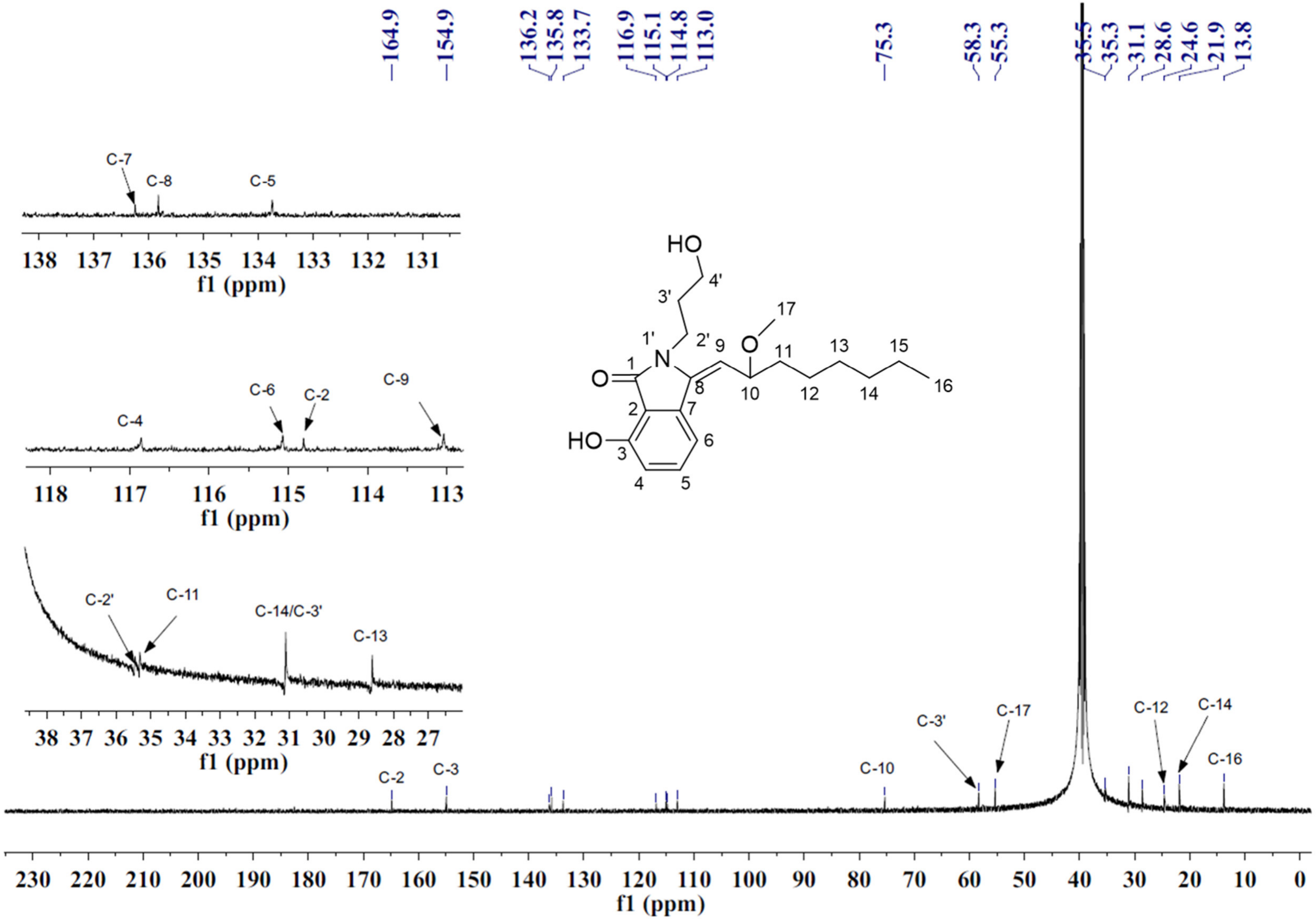

Figure S40. ^{13}C NMR spectrum of roquesalin G (**9**) in DMSO-d_6 (125 MHz).

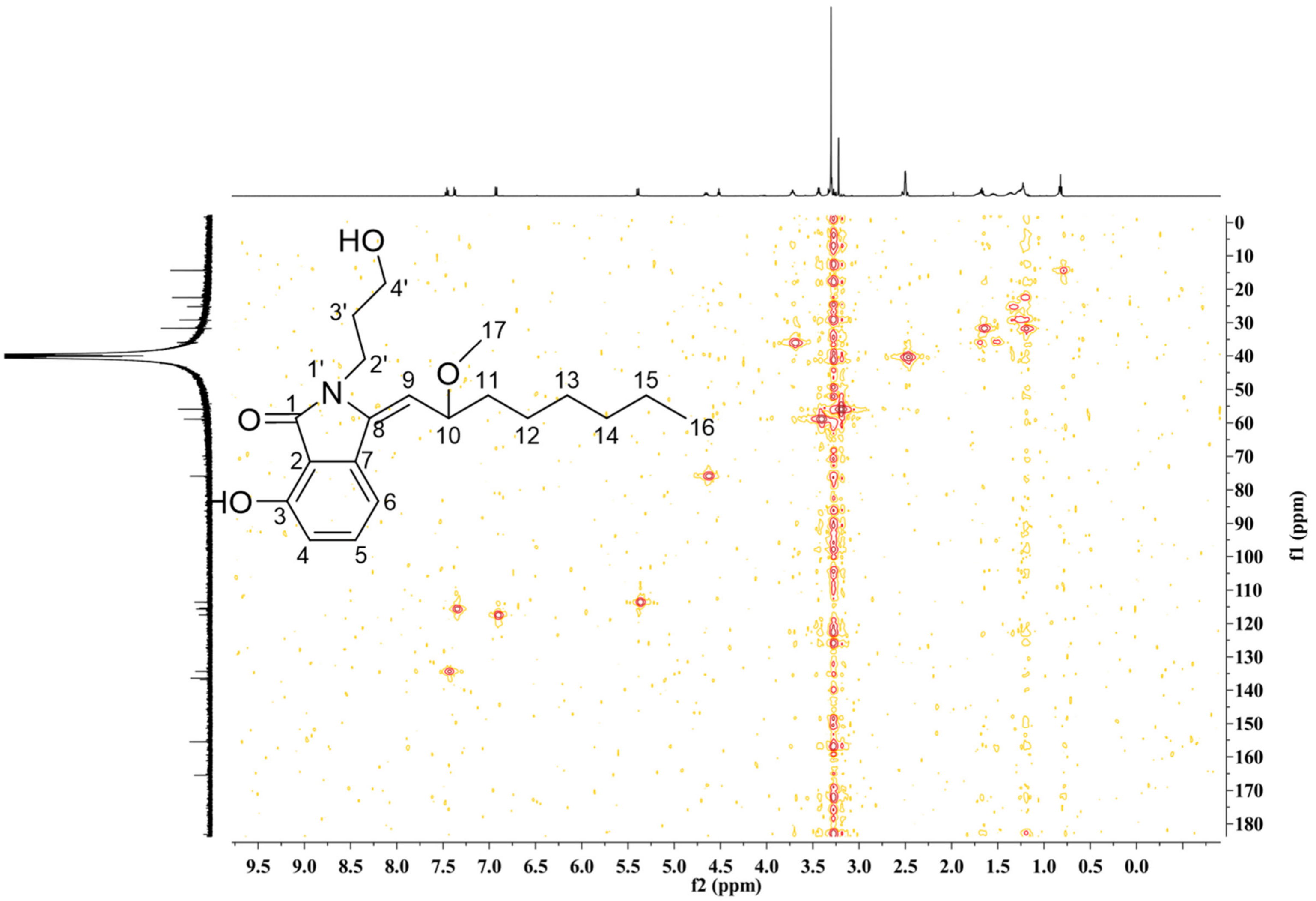

Figure S41. HSQC spectrum of roquesalin G (**9**) in DMSO-d_6.

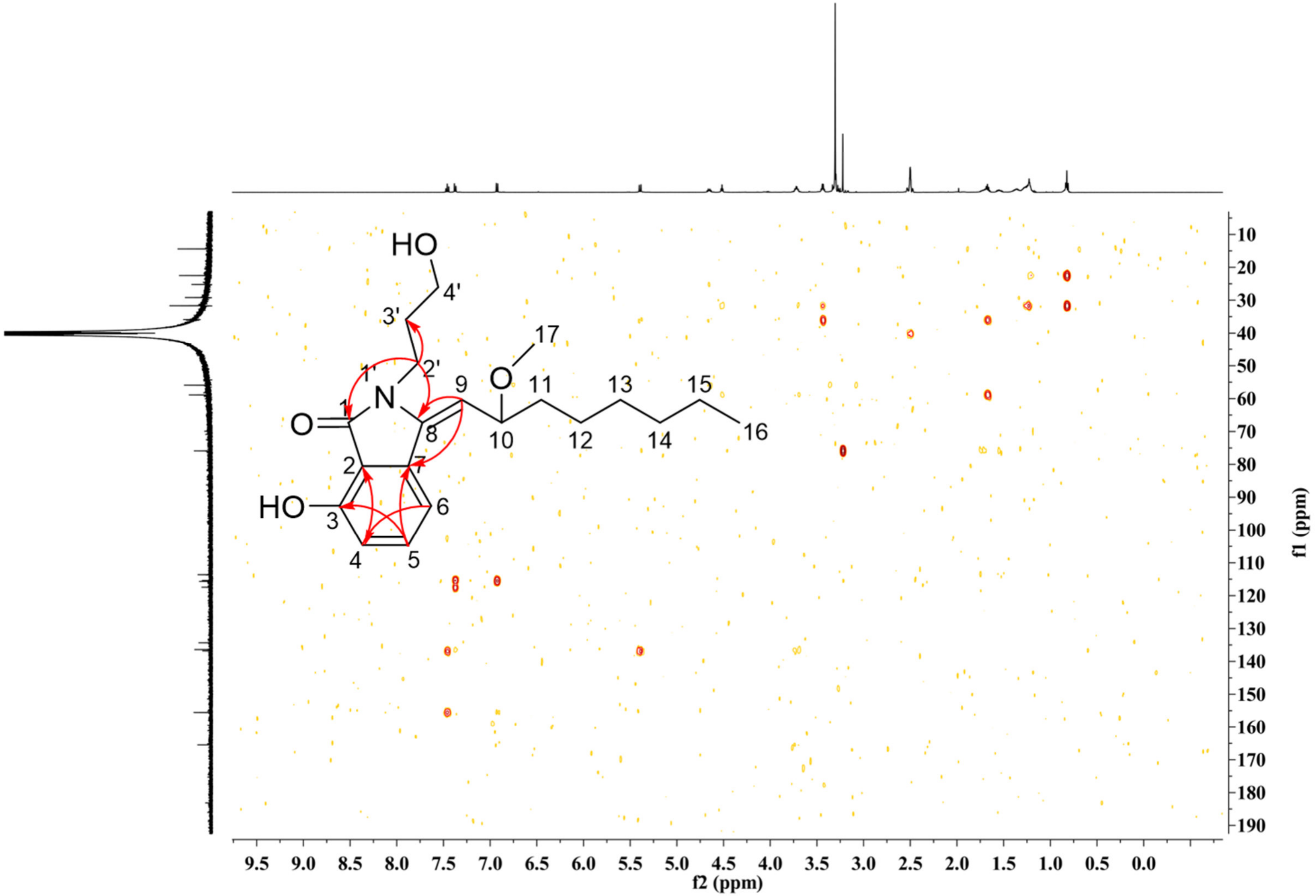

Figure S42. HMBC spectrum of roquesalin G (**9**) in DMSO-d_6.

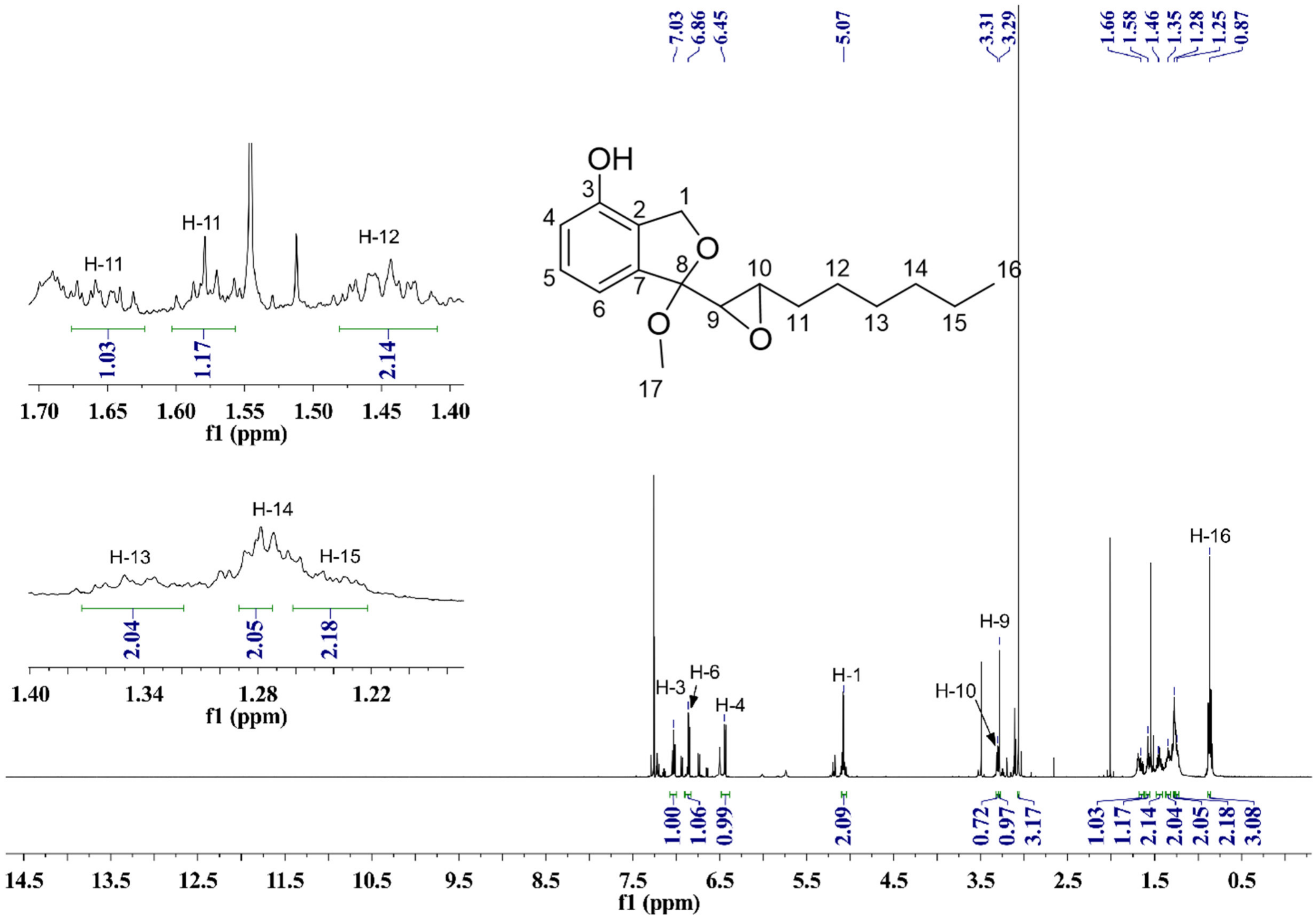

Figure S43. ^{1}H NMR spectrum of roquesalin H (**10**) in $CDCl_3$ (500 MHz).

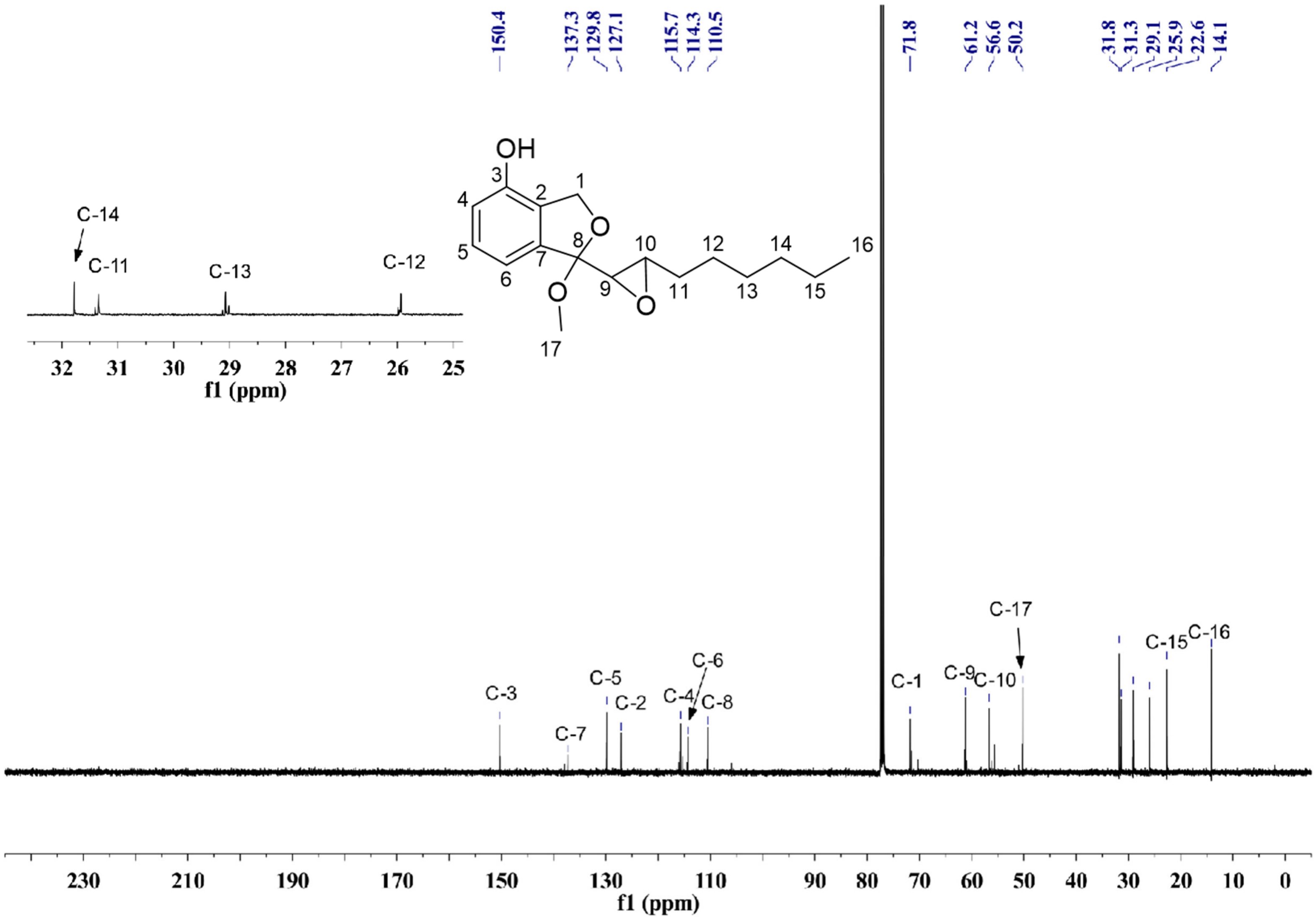

Figure S44. ^{13}C NMR spectrum of roquesalin H (**10**) in $CDCl_3$ (125 MHz).

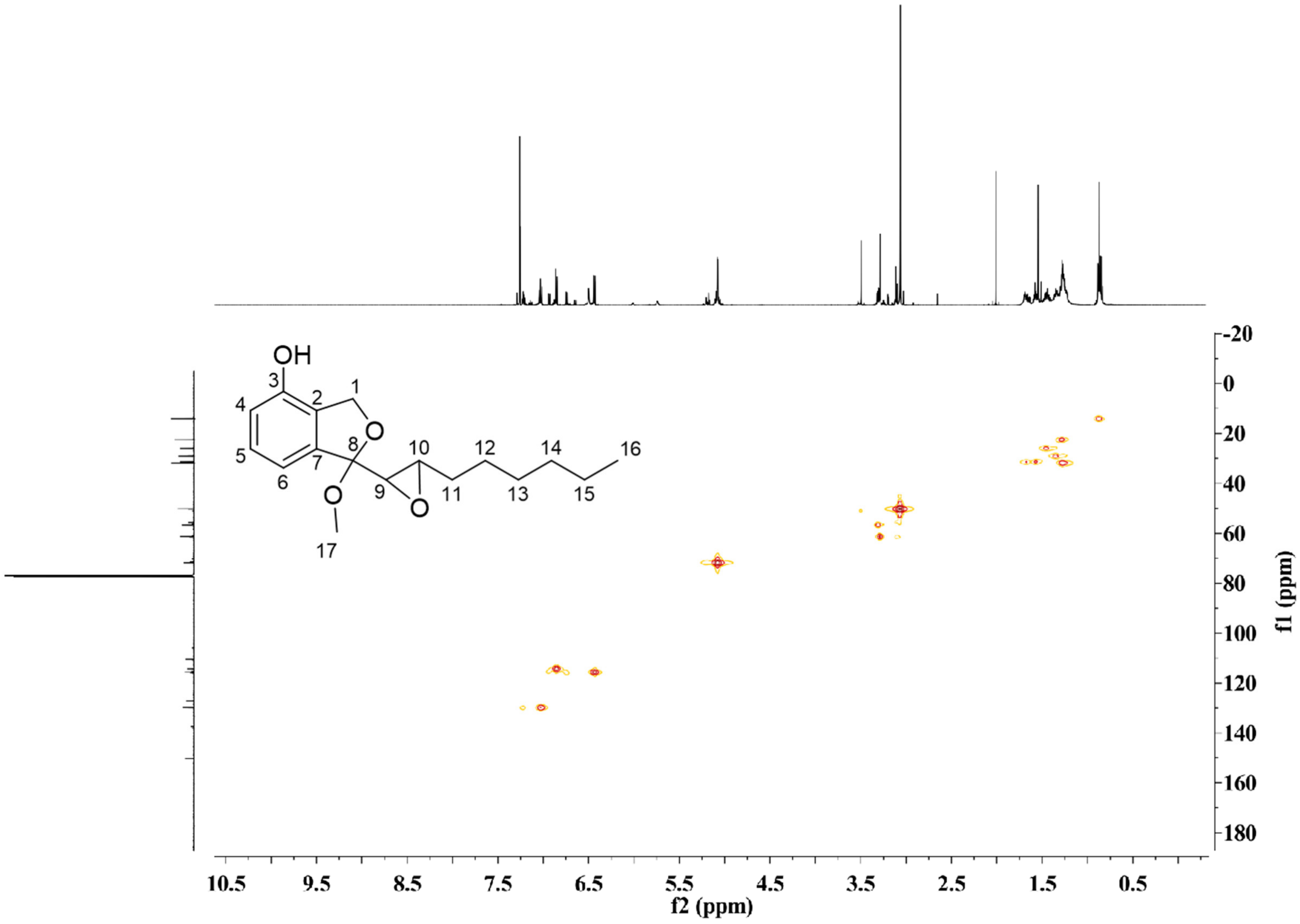

Figure S45. HSQC spectrum of roquesalin H (**10**) in $CDCl_3$.

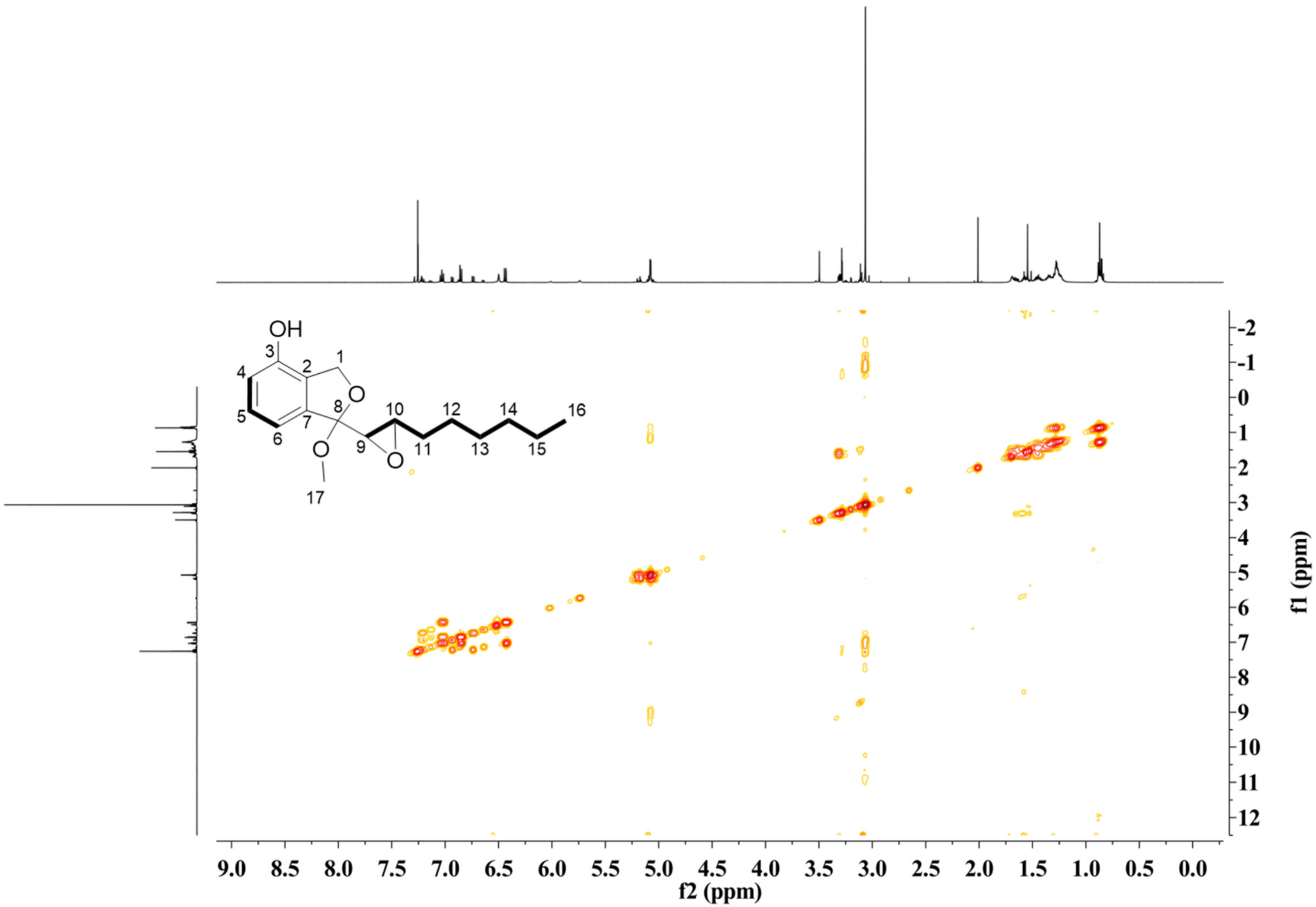

Figure S46. ^1H-^1H COSY spectrum of roquesalin H (**10**) in $CDCl_3$.

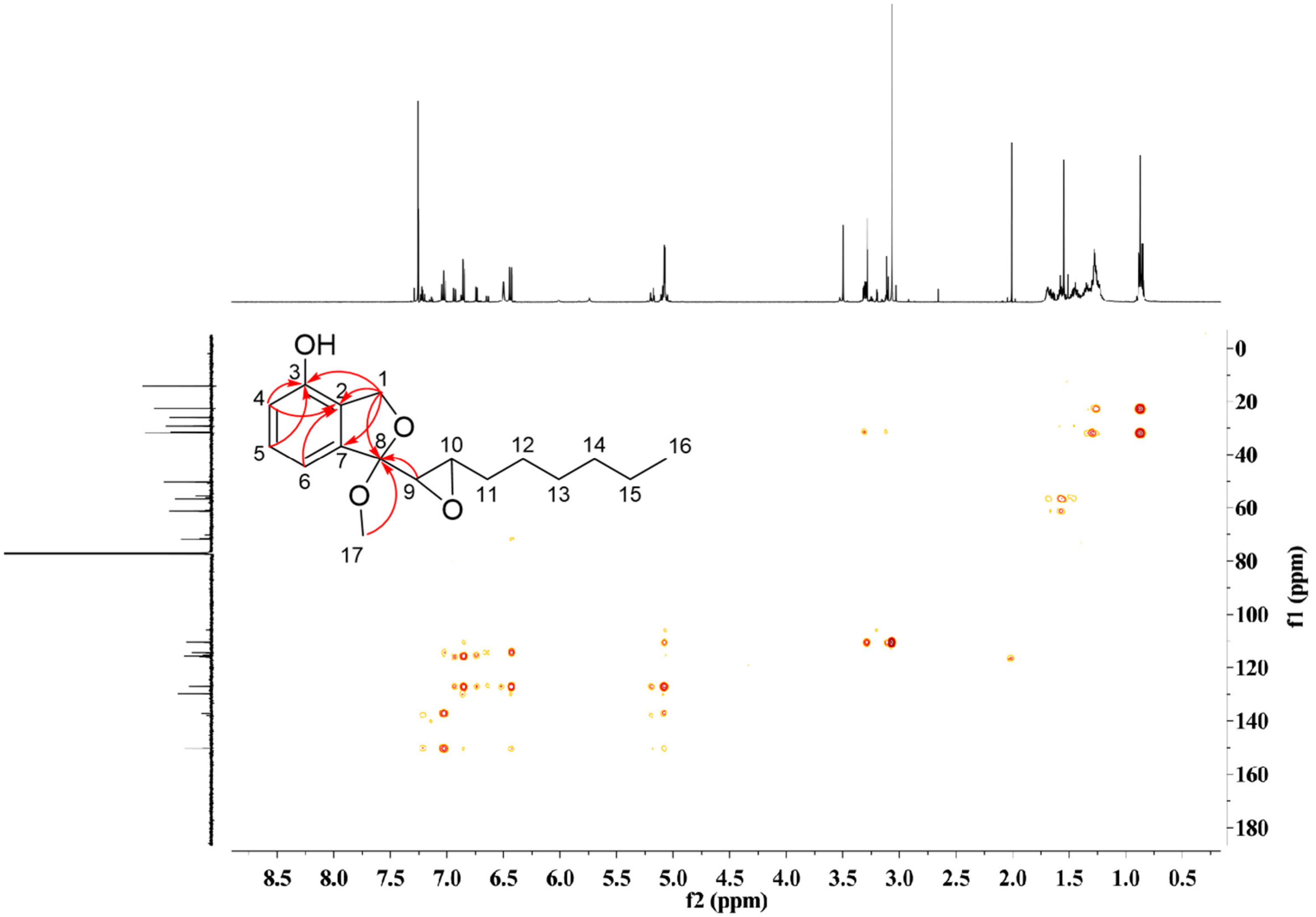

Figure S47. HMBC spectrum of roquesalin H (**10**) in $CDCl_3$.

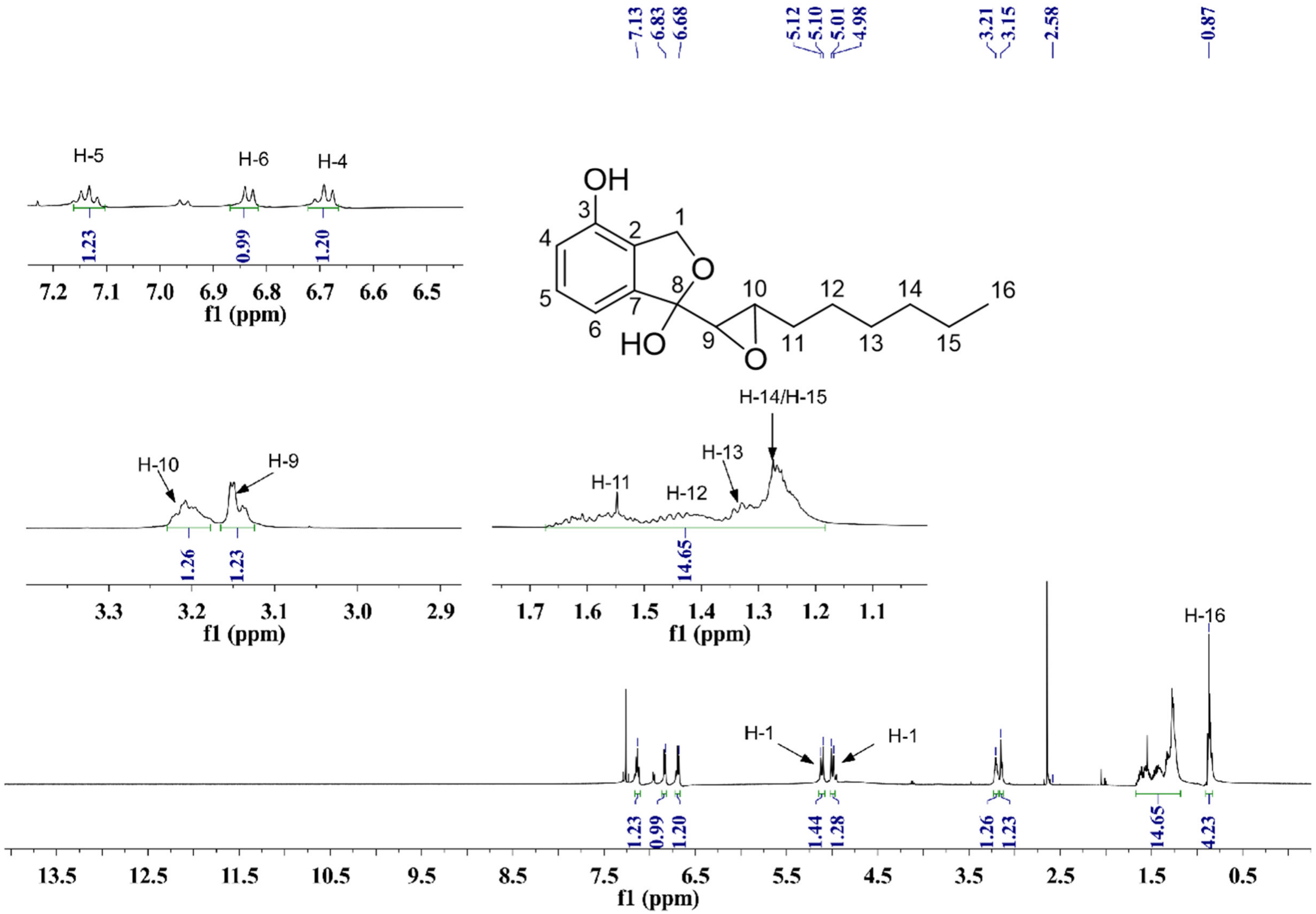

Figure S48. ^{1}H NMR spectrum of roquesalin I (**12**) in $CDCl_3$ (500 MHz).

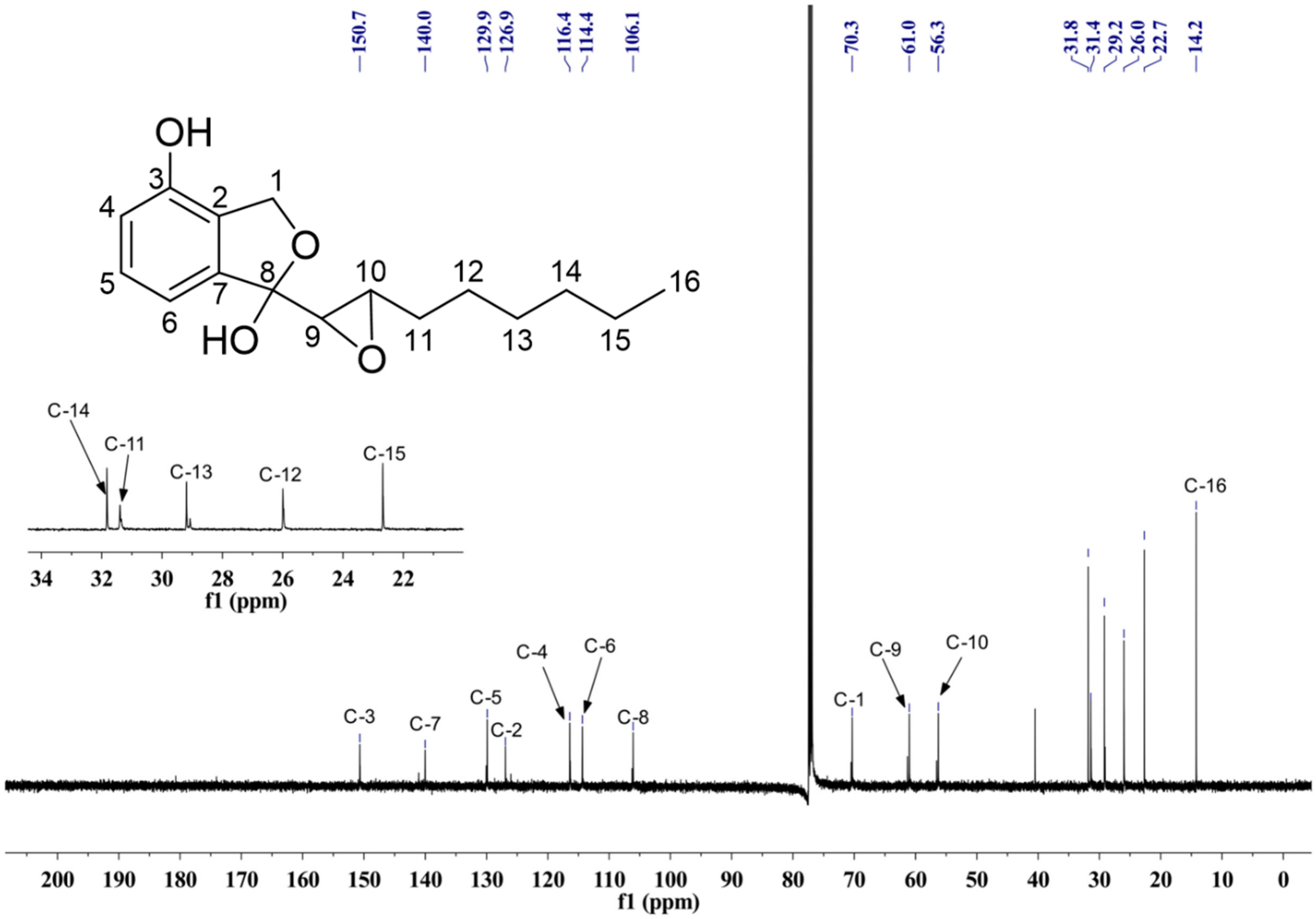

Figure S49. ^{13}C NMR spectrum of roquesalin I (**12**) in $CDCl_3$ (125 MHz).

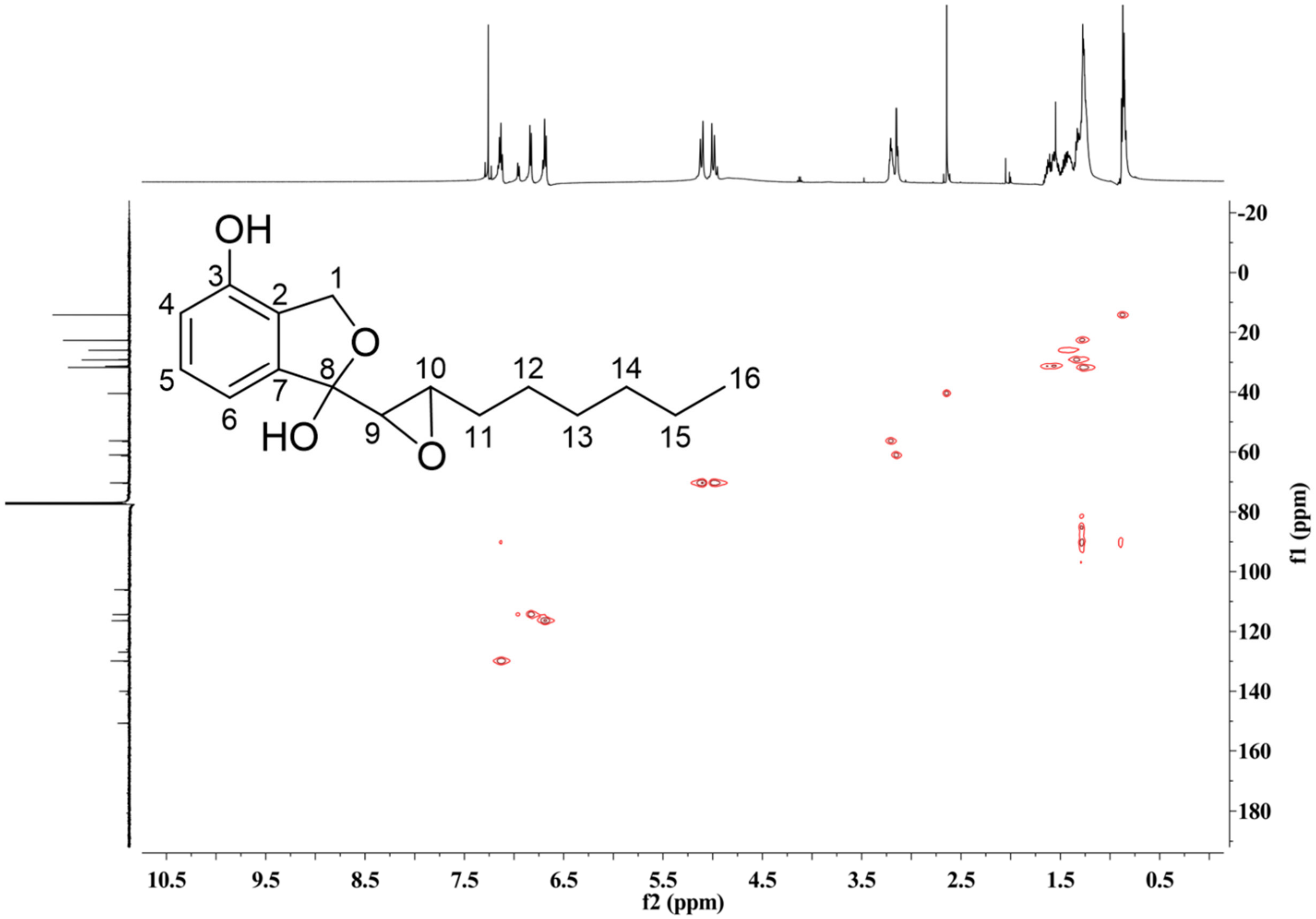

Figure S50. HSQC spectrum of roquesalin I (**12**) in $CDCl_3$.

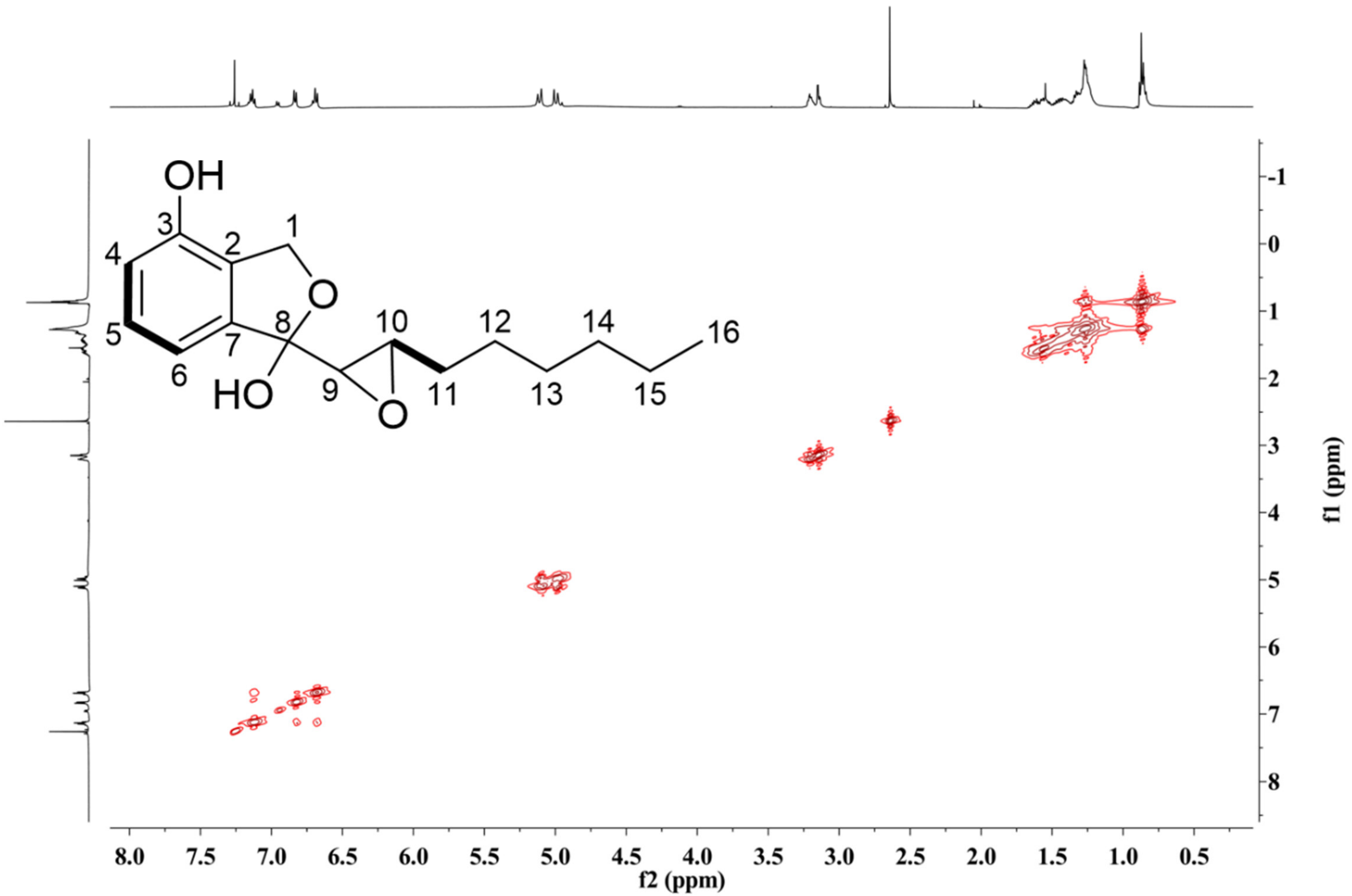

Figure S51. ^1H-^1H COSY spectrum of roquesalin I (**12**) in $CDCl_3$.

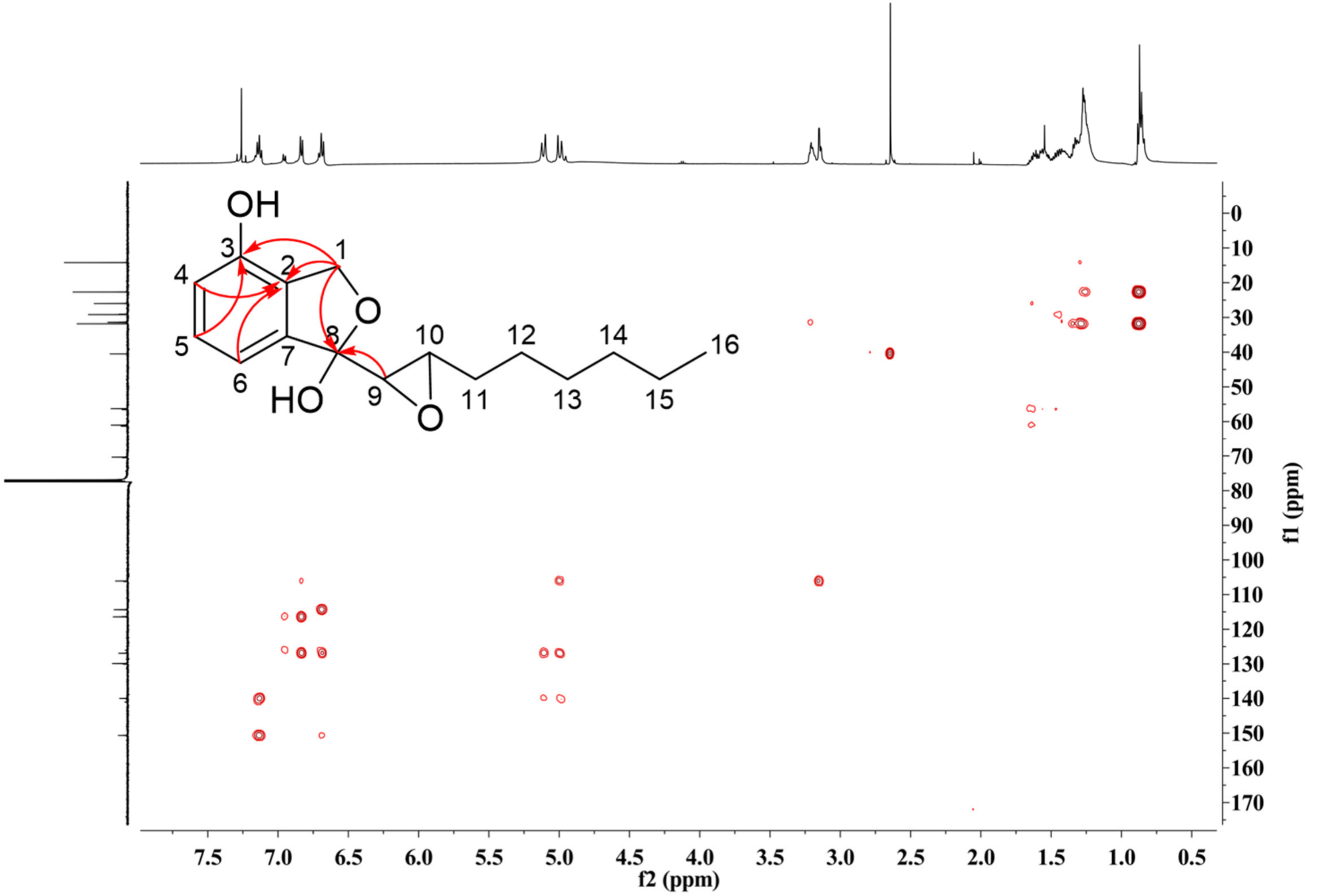

Figure S52. HMBC spectrum of roquesalin I (**12**) in $CDCl_3$.

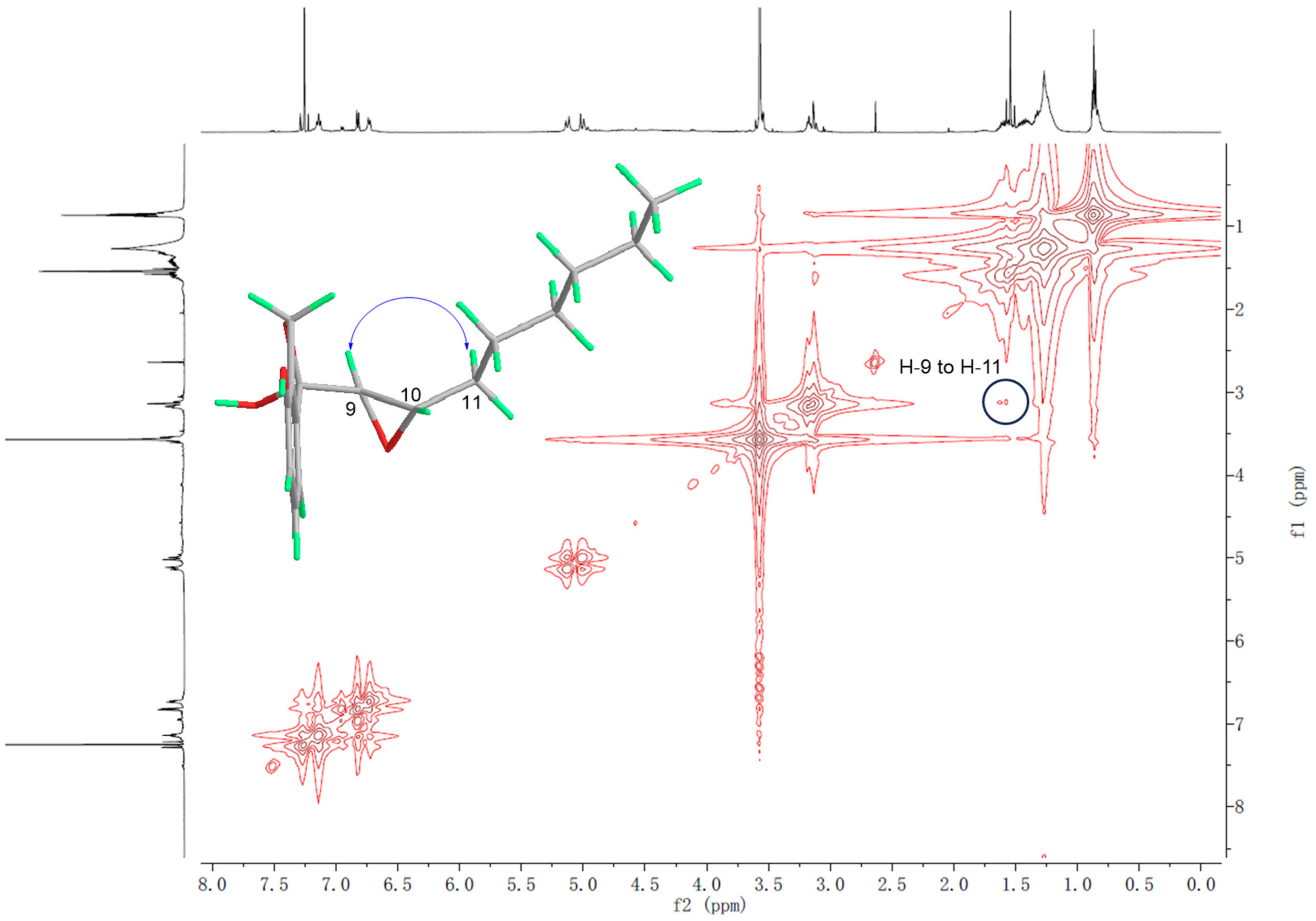

Figure S53. NOESY spectrum of roquesalin I (**12**) in $CDCl_3$.

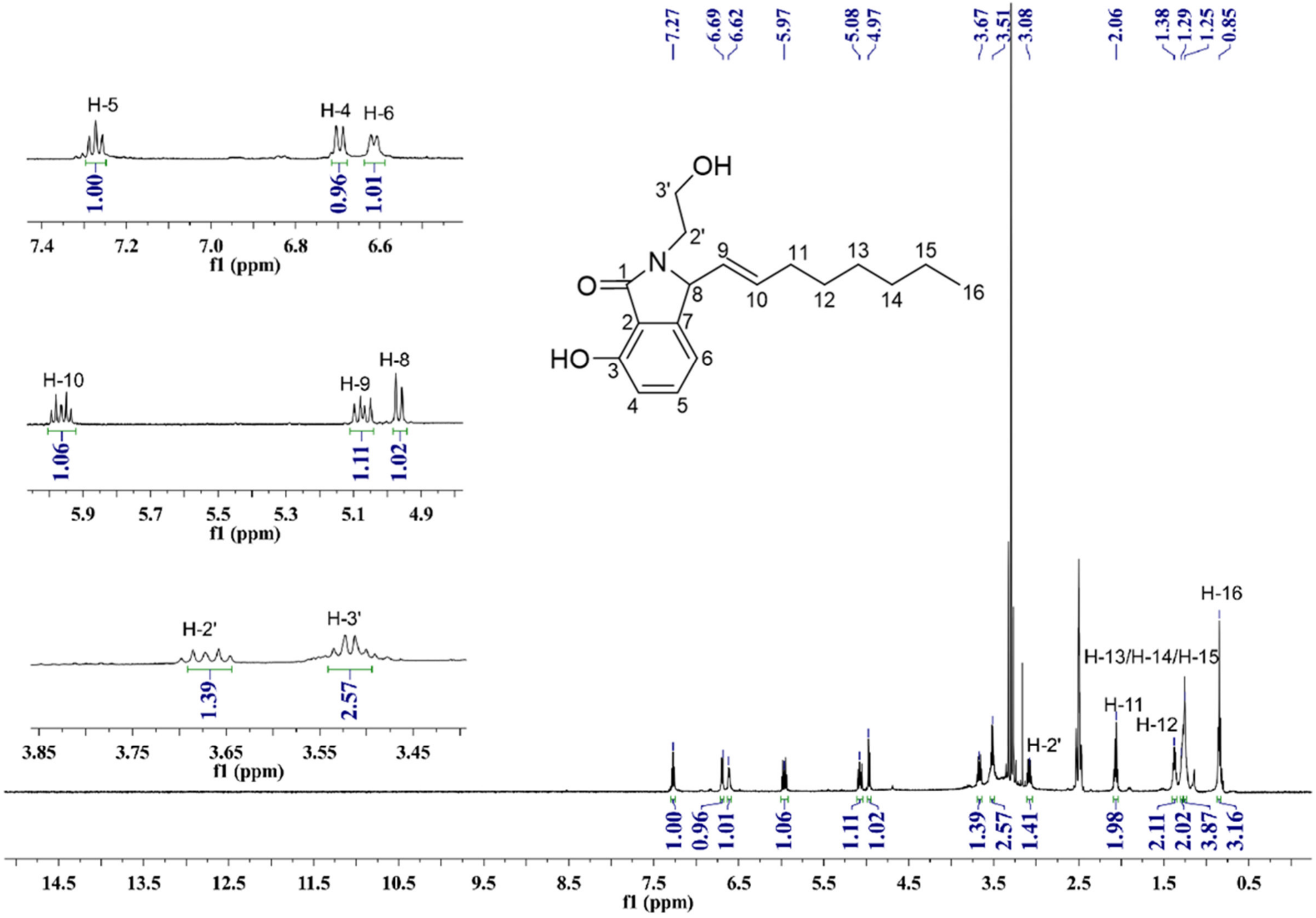

Figure S54. ^{1}H NMR spectrum of roquesalin J (**14**) in DMSO-d_6 (500 MHz).

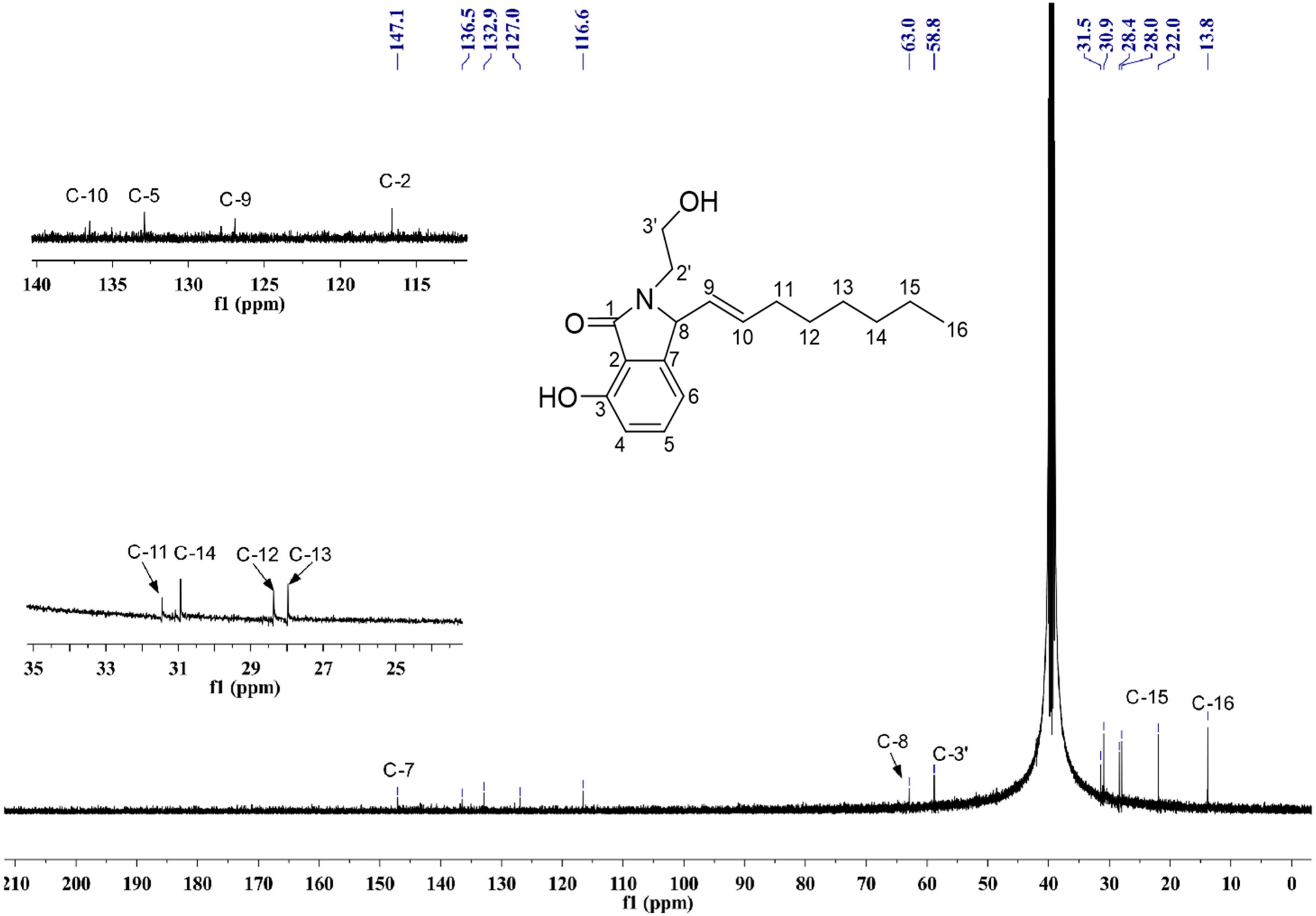

Figure S55. ^{13}C NMR spectrum of roquesalin J (**14**) in DMSO-d_6 (125 MHz).

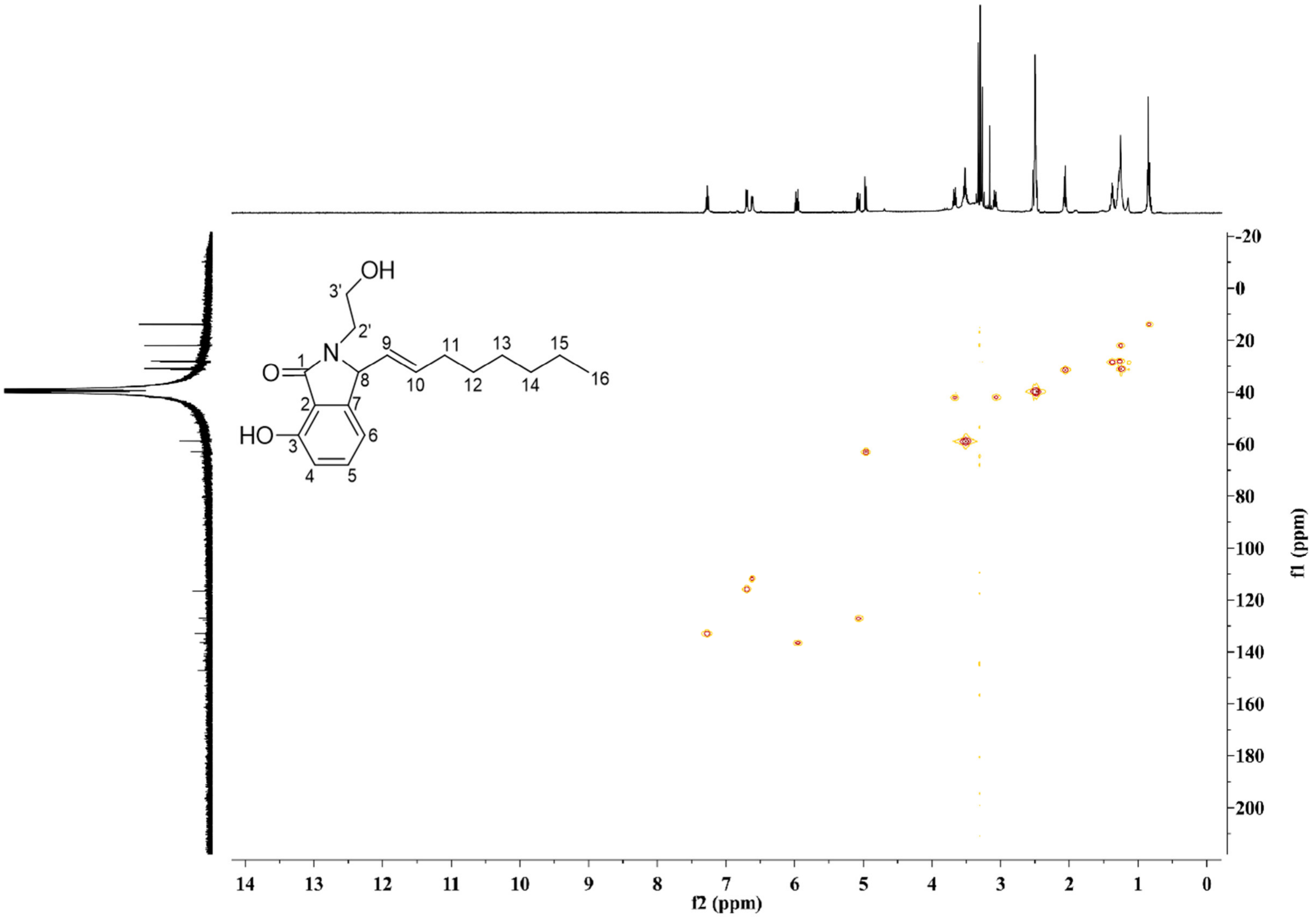

Figure S56. HSQC spectrum of roquesalin J (**14**) inDMSO-d_6.

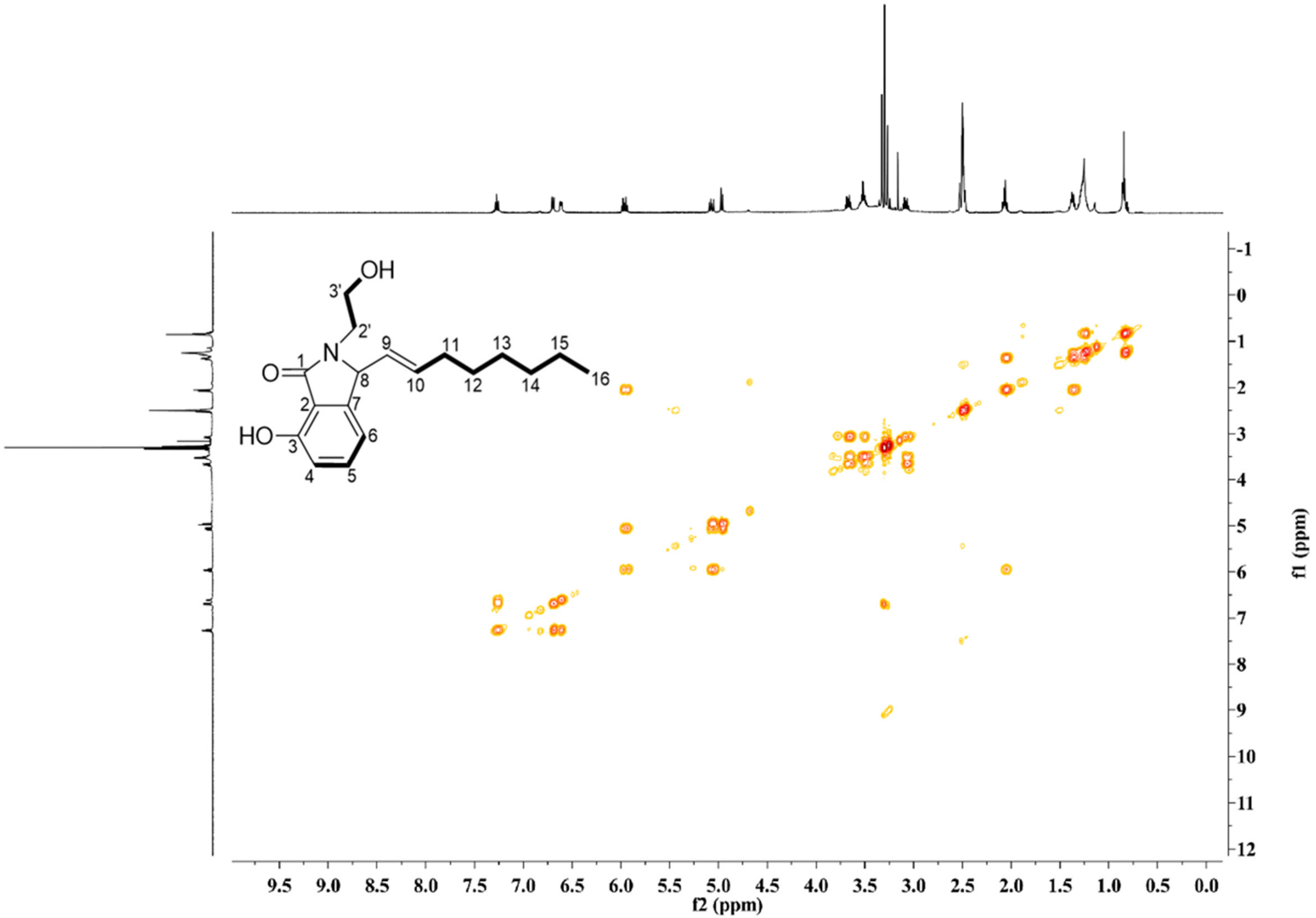

Figure S57. ^{1}H-^{1}H COSY spectrum of roquesalin J (**14**) in DMSO-d_6.

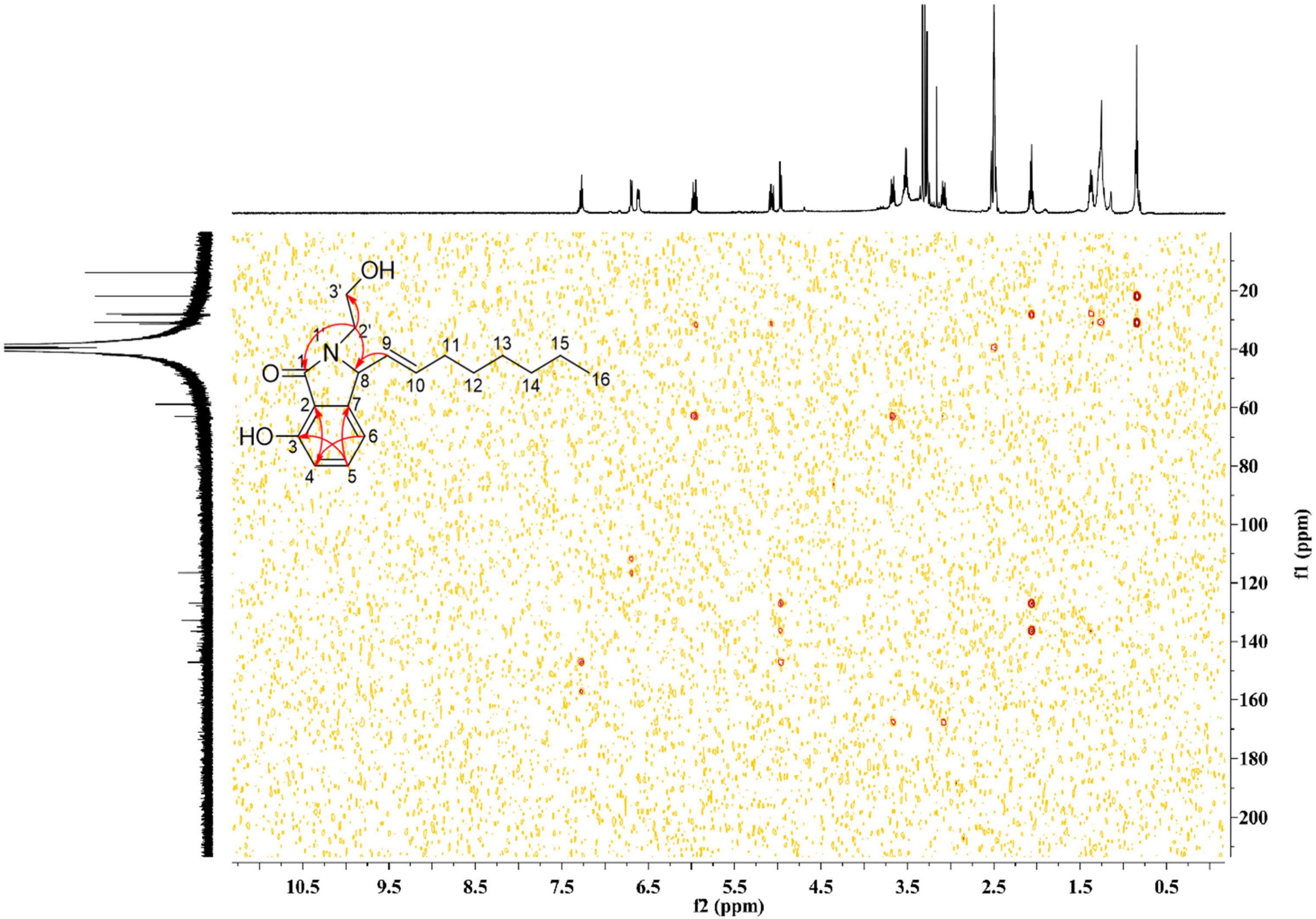

Figure S58. HMBC spectrum of roquesalin J (**14**) in DMSO-d_6.

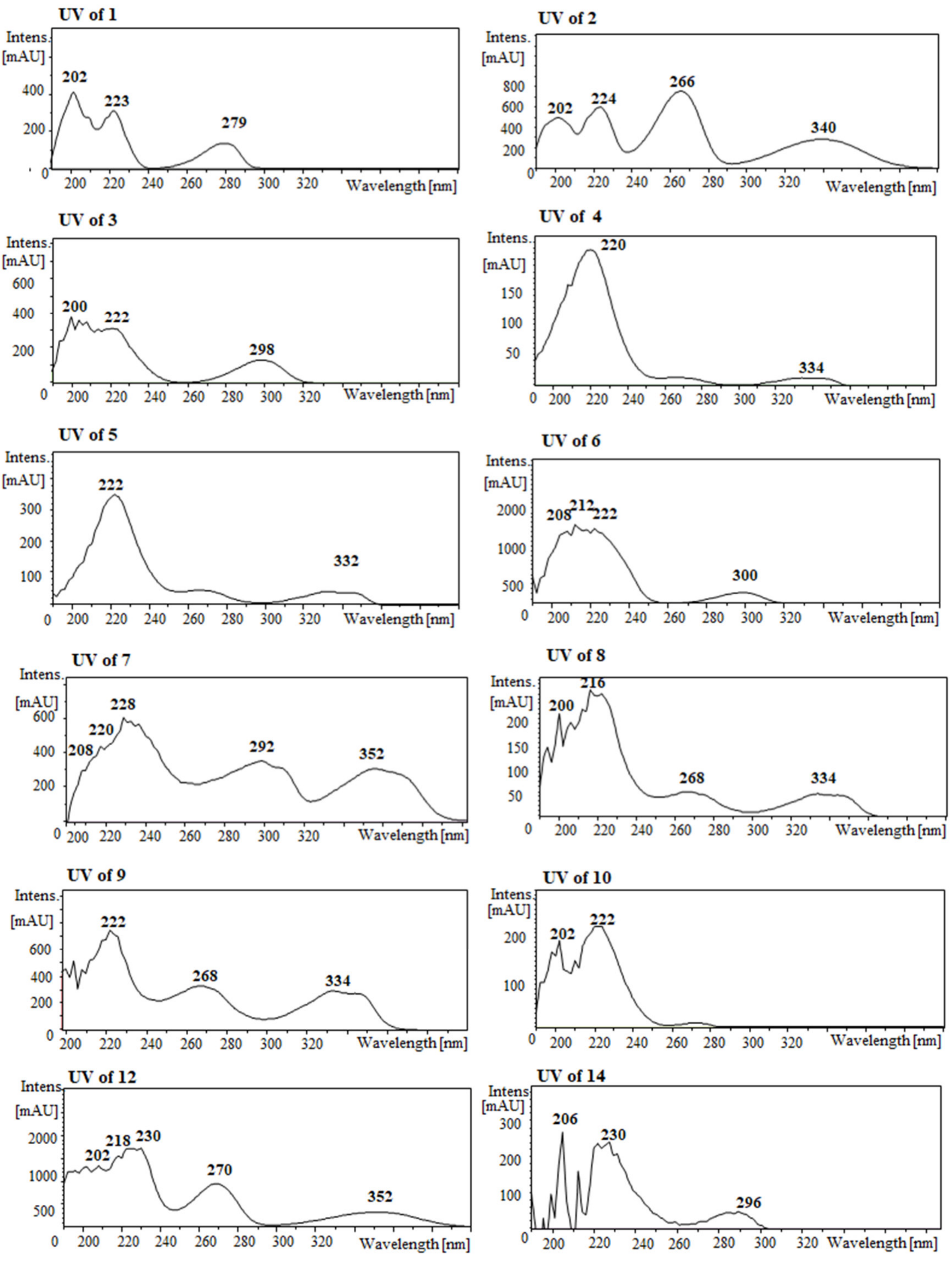

Figure S59. The UV spectra of the identified compounds.

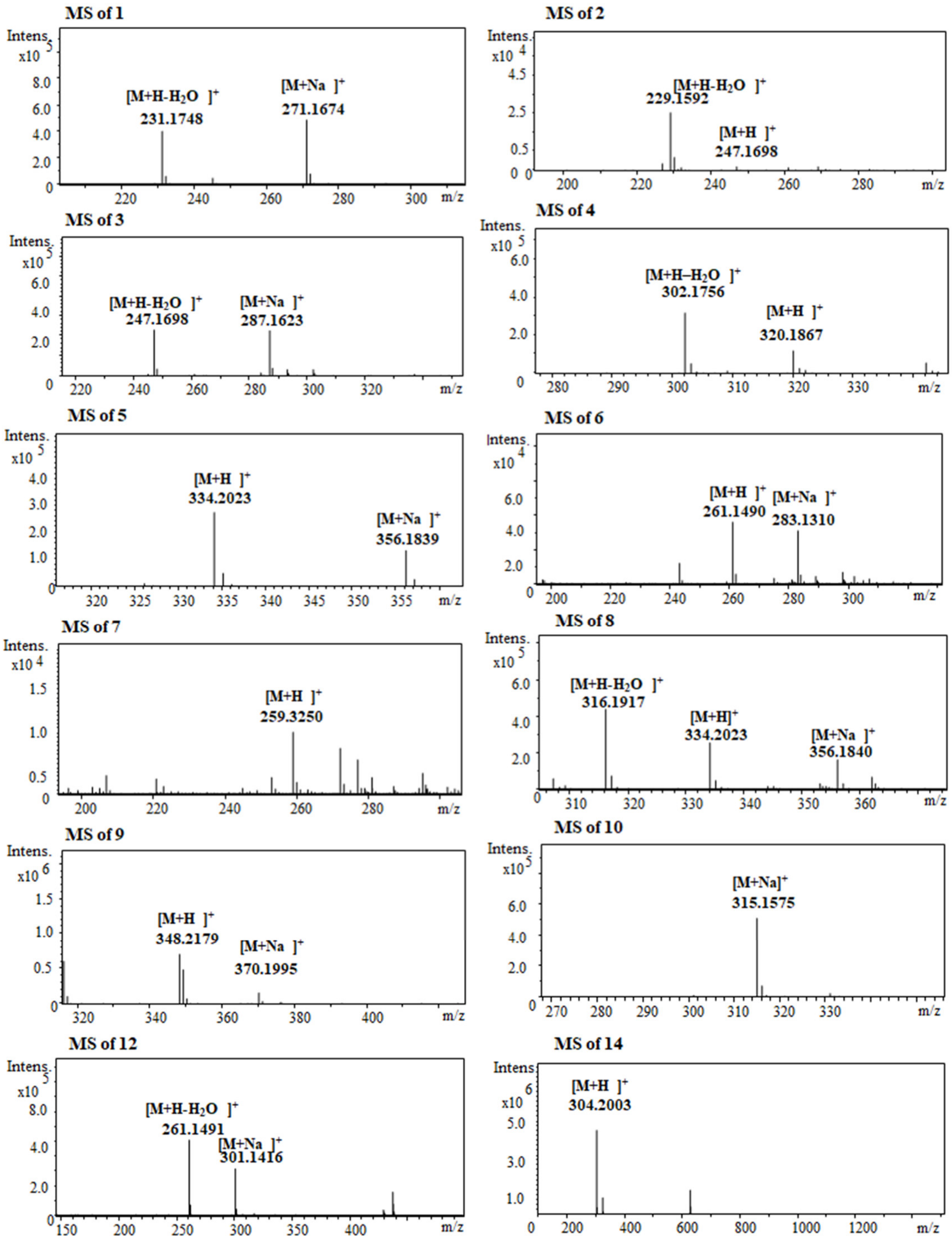

Figure S60. The mass spectra of the identified compounds.

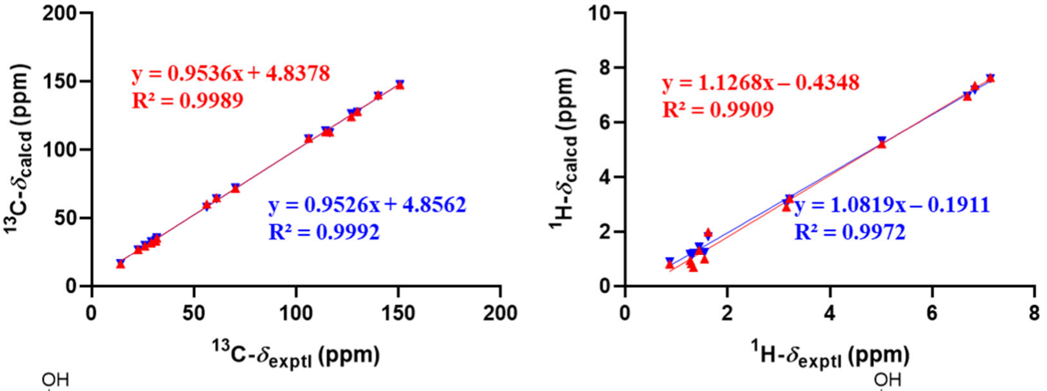

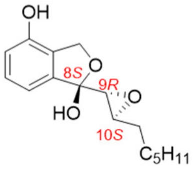

	(8*S*,9*R*,10*S*)-**12**		(8S,9*S*,10*R*)-**12**	
	^{13}C	^{1}H	^{13}C	^{1}H
**MAE**	2.70	0.33	2.52	0.19
**CMAE**	1.30	0.17	1.00	0.10
**DP4+**	0.0%		100.0%	

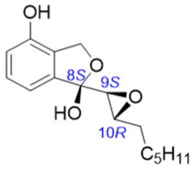

Figure S61. Linear regression analysis of experimental and calculated NMR chemical shifts of isomers of **12**.

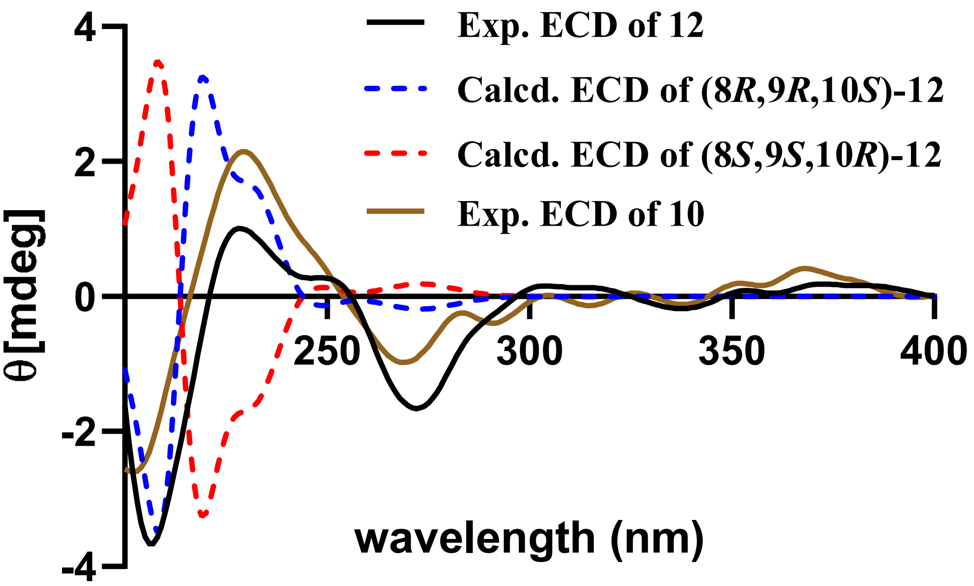

Figure S62. The calculated ECD spectra of (8*S*,9*S*,10*R*)- and (8*R*,9*R*,10*S*)-**12**, and experimental ECD spectra of **10** and **12**.

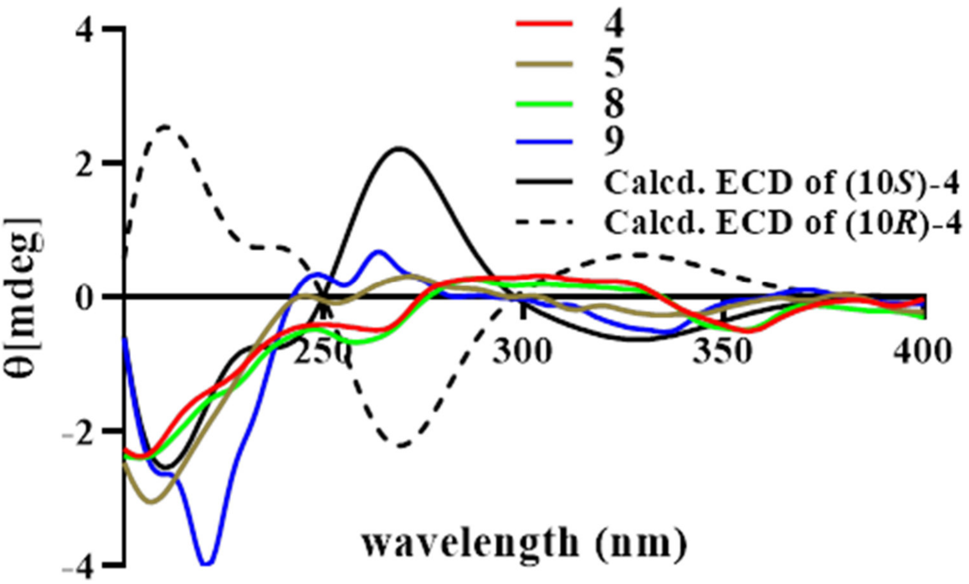

Figure S63. The experimental spectra of **4**, **5**, **8**, and **9**, and calculated ECD spectra of **4**.

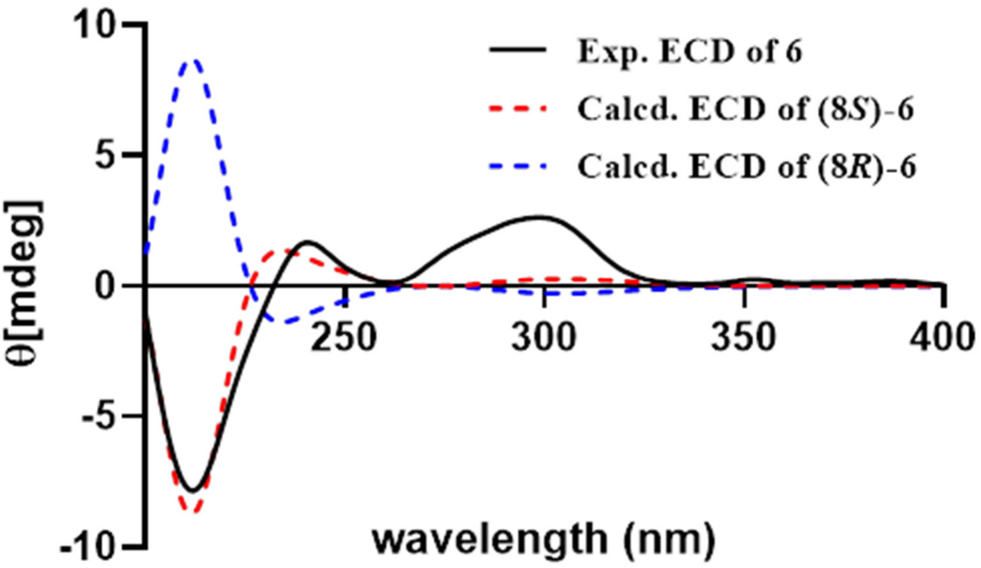

Figure S64. The experimental spectrum of **6**, and calculated ECD spectra of **6**.

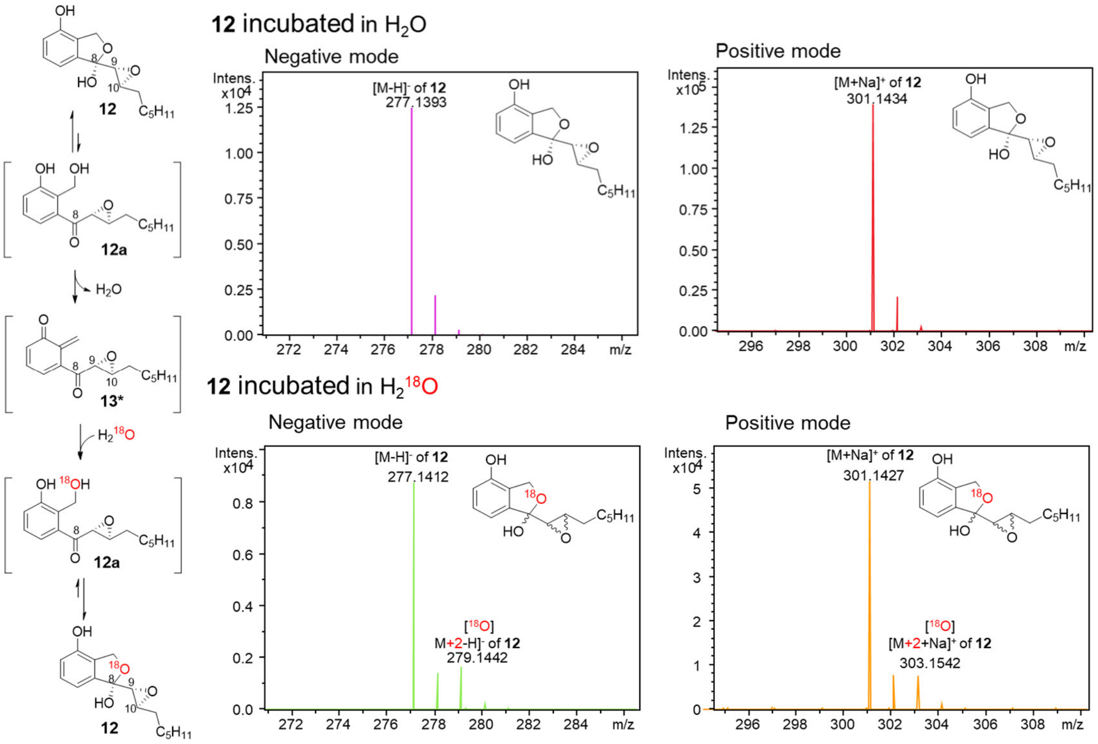

Figure S65. MS analysis of **12** after incubation in H_2O or $H_2^{18}O$ on both positive and negative modes.

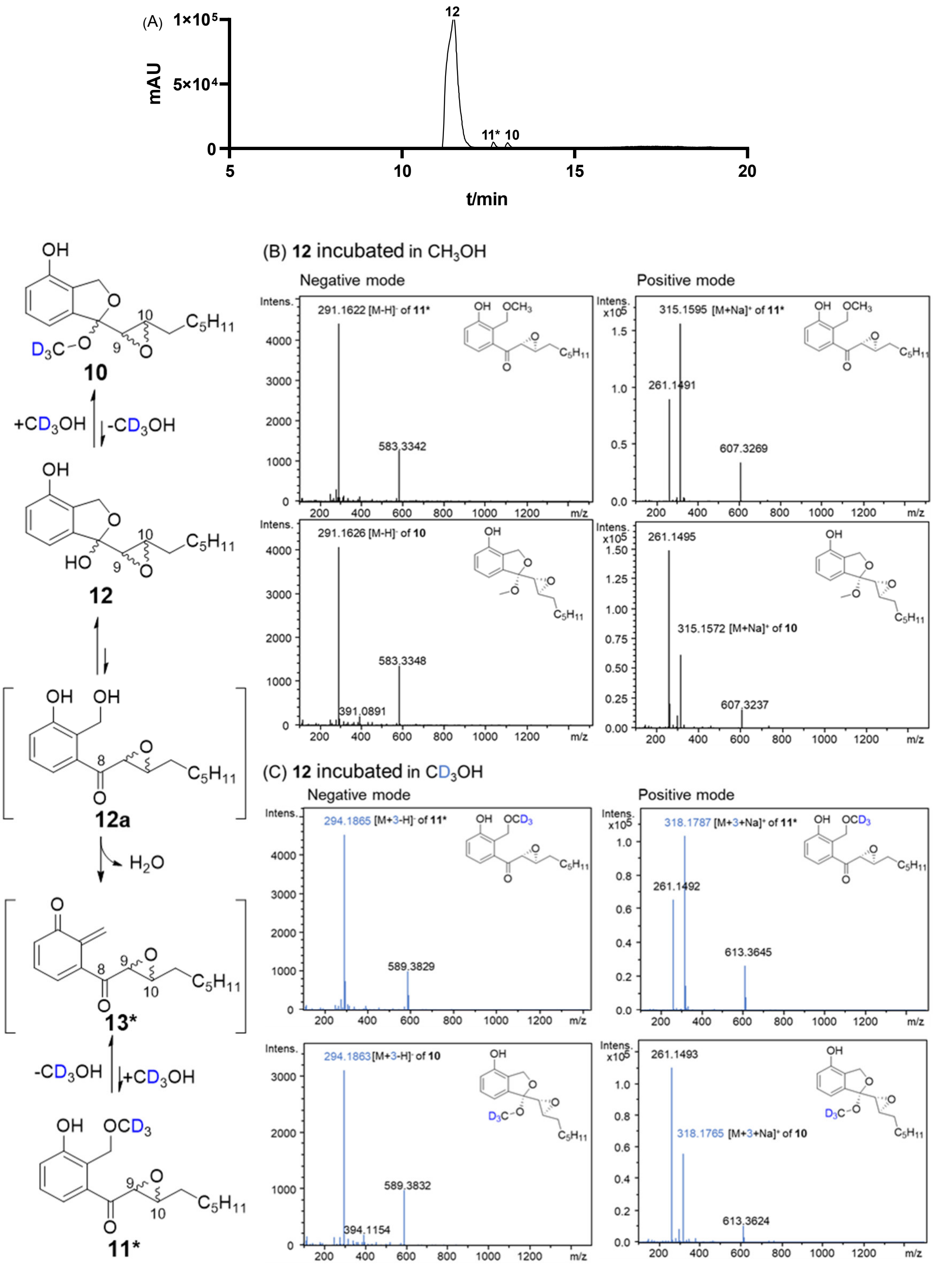

Figure S66. Conversion of **12** to **10** and **11***.

LC-MS chromatogram of **12** after incubation in CH_3OH at room temperature for 16h (A). MS spectra of **10** and **11*** after cultivation of **12** in CH_3OH (B) or CD_3OH (C) on both positive and negative modes.

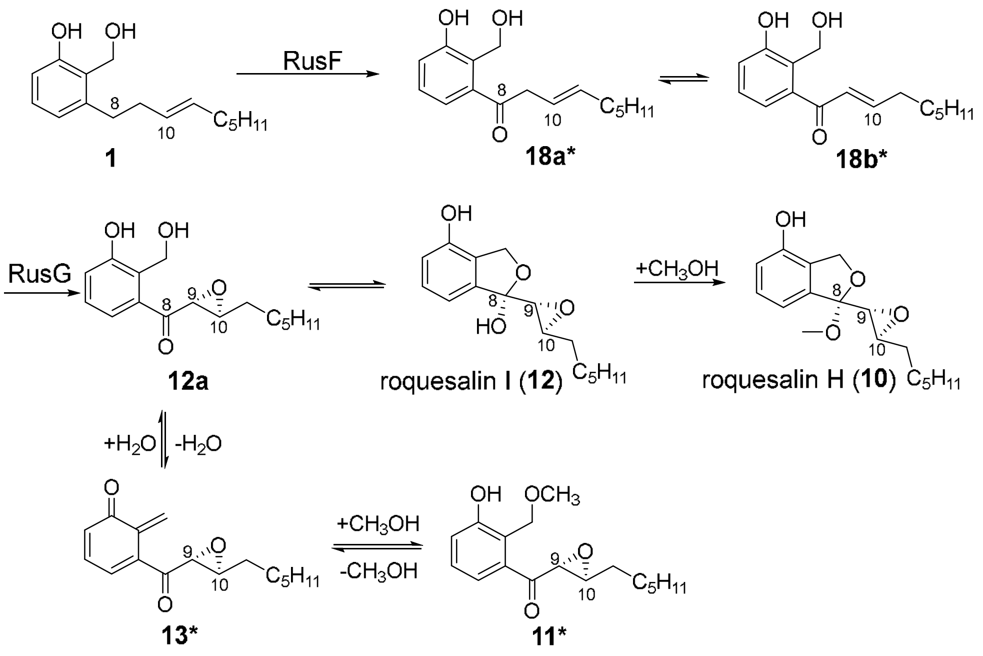

Figure S67. Proposed shunt pathway in the absence of RusE.

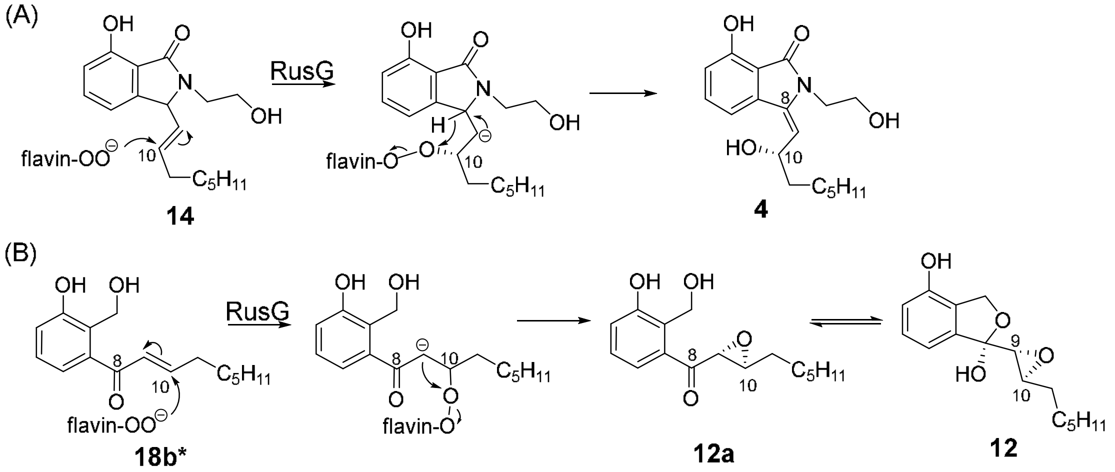

Figure S68. Proposed mechanisms of the RusG-catalyzed oxidations.

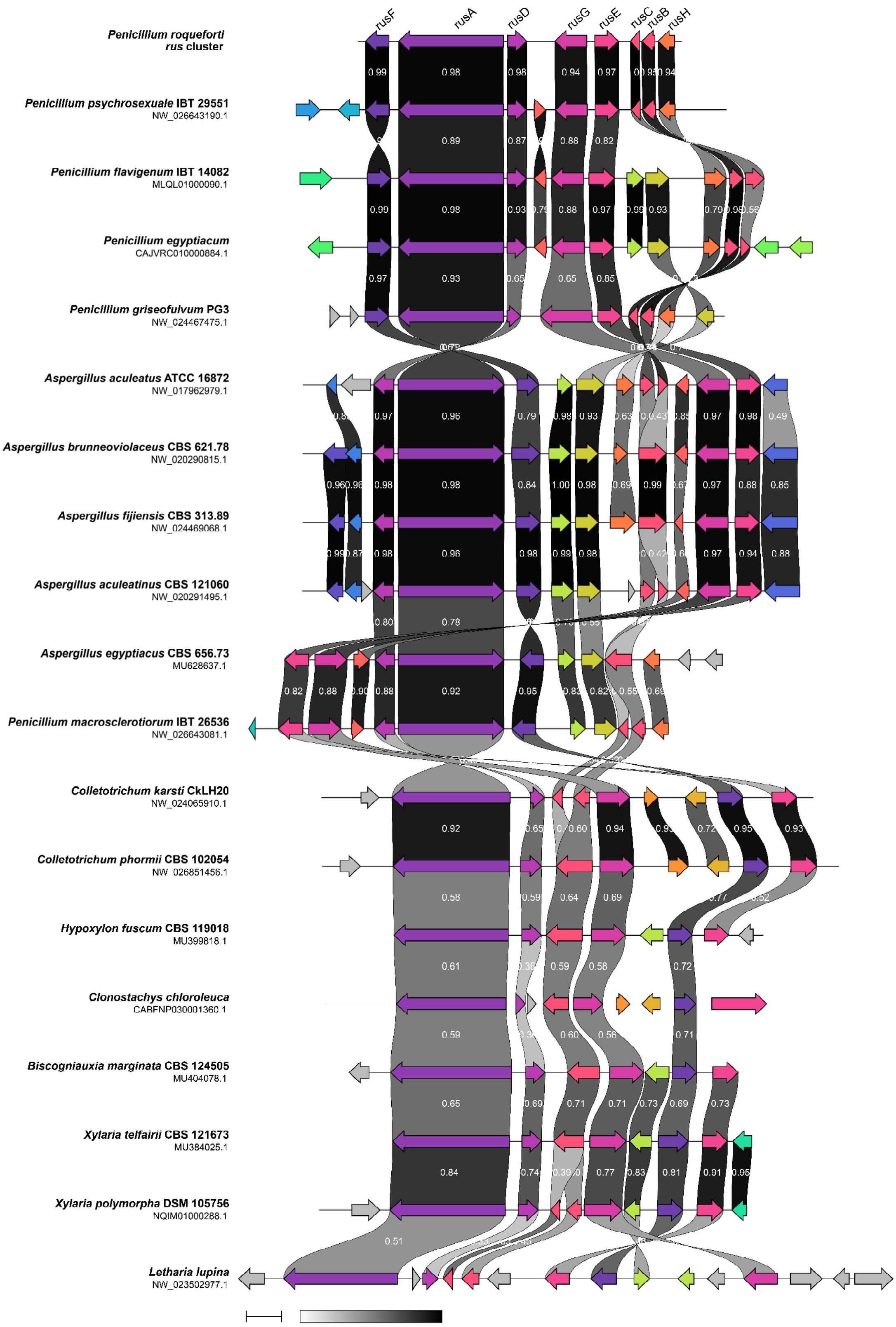

Figure S69. Representative homologous BGCs of the *rus* cluster identified in other fungi by using cblaster.

Supplementary References

1 K. R. Oldenburg, K. T. Vo, S. Michaelis and C. Paddon, *Nucleic Acids Res.*, 1997, **25**, 451.

2 C. L. M. Gilchrist, T. J. Booth, B. van Wersch, L. van Grieken, M. H. Medema and Y.-H. Chooi, *Bioinformatics Advances*, 2021, **1**, vbab016.

3 M. van den Belt, C. Gilchrist, T. J. Booth, Y.-H. Chooi, M. H. Medema and M. Alanjary, *BMC Bioinformatics*, 2023, **24**, 181.

4 D. A. Yee and Y. Tang, *Methods Mol. Biol. (N. Y., NY, U. S.)*, 2022, **2489**, 41.

5 Y. Yuan, S. Cheng, G. Bian, P. Yan, Z. Ma, W. Dai, R. Chen, S. Fu, H. Huang, H. Chi, Y. Cai, Z. Deng and T. Liu, *Nature Catal.*, 2022, **5**, 277.

6 J. Nies, H. Ran, V. Wohlgemuth, W. B. Yin and S.-M. Li, *Org. Lett.*, 2020, **22**, 2256.

7 D. J. Janzen, J. Zhou and S.-M. Li, *Org. Lett.*, 2023, **25**, 6311.

8 P. Pracht, F. Bohle and S. Grimme, *Phys. Chem. Chem. Phys.*, 2020, **22**, 7169.

9 T. Bruhn, A. Schaumlöffel, Y. Hemberger and G. Bringmann, *Chirality*, 2013, **25**, 243.

10 N. Grimblat, M. M. Zanardi and A. M. Sarotti, *J. Org. Chem.*, 2015, **80**, 12526.

11 R. Yang, J. Feng, H. Xiang, B. Cheng, L.-D. Shao, Y.-P. Li, H. Wang, Q.-F. Hu, W.-L. Xiao, Y. Matsuda and W.-G. Wang, *J. Am. Chem. Soc.*, 2023, **145**, 11293.

12 N. M. O'Boyle, M. Banck, C. A. James, C. Morley, T. Vandermeersch and G. R. Hutchison, *J. Cheminform.*, 2011, **3**, 33.

13 C. Bannwarth, S. Ehlert and S. Grimme, *J. Chem. Theory Comput.*, 2019, **15**, 1652.

14 M. R. Green and J. Sambrook, *Molecular cloning: a laboratory manual*, Cold Spring Harbor Laboratory Press, Cold Spring Harbor, New York, 2012, 4th.

15 C. Bond, Y. Tang and L. Li, *Fungal Genet. Biol.*, 2016, **89**, 52.

16 Y. M. Chiang, M. Ahuja, C. E. Oakley, R. Entwistle, A. Asokan, C. Zutz, C. C. Wang and B. R. Oakley, *Angew. Chem., Int. Ed.*, 2016, **55**, 1662.
